# Metallized porphyrin-based covalent organic frameworks with orbital-hybridized conduction channels for continuous-flow adsorption–photocatalytic uranium extraction from seawater

**DOI:** 10.1039/d6sc00048g

**Published:** 2026-08-10

**Authors:** Bin Zuo, Ruoyu Wang, Guoze Yan, Jiayu Zuo, Likun Pan, Dong Jiang, Yusuke Yamauchi, Xingtao Xu

**Affiliations:** a Marine Science and Technology College, Zhejiang Ocean University Zhoushan 316022 China xingtao.xu@zjou.edu.cn; b School of Energy Science and Engineering, Harbin Institute of Technology Harbin Heilongjiang 150001 China; c Shanghai Key Laboratory of Magnetic Resonance, School of Physics and Electronic Science, Institute of Magnetic Resonance and Molecular Imaging in Medicine, East China Normal University Shanghai 200241 China; d Australian Institute for Bioengineering and Nanotechnology (AIBN), The University of Queensland Brisbane QLD 4072 Australia y.yamauchi@uq.edu.au; e Department of Materials Process Engineering, Graduate School of Engineering, Nagoya University Nagoya 464-8603 Japan; f Department of Chemical and Biomolecular Engineering, Yonsei University 50 Yonsei-ro, Seodaemun-gu Seoul 03722 South Korea

## Abstract

Adsorption–photocatalytic uranium extraction from seawater is emerging as a highly attractive route for sustainable nuclear-fuel supply. However, its efficiency is often limited by a kinetic mismatch between uranyl adsorption (mass transport) and photochemical reduction (electron transfer). Herein, we report a family of metallized porphyrin-based covalent organic frameworks (M–Por-COFs-AO, M = Co, Ni, Cu) in which metal–ligand coordination constructs well-defined π–d hybridized conductive channels. These channels are designed to promote coordinated ion diffusion and electron migration, thereby enabling a balanced adsorption–photocatalysis cycle. Among the series, Ni–Por-COF-AO exhibits the most favorable transport characteristics, delivering an ultrahigh affinity (*K*_d_ = 1.2 × 10^9^ mL g^−1^), a superior U/V selectivity ratio (*S*_U/V_ = 62.7), and a high uranium extraction capacity of 516.9 mg g^−1^ from 20 ppm uranyl solution. Under visible light in a continuous-flow photochemical system, it achieves a uranium extraction capacity of 21.2 mg g^−1^ from natural seawater within seven days without sacrificial agents. Mechanistic analyses reveal that the hybridization between the transition-metal e–g orbitals and porphyrin π-systems generates delocalized conductive pathways, enabling directional charge migration and selective ion sieving. This work establishes an orbital-hybridized channel engineering paradigm for COFs and demonstrates a materials-to-device integrated strategy that achieves balanced ion-electron transport for efficient and sustainable uranium recovery from seawater.

## Abstract 2

Metallized porphyrin-based covalent organic frameworks have been designed for adsorption–photocatalytic uranium extraction from seawater.

## Graphical abstract

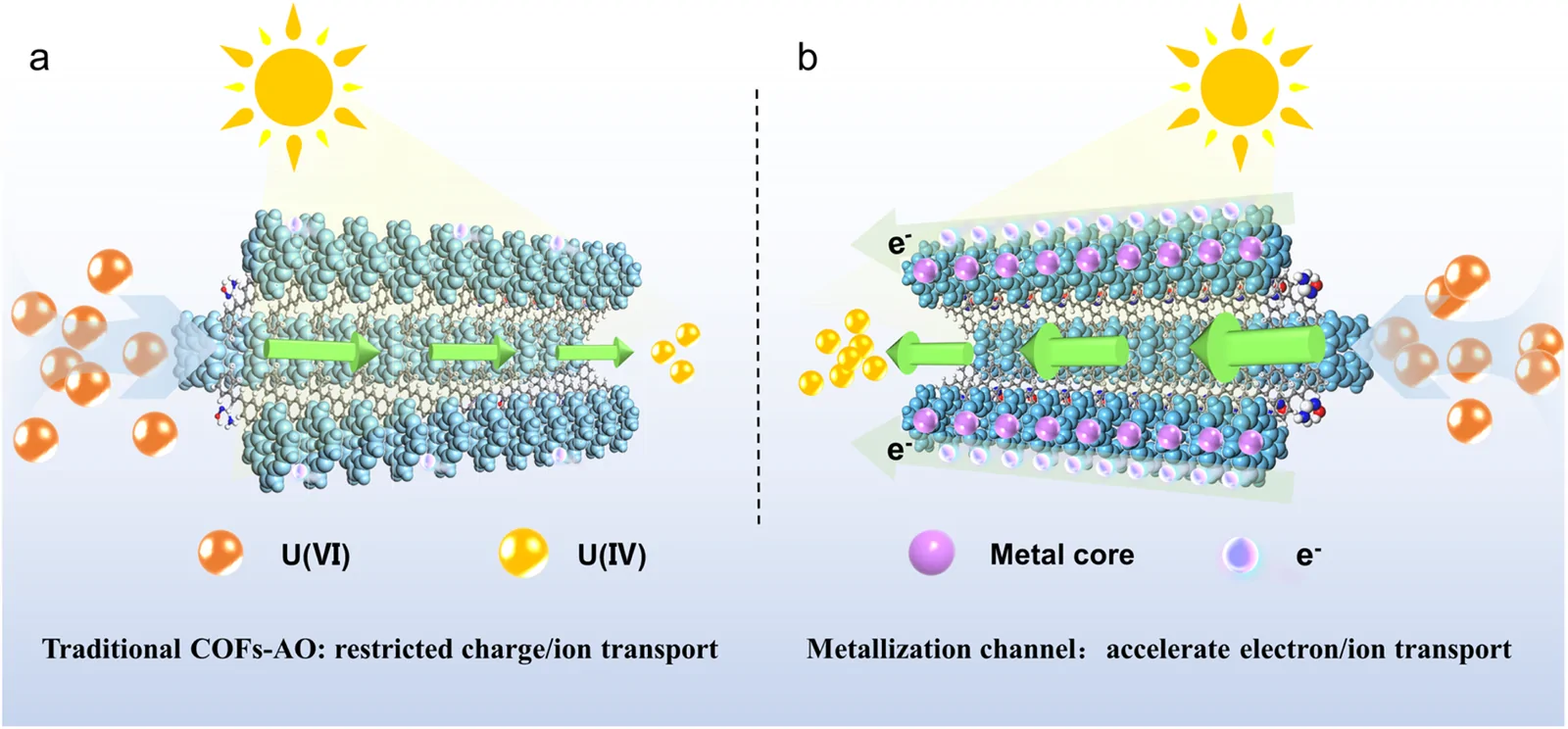

## 1 Introduction

Uranium is an essential fuel for the nuclear industry and is vital for achieving a sustainable and low-carbon energy transition.^1–3^ However, terrestrial uranium ores are limited and unevenly distributed, posing long-term supply risks.^4,5^ In contrast, the oceans contain an estimated 4.5 billion tons of dissolved uranium, representing an almost inexhaustible reserve.^4,5^ Yet, the extremely low uranium concentration (∼3.3 ppb), the abundance of competing ions (Na^+^, Ca^2+^, Mg^2+^), and extensive biofouling in seawater make practical recovery highly challenging.^6^ Therefore, developing advanced materials that offer high selectivity, fast adsorption kinetics, and efficient regeneration capability remains a pressing goal for sustainable uranium extraction.

To date, solvent extraction, membrane separation, adsorption and photocatalytic reduction have been proposed as effective strategies for uranium extraction.^7^ Among these approaches, photocatalytic reduction is particularly attractive due to its remarkable advantages of high efficiency, ease of operation, and low energy consumption. Numerous porous materials, including metal–organic frameworks (MOFs), porous organic polymers, porous aromatic frameworks (PAFs), and covalent organic frameworks (COFs), have been explored for the extraction of uranium.^8,9^ Among them, amidoxime-functionalized COFs (COFs-AO) have emerged as versatile platforms for uranium extraction from seawater, benefiting from their high selectivity, ordered porosity and tunable chemical environments.^10–12^ However, despite these advantages, the overall uranium recovery remains limited by sluggish adsorption kinetics and inefficient photochemical regeneration, primarily due to insufficient charge separation and restricted transport pathways in conventional COFs.^13,14^ Recent advances in COFs-based uranium extraction materials have mainly focused on tuning local polarization generated through organic linker modification for charge separation and utilization efficiency,^15,16^ enhancing planar conjugation for improved π-electron delocalization and better semiconductor properties,^17,18^ and regulating interlayer stacking to facilitate charge transport.^19,20^ However, these general strategies mainly optimize coordination, light-driven reduction, or charge migration, while the fundamental kinetic mismatch among uranyl adsorption, electron transport, and photocatalytic regeneration, which ultimately governs the overall efficiency of seawater uranium extraction, has not been explicitly addressed (Fig. 1a). Therefore, to achieve high uranium extraction performance in a photoactive COFs-AO system, it is necessary not only to combine adsorption and photoreduction functions, but also to balance the interaction between uranyl adsorption, photochemical reduction, and active site regeneration.

**Fig. 1:**
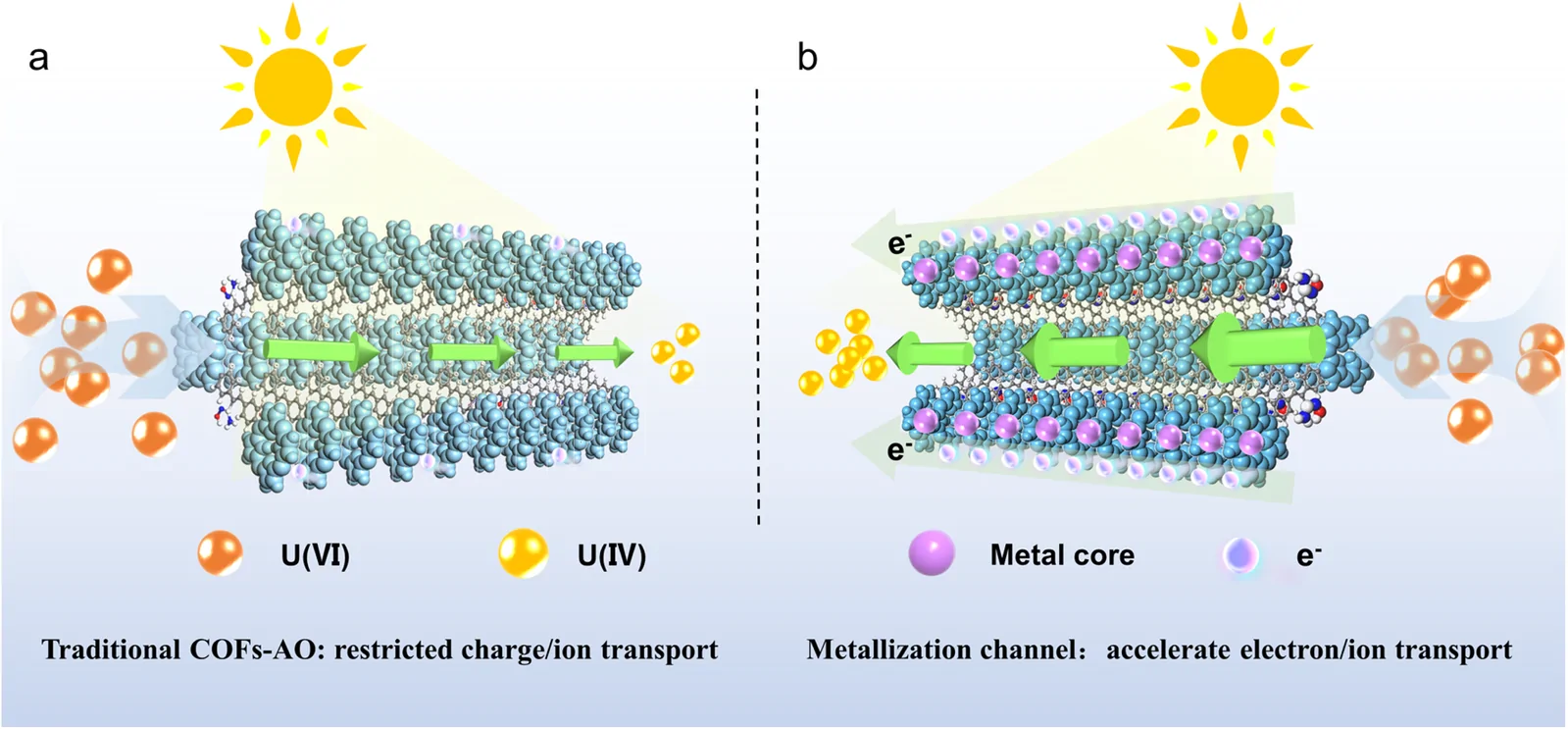
Schematic illustration of (a) the traditional COFs-AO system and (b) the M–Por-COFs-AO with metallized channels.

To address the challenges, herein, we employ porphyrin-incorporated COFs-AO as a robust adsorption–photocatalysis platform. In this system, porphyrin units exhibit extended π-conjugation, rigid planar geometry and well-defined coordination environment, and serve as photoactive centers to provide an ideal molecular platform for tuning metal-dependent π–d orbital hybridization. By systematically tuning the central metal in porphyrin units, the π–d orbital overlap within the porphyrin core was modulated to create more favorable out-of-plane electronic pathways. This metal-dependent orbital hybridization would provide a rational way to promote vertical charge transport and improve the coupling between uranyl adsorption and photochemical reduction. The resulting two-dimensional metallized porphyrin COFs (M–Por-COFs-AO, M = Co, Ni, Cu) are structurally consistent with an AA-stacked layered architecture featuring vertically aligned metallized channels (Fig. 1b). Among the series, Ni–Por-COF-AO exhibits the optimal electronic structure and ion transport dynamics, achieving a uranium extraction capacity of 21.2 mg g^−1^ from natural seawater within seven days under visible-light irradiation in a continuous-flow photochemical system, without the need for sacrificial agents. Moreover, the framework demonstrates excellent U/V selectivity (62.7) and superior cycling stability. Mechanistic analyses and density functional theory (DFT) calculations reveal that the hybridization between Ni e–g orbitals and the porphyrin π-system generates delocalized conductive pathways, promoting synchronized charge and ion transport.

This work establishes a general orbital-hybridized channel engineering paradigm for two-dimensional COFs, demonstrating how metal-centered π–d hybridization can be leveraged to construct vertically aligned conduction pathways that synchronize ion diffusion and electron transport. Beyond materials design, we further introduce a device-level advance by integrating these COFs into a continuous-flow photochemical system, enabling sustained uranyl capture and photoreduction under realistic seawater conditions. Together, these developments provide a structurally coherent and functionally unified strategy that overcomes the long-standing kinetic imbalance between adsorption and photochemical regeneration. More broadly, this approach offers a transferable framework for designing next-generation COFs-based photocatalysts and may be extended to a wide range of ion-electron coupled processes, including redox separations, electrocatalysis, and photocharged adsorption systems.

## 2 Results and discussion

### 2.1 Material design, synthesis, and characterization

To construct two-dimensional COFs with highly ordered channels and enhanced charge/ion transport, a metallized porphyrin-based framework was rationally designed. Transition-metal ions (M = Co, Ni, Cu) were introduced into the porphyrin centers prior to polymerization, aiming to stabilize the AA stacking configuration through metal–ligand coordination and thus reduce the interlayer distance. This structural strategy not only promotes long-range π–π conjugation but also establishes potential conductive pathways for electrons and ions within the COFs matrix. The synthesis of M–Por-COFs-AO followed a three-step procedure (Scheme S1 and Fig. 2a), including (i) coordination of metal ions with 5,10,15,20-tetrakis(4-formylphenyl)porphyrin (Por–CHO) to form M–Por–CHO precursors; (ii) a Knoevenagel condensation between M–Por–CHO and 1,4-phenylenediacetonitrile (PDAN) to afford crystalline M–Por-COFs; and (iii) amidoximation of the cyano groups to yield the final M–Por-COFs-AO. The relevant NMR spectra are provided in the SI (Fig. S1–S8).

**Fig. 2:**
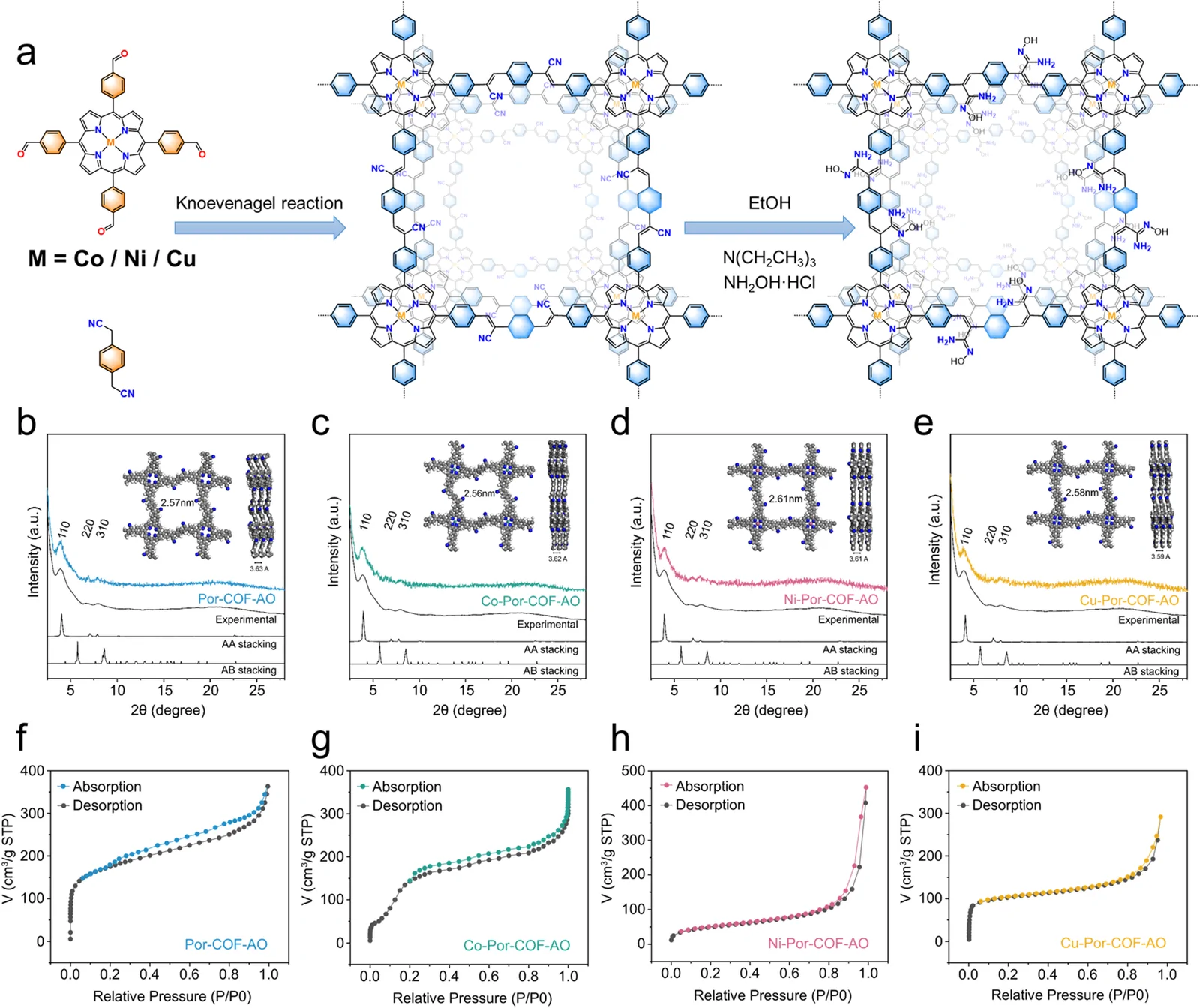
(a) Synthetic route of M–Por-COFs-AO. Experimental PXRD patterns together with simulated PXRD patterns based on AA- and AB-stacking models of Por-COFs-AO (b), Co–Por-COF-AO (c), Ni–Por-COF-AO (d), and Cu–Por-COF-AO (e), suggesting better consistency with the AA-stacking model. N_2_ adsorption–desorption isotherms of Por-COFs-AO (f), Co–Por-COF-AO (g), Ni–Por-COF-AO (h), and Cu–Por-COF-AO (i).

Powder X-ray diffraction (PXRD) patterns confirmed the formation of crystalline COFs (Fig. 2b–e). To further evaluate the stacking mode, the experimental PXRD profiles were compared with simulated patterns based on both AA- and AB-stacking models. The experimental data shows better agreement with the AA-stacking simulations, suggesting that these frameworks are more consistent with an AA-stacked arrangement. Compared with the pristine Por-COF-AO, the metallized analogues exhibit reduced interlayer spacing, consistent with tighter interlayer packing after metal incorporation. Some peaks in PXRD profiles are relatively broad and weak, which may be associated with limited crystallite size and partial structural disorder introduced during post-functionalization and metallization. Therefore, the PXRD results are more appropriately interpreted as being consistent with an AA-stacked model, rather than as definitive proof of a perfectly ordered long-range structure. In addition, the PXRD pattern of the unmodified Por-COF in Fig. S9 shows that the main diffraction features are found to be generally preserved after amidoximation, suggesting that the overall crystalline framework remains largely intact during the post-functionalization process.

Fourier transform infrared (FT-IR) spectra of M–Por-COFs-AO exhibites characteristic bands of amidoxime groups (–C=NOH at 1650 cm^−1^ and –N–O at 930 cm^−1^), confirming successful post-synthetic functionalization (Fig. S10). Solid-state ^13^C NMR test further validates the chemical integrity of the frameworks (Fig. S11). Scanning electron microscopy (SEM) images reveal the uniform, bulk-like particles (Fig. S12), while the high-resolution transmission electron microscope (HRTEM) images clearly show the lattice fringes of M–Por-COF-AO (Fig. S13). The specific surface areas of Por-COF-AO, Co–Por-COF-AO, Ni–Por-COF-AO, and Cu–Por-COF-AO were determined to be 751.3, 612.3, 520.5, and 560.7 m^2^ g^−1^, respectively (Fig. 2f–i). The pore size distributions of these COFs are centered at approximately 1.25 nm, in good agreement with the theoretically predicted pore size, confirming the preservation of the intrinsic porous framework after metallization (Fig. S14). Collectively, these results support the conclusion that the metallized channel engineering strategy yields layered COFs architectures that are structurally consistent with an AA-stacking model while integrating electronic functionality into the framework. The resulting M–Por-COFs-AO materials provide a suitable structural platform for exploring the synergistic coupling of uranium adsorption and photocatalytic reduction.

### 2.2 Electrochemical and photoelectrochemical properties

To elucidate the impact of metallized channel engineering on the optoelectronic properties of COFs, the optical absorption, electronic structure, and charge transport behaviors of the M–Por-COFs-AO series were systematically investigated. The UV-vis diffuse reflectance spectra (Fig. 3a) reveales strong absorption across the visible-light region (400–800 nm) for all M–Por-COFs-AO samples, arising from the π–π* transitions of porphyrin macrocycles and metal–ligand charge transfer transitions. Upon metal incorporation, the absorption intensity notably increases and exhibits slight red-shifts relative to the pristine Por-COF-AO, suggesting enhanced light-harvesting and modified electronic states. Based on Kubelka–Munk plots, the optical band gaps of Co–, Ni–, and Cu–Por-COF-AO were determined to be 1.86, 1.59, and 1.87 eV, respectively. This suggests that Ni incorporation leads to a narrower band gap, which in turn facilitates visible-light-driven charge excitation (Fig. S15).

**Fig. 3:**
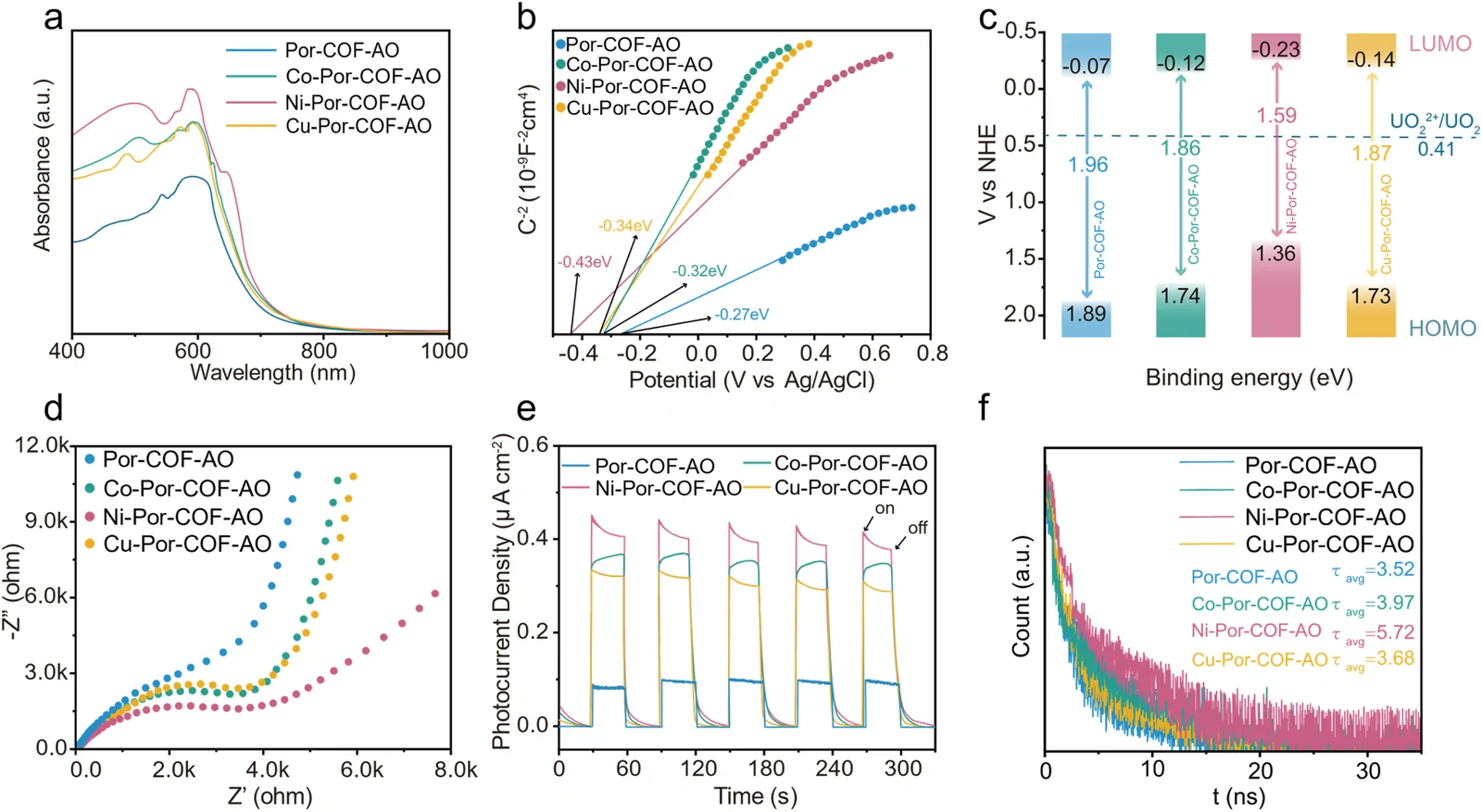
(a) UV-vis DRS spectra of M–Por-COFs-AO. (b) Mott–Schottky plots of M–Por-COFs-AO. (c) Energy band alignment diagrams of M–Por-COFs-AO. The flat-band potentials *vs.* Ag/AgCl were converted to the NHE scale and used to approximate the CB positions. (d) EIS Nyquist plots of M–Por-COFs-AO. (e) Transient photocurrent responses in 0.5 M Na_2_SO_4_ under visible light irradiation. (f) Time-resolved photoluminescence spectra of M–Por-COFs-AO.

Mott–Schottky analyses confirms that all M–Por-COFs-AO samples behave as n-type semiconductors with positive slopes in their 1/*C*^2^–*V* curves (Fig. 3b). The flat-band potentials were measured to be −0.32, −0.43, and −0.34 V *vs.* Ag/AgCl for Co–, Ni–, and Cu–Por-COF-AO, respectively, corresponding to −0.12, −0.23, and −0.14 V *vs.* NHE after conversion (Fig. 3c). For these n-type materials, the flat-band potentials were used to approximate the conduction-band (CB) positions. Combined with the optical band gaps, the valence-band (VB) positions are estimated to be 1.75, 1.36, and 1.73 V *vs.* NHE, respectively. Importantly, the CB edge of Ni–Por-COF-AO lies above the redox potential of U(vi)/U(iv) (0.41 V *vs.* NHE), rendering it thermodynamically favorable for photoreduction of uranyl ions.

Electrochemical impedance spectroscopy (EIS, Fig. 3d) reveals a pronounced reduction in charge-transfer resistance for Ni–Por-COF-AO, evidenced by the smallest semicircular radius among all samples. This suggests that the well-aligned, metal-stabilized channels effectively promote interfacial charge transport. Consistently, transient photocurrent responses (Fig. 3e) demonstrates that Ni–Por-COF-AO generates the highest photocurrent density, approximately 4.3 times greater than that of Por-COF-AO, highlighting its superior light-to-charge conversion efficiency. Time-resolved photoluminescence (TRPL) spectroscopy (Fig. 3f) further probes carrier recombination dynamics. Ni–Por-COF-AO exhibits the longest average carrier lifetime (5.72 ns) compared to Co-analogue (3.97 ns) and Cu-analogue (3.68 ns), demonstrating the most efficient suppression of electron–hole recombination. The extended lifetime is attributed to delocalized π–d orbital hybridization within the metallized channels, which facilitates rapid charge separation and transport. These results collectively demonstrate that metallized channel engineering not only tunes the band structure but also enhances carrier mobility and charge-separation efficiency.

### 2.3 Uranium adsorption and photocatalytic extraction performance

To evaluate the uranium capture ability of the metallized COFs, batch adsorption experiments were conducted under simulated seawater conditions both in the dark and under visible-light irradiation. As shown in Fig. 4a, all M–Por-COF-AO materials exhibit significantly higher uranium uptake capacities than the pristine Por-COF-AO, even in the dark, demonstrating that metallized channel engineering intrinsically enhances uranyl affinity and adsorption kinetics. Among the four materials, Ni–Por-COF-AO shows the most pronounced uranium uptake enhancement even under dark conditions, indicating that Ni incorporation not only benefits the photoinduced extraction pathway under illumination but also intrinsically strengthens uranyl adsorption in the absence of light. This dark-condition improvement may be attributed to the stronger uranyl-binding affinity and the more favorable local channel environment introduced by the Ni-metallized porphyrinic framework. Upon visible-light illumination, the extraction performance further improved due to photocatalytic coupling. Notably, Ni–Por-COF-AO achieves the highest uranium extraction capacity of 516.9 mg g^−1^, which represents a 44.5% increase compared with the dark condition and nearly three times that of Por-COF-AO (182.7 mg g^−1^). The equilibrium isotherms (Fig. 4b) show that the maximum adsorption capacity of Ni–Por-COF-AO at 298 K is 759.1 mg g^−1^. This pronounced light-responsive enhancement, particularly the significant capacity increase under illumination for Ni–Por-COF-AO, indicates that the metallized channels enable a more efficient coupling between the adsorption and photocatalytic reduction processes. The enhanced electron transport kinetics appear to be well-matched with the mass transport (ion diffusion and adsorption), leading to a more balanced and synergistic cycle. To further clarify the evidence chain for the proposed electron-mass transport interplay, the charge-transport descriptors and uranium-capture performance descriptors of the four samples were summarized in a normalized, side-by-side manner (Table S1). Specifically, Ni–Por-COF-AO exhibits the smallest charge-transfer resistance (*R*_ct_), the highest transient photocurrent response, and the longest TRPL average lifetime among the four materials, indicating the most favorable charge-transfer and charge-separation behavior. Meanwhile, Ni–Por-COF-AO also shows the highest uranium uptake under both light and dark conditions, the highest equilibrium adsorption capacity, and the highest distribution coefficient. These results suggest a positive correspondence between improved charge-transfer/charge-separation behavior and enhanced uranium-capture performance across the sample series. Accordingly, the present results are more appropriately interpreted as being consistent with a more coordinated interplay between charge transport and uranium capture/conversion, rather than constituting direct proof of a rigorously quantified transport coupling.

**Fig. 4:**
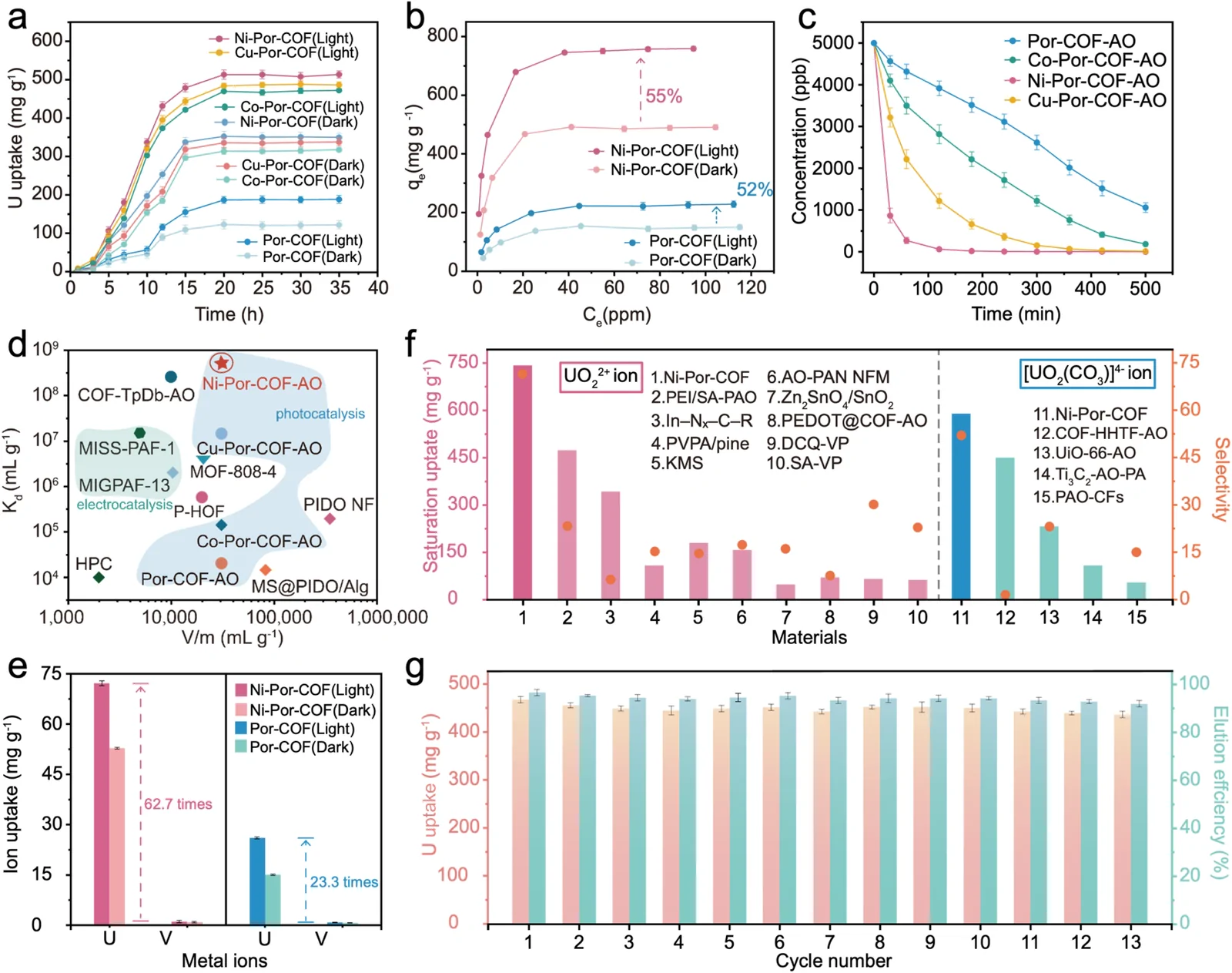
(a) Uranium extraction capacities of Por-COF-AO, Co–Por-COF-AO, Ni–Por-COF-AO, and Cu–Por-COF-AO in uranium-spiked seawater under dark and light (conditions: 150 mL solution, 20 ppm U-spiked seawater, 5 mg COFs). (b) The equilibrium adsorption isotherms of uranium adsorption by Por-COF-AO and Ni–Por-COF-AO in seawater with different initial concentrations of uranyl added (shown in the uranium concentration range from 5 to ∼120 ppm). (c) *K*_d_ values of four COFs in 5 ppm U(vi) solution. (d) *K*_d_*vs. V*/*m* ratios of benchmark adsorbents (Table S2). (e) Selectivity (*S*_U/V_) of Por-COF-AO and Ni–Por-COF-AO in ASTM D1141-98 artificial seawater at pH = 8 under visible-light irradiation, with U(vi), V, Cu, Fe, Zn, and Co each fixed at 5 ppm (V was introduced as NaVO_3_). (f) Comparison of ion selectivity and saturation capacity between Ni–Por-COF-AO and reported adsorbents (Table S3). (g) Uranium adsorption capacity and desorption efficiency of Ni–Por-COF-AO.

The distribution coefficient (*K*_d_) of Ni–Por-COF-AO reached 1.2 × 10^9^ mL g^−1^, outperforming benchmark adsorbents such as MOF-808-4 (6.5 × 10^6^ mL g^−1^), COF-TpDb–AO (3.6 × 10^8^ mL g^−1^), and MIGPAF-13 (2.0 × 10^6^ mL g^−1^) (Fig. 4c, d and Table S2). Selectivity tests conducted in uranium–vanadium mixed seawater solutions (pH = 8) revealed a remarkable U/V selectivity ratio (*S*_U/V_) of 62.7, substantially higher than that of Por-COF-AO (23.3) (Fig. 4e). The selectivity test was carried out in ASTM D1141-98 artificial seawater under visible-light irradiation at pH 8, with U(vi), V, Cu, Fe, Zn, and Co simultaneously present at 5 ppm; V was introduced as NaVO_3_. Under the tested conditions, vanadium is expected to exist predominantly as vanadate-like anionic species (mainly H_2_VO_4_^−^/HVO_4_^2−^), whose charge character and coordination behavior differ substantially from those of uranyl species. Accordingly, the high U/V selectivity of Ni–Por-COF-AO is more appropriately attributed to selective uranyl–amidoxime coordination together with channel-confined discrimination, rather than simple sieving alone. Among the tested factors, bicarbonate shows the most noticeable influence, whereas chloride and sulfate induce comparatively milder changes; increasing ionic strength also leads to some attenuation in uptake/selectivity. These observations indicate that the U/V selectivity is governed by a combination of hydration behavior, charge character, coordination geometry, and competitive binding affinity, rather than simple pore-size exclusion alone. Here, *S*_U/V_ was calculated from the ratio of the adsorption capacities of U and V under the same conditions. In addition, the effects of HCO_3_^−^, SO_4_^2−^, Cl^−^, and ionic strength on the U/V selectivity of Ni–Por-COF-AO under visible-light irradiation were further evaluated and are presented in Fig. S21.

Even in the presence of other competing cations (Co^2+^, Zn^2+^, Cu^2+^, Fe^3+^, VO_4_^2−^), Ni–Por-COF-AO retained superior uranium selectivity (Fig. S16), underscoring the intrinsic advantage of the metallized channels in electrostatic discrimination and uranyl coordination. Compared to other high-efficiency adsorbents or technologies reported in the literature, Ni–Por-COFs-AO exhibits remarkably favorable extraction performance, demonstrating great promise for practical applications (Fig. 4f and Table S3). Cyclic adsorption–desorption tests demonstrates excellent structural and functional stability. After 13 consecutive extraction cycles, Ni–Por-COF-AO retains over 92% of its initial capacity without noticeable morphology degradation (Fig. 4g and S18). To further verify the structural and chemical stability of Ni–Por-COF-AO after repeated cycling, post-cycle PXRD, FT-IR, and N_2_ sorption analyses were performed (Fig. S17). The cycled sample retains the characteristic low-angle PXRD reflections, although with slightly broadened and weakened intensity, indicating that the crystalline COFs remains largely preserved after repeated adsorption–desorption cycles. The post-cycle FT-IR spectrum remains largely consistent with that of the fresh sample, and the characteristic amidoxime bands at 1650 cm^−1^ (–C=NOH) and 930 cm^−1^ (–N–O) are still retained, suggesting that the framework linkages and AO functional groups remain chemically intact. In addition, post-cycle N_2_ sorption analysis shows that the cycled sample still exhibits a BET surface area of 416.8 m^2^ g^−1^, corresponding to about 80% of that of the pure Ni–Por-COF-AO (520.5 m^2^ g^−1^), further indicating that appreciable porosity is retained after repeated cycling. Moreover, ICP-OES analysis of the post-cycle supernatant shows that the concentration of leached Ni was below the detection limit (LOD = 1.5 µg L^−1^), further confirming the robustness of the metallized framework.

### 2.4 Continuous-flow adsorption–photocatalytic uranium extraction from natural seawater

To explore the practical feasibility and address the challenge of catalyst recovery, Ni–Por-COF-AO was immobilized within a custom-designed flow photochemical reactor (Fig. 5a and b) with serpentine microchannels (8 × 8 cm^2^ plate area). Prior to the seawater extraction tests, the effect of flow rate on uranium extraction was systematically evaluated in uranium-spiked seawater (20 ppm U) at 1, 2, and 4 mL min^−1^ (Fig. 5c). The extraction performance decreased with the increase of flow rate, indicating that longer residence time favors mass transport and photoinduced reduction. Under visible-light irradiation, natural seawater (3.3 ppb U) continuously circulated through the reactor for 7 days. The system achieves a uranium extraction capacity of 21.2 mg g^−1^ under visible-light irradiation, which is approximately three times higher than that under dark conditions (6.9 mg g^−1^), highlighting the significant contribution of photocatalysis (Fig. 5d). X-ray photoelectron spectroscopy (XPS) analysis confirms that U(vi) was photoreduced to U(iv) on the Ni–Por-COF-AO surface (Fig. 5e), evidencing efficient photoelectron participation in uranium reduction. This performance surpasses that of most previously reported adsorbents under comparable real-seawater conditions (Fig. 5f and Table S4). The seawater was pretreated by coarse quartz-sand filtration to remove suspended particulates and most plankton, followed by powdered activated carbon treatment and 0.45 µm membrane filtration. During operation, the seawater exhibites a pH of 8.1–8.2 and a salinity of 32–34, and the experiment was carried out at room temperature (25 ± 2 °C). Outlet samples were collected every 24 h during the 7 day continuous-flow test. The reactor was irradiated from the top by a 300 W Xe lamp with a light intensity of 1 kW m^−2^, positioned approximately 50 cm above the reactor. Illumination was applied from one side only, and the incident light passed through the outer quartz plate to the Ni–Por-COF-AO-coated microchannel region, covering nearly the entire 8 × 8 cm^2^ reactor plate.

**Fig. 5:**
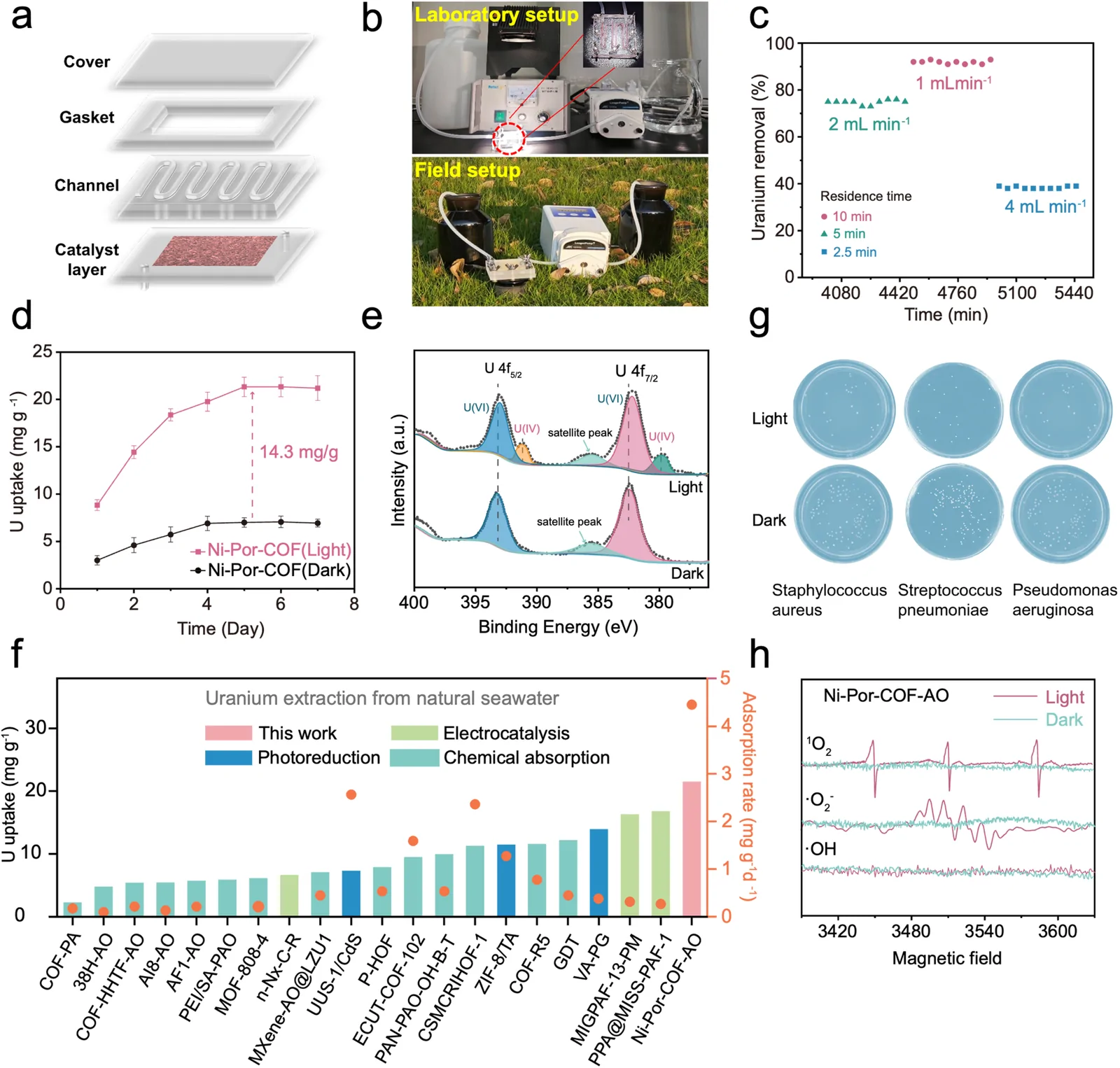
(a) Structure diagram of the panel reactor. (b) Schematic of flow photocatalytic device: dual-layer quartz plates with Ni–Por-COF-AO-coated microchannels (operation: seawater flow controlled by peristaltic pump). (c) Uranium extraction (20 ppm U) under varying flow rates. (d) Uranium extraction capacity from natural seawater under dark and light; (reaction condition: 50 L natural seawater (10 mL min^−1^), 5 mg Ni–Por-COF-AO). (e) U 4f XPS spectra of uranium-loaded Ni–Por-COF-AO under dark and illuminated conditions. (f) Comparison of uranium uptake capacities and adsorption rates for reported adsorbents in natural seawater (SI Table S4). (g) Antibacterial spectrum (inhibition rates against *E. coli* and *S. aureus*). (h) EPR spectra under visible light irradiation of Ni–Por-COF-AO.

Biofouling is a critical obstacle for long-term uranium extraction in marine environments. The Ni–Por-COF-AO exhibits notable intrinsic antibiofouling capability, attributed to its photoactive and oxidative surface chemical properties. Under dark conditions, moderate inhibitions of *Staphylococcus aureus*, *Streptococcus pneumoniae*, and *Pseudomonas aeruginosa* were observed (21–48%). Under visible-light illumination, inhibition rates dramatically increased to 84–90%, demonstrating broad-spectrum antibiofouling activity (Fig. 5g). Electron paramagnetic resonance (EPR) spectroscopy identifies the generation of reactive oxygen species (^1^O_2_, ˙O_2_^−^ and ˙OH) under illumination (Fig. 5h), which can effectively suppress microbial attachment while contributing to uranium photoreduction.

Taken together, these results establish that Ni–Por-COF-AO integrates structural, photochemical, and biological functionalities. The metallized channel engineering not only accelerates ion–electron transport for efficient uranium recovery but also confers long-term operational stability through intrinsic antibiofouling activity—key advantages for realistic seawater extraction applications.

### 2.5 Adsorption–photocatalytic mechanism

To elucidate the microscopic origin of the enhanced uranium extraction performance of M–Por-COF-AO, we combined DFT calculations with XPS, EPR, and radical trapping experiments. DFT calculations elucidate how metal–ligand interactions modulate the electronic structure, while XPS probes the evolution of chemical states at the active sites. EPR and radical trapping experiments further identify the key reactive species and their transformation pathways. Collectively, this multi-scale investigation establishes a coherent mechanistic framework that bridges electronic structure and macroscopic performance, thereby revealing the intrinsic structure–activity relationships governing the system.

High-resolution XPS spectra provided direct evidence of uranyl coordination with amidoxime sites. After U(vi) adsorption, the N 1s peak of Ni–Por-COF-AO shifted from 400.3 eV (–C=NOH) to 401.2 eV, accompanied by the emergence of a shoulder peak at 401.1 eV, attributable to the N–U coordination bond (Fig. S20). Correspondingly, the O 1s component at 531.8 eV increased in intensity, consistent with U–O–C/N interactions, confirming strong chelation between amidoxime oxygen/nitrogen donors and uranyl ions. DFT calculations revealed the binding energies of uranyl ions at amidoxime sites to be −17.2 eV (Por-COF-AO), −19.4 eV (Co–Por-COF-AO), −21.1 eV (Ni–Por-COF-AO), and −20.2 eV (Cu–Por-COF-AO) (Fig. 6a). The substantially stronger binding in Ni–Por-COF-AO verifies that metallic channel incorporation significantly reinforces uranyl–amidoxime interactions, in agreement with the experimentally observed higher affinity (*K*_d_ = 1.2 × 10^9^ mL g^−1^). This stronger uranyl binding also explains the superior uranium uptake of Ni–Por-COF-AO under dark conditions, where intrinsic adsorption affinity rather than photocatalytic reduction dominates the extraction behavior.

**Fig. 6:**
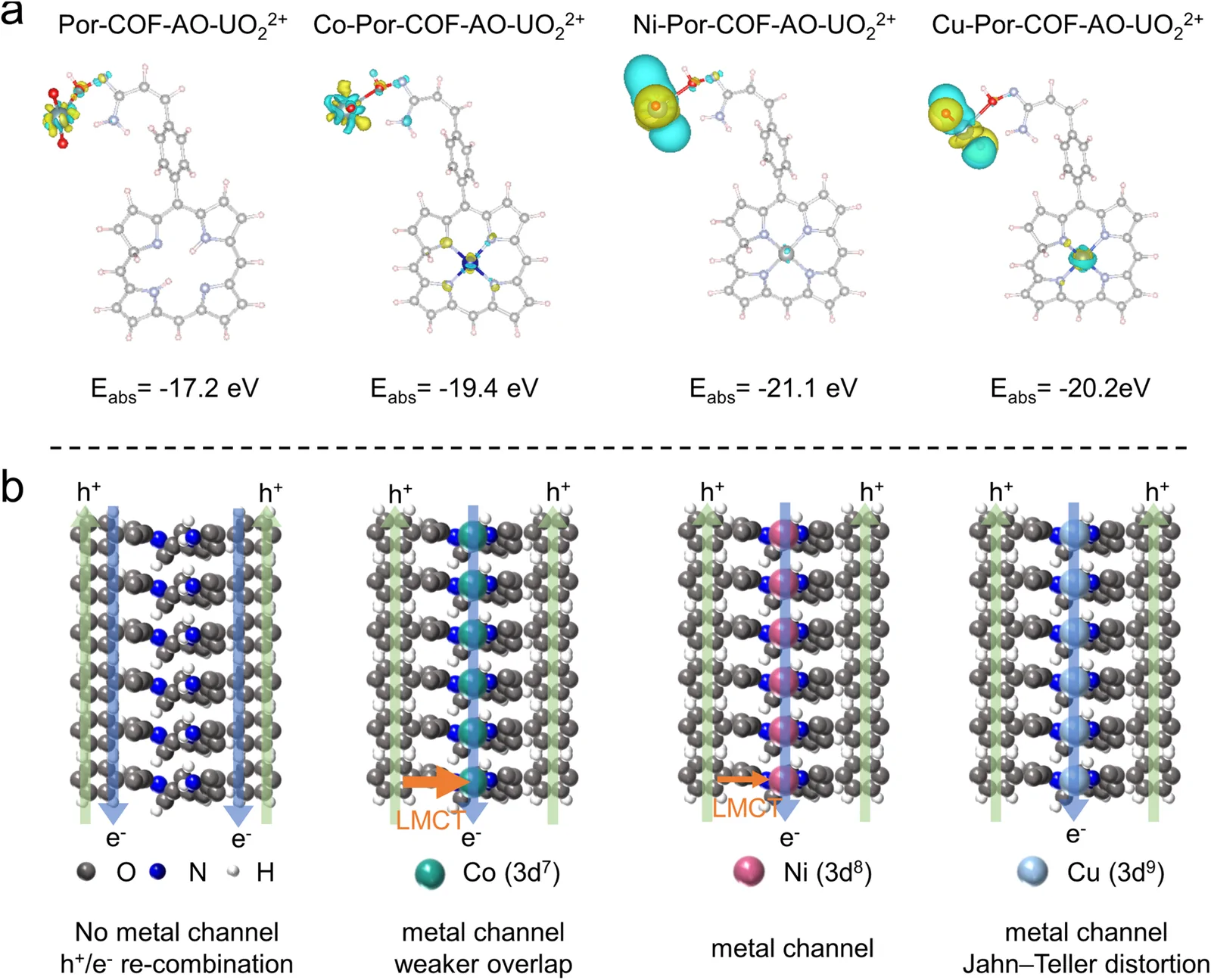
DFT calculations for the adsorption binding energy and the LMCT mechanism. (a) Charge-density difference maps and adsorption energies after M–Por-COF-AO adsorbs uranyl ions, and (b) proposed charge/hole transport pathways in M–Por-COF-AO.

Charge density difference maps further elucidated the electronic interactions between the metalloporphyrin centers and the conjugated COF network. Considering the AA-stacked architecture of M–Por-COF-AO, both macrocycle–macrocycle and metal–metal channels play vital roles in governing charge-carrier separation and migration dynamics. Upon visible-light excitation of the porphyrin π system, photoexcited electrons are proposed to migrate through the metal-centered π–d hybridized channel, as evidenced by the charge density difference maps (Fig. 6a and b). For the non-metallized Por-COF-AO, in the absence of metal centers, both electrons and holes are confined to macrocycle–macrocycle transport, leading to rapid electron–hole recombination and a shorter exciton lifetime. In contrast, in Co- and Ni-metallized systems, the introduction of transition metals activates a ligand-to-metal charge transfer (LMCT) process that modulates the balance between electron and hole migration. Specifically, in Co–Por-COF-AO (3d^7^), LMCT is dominant, suppressing hole migration through the macrocyclic pathway and thus resulting in inferior photocatalytic activity. As the d-electron count increases in Ni–Por-COF-AO (3d^8^), the LMCT effect is partially mitigated, allowing improved hole transport and more balanced charge separation. Conversely, in Cu–Por-COF-AO (3d^9^), strong Jahn–Teller distortion disrupts the metal–metal conduction pathway, leading to less efficient charge migration. Therefore, Ni^2+^ achieves an optimal electronic configuration, where moderate LMCT coupling and effective π–d orbital overlap produce delocalized conduction pathways, minimizing electron–hole recombination and enabling superior photocatalytic uranium reduction performance.

High-resolution XPS analysis was performed to investigate the chemical states of the metal sites in M–Por-COF-AO before and after photocatalysis. Taking Ni–Por-COF-AO as a representative example (similar trends were observed for Co and Cu), the Ni 2p_3/2_ peak at 855 eV, accompanied by characteristic satellite peaks, remain unchanged after the reaction (Fig. S19). This indicates that the Ni center maintains a stable +2 oxidation state, without undergoing irreversible redox transformation. Furthermore, no detectable Ni leaching was observed in the post-reaction solution by ICP-OES, suggesting that the Ni center does not act as a direct redox-active site but rather serves as an electron transfer mediator.^21^ Under photoexcitation, electrons are transferred from the porphyrin ligand to the Ni 3d orbitals, forming a transient high electron density state. Due to the electronic stability of Ni(ii), these electrons are rapidly delocalized and subsequently transferred to adsorbed species.

In the O 1s spectra, the increased intensity at 531.8 eV is attributed to U–O–C/N interactions, confirming strong coordination between uranyl ions and the amidoxime functional groups. Analysis of the U 4f spectra of the reacted material reveals the emergence of U(iv) species alongside U(vi), indicating a stepwise reduction pathway *via* a U(v) intermediate.^22^ Radical quenching experiments further elucidate the active species involved. Isopropanol (˙OH scavenger) and *p*-benzoquinone (˙O_2_^−^ scavenger) significantly inhibited the reaction, while the addition of AgNO_3_ (electron scavenger) nearly suppressed the reduction entirely, highlighting the critical role of photogenerated electrons.^23^ EPR analysis of Ni–Por-COF-AO reveals that ˙OH, ˙O_2_^−^, and ^1^O_2_ were generated exclusively under light irradiation, whereas negligible signals were observed in the dark, confirming the photoinduced nature of these reactive species. In addition, H_2_O_2_ was also detected in the reaction products. The introduction of methanol as a hole scavenger enhanced the reaction efficiency, indicating that suppressing charge recombination is essential for improving photocatalytic performance.^24^

Collectively, these results suggest that photogenerated electrons are the primary driving force for U(vi) reduction, while ˙O_2_^−^ may act as a secondary reductive intermediate. In contrast, ˙OH radicals are likely associated with parallel oxidative pathways. Under aerobic conditions, electrons and holes react with dissolved oxygen and water, respectively, generating reactive species that contribute to the formation of mixed-valence uranium(iv&vi) oxide precipitates.

Based on the above experimental and theoretical results, a synergistic adsorption photocatalysis mechanism is proposed as follows.

1 M–Por-COF-AO + ℏ*ν* → e^−^ + h^+^

2 O_2_ + e^−^ → ˙O_2_^−^

3 UO_2_^2+^ + e^−^ → UO_2_^+^

4 UO_2_^+^ + e^−^ → UO_2_ (s)

5 2UO_2_^+^ → UO_2_^2+^ + UO_2_ (s)

6 UO_2_^+^ + ˙O_2_^−^ + 2H_2_O → (UO_2_)O_2_·2H_2_O (s)

7 H_2_O + h^+^ → ˙OH + H^+^

8 2˙OH → H_2_O_2_

9 ˙O_2_^−^ + e^−^ + 2H^+^ → H_2_O_2_

10 UO_2_^2+^ + H_2_O_2_ + 2H_2_O → (UO_2_)O_2_·2H_2_O (s) + 2H^+^

Upon coordination, uranyl ions (UO_2_^2+^) are selectively captured by amidoxime groups anchored within the metallized channels. Under visible-light irradiation, the porphyrin π system is excited to generate electron–hole pairs. The photogenerated electrons subsequently undergo directional migration through a conductive pathway formed by π–d orbital hybridization among the porphyrin ligands, Ni 3d orbitals, and the adsorbed U(vi) species. Electrons arriving at the adsorption sites drive the stepwise reduction of U(vi) to U(v) and further to U(iv), ultimately precipitating as insoluble uranium species (*e.g.*, UO_2_). In parallel, a fraction of electrons is captured by dissolved oxygen to generate superoxide radicals (˙O_2_^−^), which further contribute to the reduction process. Meanwhile, photogenerated holes migrate to the surface and are consumed *via* oxidation of water or amidoxime groups, accompanied by proton release and regeneration of active sites, thereby completing the catalytic cycle.^25^ Hydroxyl radicals (˙OH) generated from the oxidation of water by photogenerated holes can recombine to form hydrogen peroxide (H_2_O_2_), which may further participate in uranium transformation processes. However, in practical water systems, holes are also likely to be consumed by the oxidation of coexisting organic species, thereby suppressing the pathways described in eqn (7)–(10). This mechanism highlights the critical role of the π–d hybridized conductive pathway, which not only facilitates efficient electron transport but also spatially integrates electron transfer with uranyl adsorption, providing a molecular-level basis for the enhanced selectivity and efficiency of uranium extraction. Upon visible-light excitation of the porphyrin π system, photoexcited electrons are proposed to migrate through the metal-centered π–d hybridized channel, as evidenced by the charge density difference maps (Fig. 6b).

Based on the combined experimental and theoretical insights, a synergistic adsorption–photocatalytic mechanism is proposed based on following processes. (1) Uranyl adsorption: amidoxime groups anchored within the metallized channels capture UO_2_^2+^ ions through bidentate N/O coordination, enhanced by the localized electrostatic field of the metal center. (2) Photoinduced charge migration: under visible-light irradiation, LMCT and metal-to-ligand charge transfer processes generate delocalized electrons within the metallized channels. (3) Selective reduction: these photoelectrons are directionally transported along the transition metal–porphyrin conduction pathway to the adsorbed uranyl species, reducing U(vi) to U(iv), while photogenerated holes simultaneously oxidize residual surface groups to regenerate the active amidoxime sites. This self-consistent cycle synchronizes uranyl adsorption and photocatalytic reduction, thereby achieving highly efficient and continuous uranium recovery from seawater. The metallized channels thus serve as dual-function conduits for ion sieving and directional electron transfer—establishing a unifying mechanistic foundation for COF-based photoactive adsorbents.

### 2.6 Techno-economic and life-cycle assessment of uranium extraction

While high extraction capacity is essential, large-scale deployment of seawater uranium recovery also requires a comprehensive understanding of economic feasibility and environmental sustainability. To this end, we conducted both techno-economic analysis (TEA) and cradle-to-gate life-cycle assessment (LCA) of Ni–Por-COF-AO, integrating laboratory performance data with industrial-scale assumptions. Given the assumption-sensitive nature of such analyses, we explicitly define key inputs, calculation logic, and system boundaries to improve transparency and reproducibility.

The cost estimation is based on a projected industrial-scale solvothermal synthesis, with the material cost of COFs assumed to range between US$2000–5000 per kg, consistent with previously reported benchmarks for high-performance COF materials considering precursor complexity, solvent usage, and post-functionalization; this range is treated as an uncertainty interval rather than a fixed value. Under the maximum adsorption capacity (759.1 mg g^−1^), the theoretical uranium extraction cost is estimated to be US$2600–6600 per kg U, substantially lower than most previously reported COFs-based adsorbents (typically >US$10 000 per kg U due to limited capacity), representing an idealized lower-bound scenario. Under more practical conditions, that is, natural seawater in a flow photochemical system yielding 21.2 mg g^−1^ uranium uptake over 7 days, the single-cycle extraction cost, defined as the ratio of material cost to uranium uptake per cycle, is estimated to be US$94 000–236 000 per kg U, representing a conservative upper bound without material reuse. When recyclability is incorporated, the experimentally demonstrated regeneration stability (∼92% capacity retention after 13 cycles) leads to a cumulative uranium yield of approximately 234 mg g^−1^, thereby reducing the amortized extraction cost to US$8600–21 400 per kg U. This multi-cycle cost explicitly accounts for cumulative uptake, regeneration efficiency, and capacity decay, and indicates that adsorbent lifetime is the dominant factor governing economic viability. Overall, this analysis suggests that the metallized channel engineering approach provides substantial performance improvement with minimal additional cost, leading to a 2–3 fold enhancement in cost efficiency compared with conventional amidoxime-COFs systems (Fig. 7a).

**Fig. 7:**
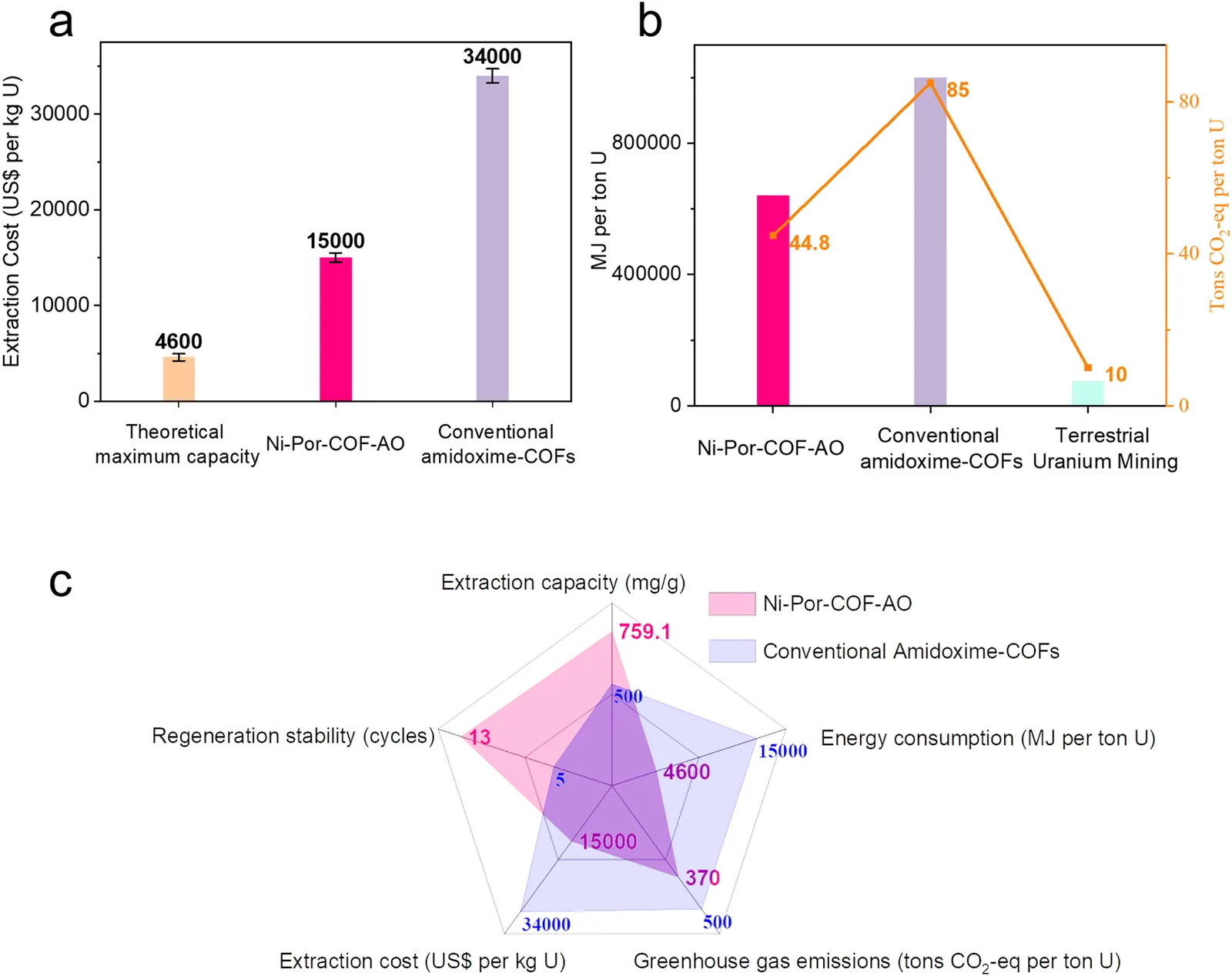
(a) Cost comparison analysis of uranium extraction (TEA). (b) Comparison chart of lifecycle energy and carbon emissions (LCA). (c) Integrated performance radar chart (TEA + LCA consolidation).

To further evaluate environmental sustainability, a cradle-to-gate LCA was performed considering the energy and carbon footprints associated with Ni–Por-COF-AO synthesis and operation. The system boundary includes precursor synthesis (porphyrin and linkers), COF fabrication (solvothermal synthesis and amidoximation), and operational energy input (light irradiation and flow system), while excluding end-of-life disposal and large-scale marine deployment infrastructure; solvent recovery and waste treatment are not explicitly modeled but are partially reflected as implicit energy contributions, which may lead to conservative estimation uncertainty. The solvothermal synthesis of COFs is assumed to consume 120–180 MJ kg^−1^, dominated by solvent heating and purification steps. Based on the cumulative uranium uptake of 234 mg g^−1^ after 13 reuse cycles, the corresponding energy requirement for uranium recovery is calculated to be approximately 6.4 × 10^5^ MJ per ton U. Although this value remains higher than that of terrestrial uranium mining (5–10 × 10^4^ MJ per ton U), it represents a 30–40% reduction compared with conventional amidoxime-COFs lacking channel engineering (typically exceeding 1 × 10^6^ MJ per ton U). Using an average carbon emission factor of 0.07 kg CO_2_-eq per MJ, the associated greenhouse gas emissions are estimated at approximately 44.8 tons CO_2_-eq per ton U, again significantly lower than those of most reported COF-based adsorbents (70–100 tons CO_2_-eq per ton U) (Fig. 7b). Contribution analysis indicates that porphyrin precursor synthesis and amidoximation modification account for more than 70% of the total environmental burden, whereas operational energy plays a comparatively smaller role. These results further suggest that process optimization strategies, including continuous-flow synthesis, solvent recovery, and renewable precursor utilization, could substantially improve the sustainability profile of COF-based uranium adsorbents.

Considering the inherent uncertainties in TEA and LCA, the present results should be interpreted as order-of-magnitude estimates rather than absolute values. Sensitivity analysis indicates that economic outcomes are most strongly influenced by adsorbent lifetime and regeneration efficiency, whereas environmental impacts are dominated by synthesis energy intensity and precursor production. Nevertheless, the relative advantage of the present system remains robust across reasonable parameter ranges. Combining TEA and LCA results, Ni–Por-COF-AO demonstrates a favorable balance between extraction performance, economic cost, and environmental impact (Fig. 7c). The metallized channel engineering strategy not only enhances uranium extraction efficiency but also contributes to reduced carbon intensity and improved resource utilization. Moreover, the flow photochemical system enables scalable operation, continuous uranium capture, and facile adsorbent recovery, representing a critical step toward bridging laboratory-scale studies and practical marine uranium recovery. Overall, these findings underscore that integrating molecular-level design (metallized channels) with system-level process optimization (flow photochemical operation) is essential for advancing seawater uranium extraction toward real-world viability.

## 3 Conclusion

In summary, a metallized channel engineering strategy was developed for constructing two-dimensional porphyrin-based COFs (M–Por-COFs-AO) that effectively synchronizes ion and electron transport. This approach successfully addresses the kinetic mismatch between adsorption and photocatalysis, achieving a more balanced process for uranium extraction. Incorporating transition metals (Ni, Cu, Co) into porphyrin centers stabilize AA stacking and generate conductive channels that synergistically couple uranyl adsorption and photocatalytic reduction. Among them, Ni–Por-COF-AO exhibits exceptional uranium affinity (*K*_d_ = 1.2 × 10^9^ mL g^−1^), high U/V selectivity (62.7), and achieves 21.2 mg g^−1^ uranium extraction from natural seawater in 7 days using a flow photochemical system without sacrificial agents. Mechanistic and DFT analyses reveal that Ni–porphyrin π–d hybridization forms delocalized conduction pathways enabling efficient charge transfer. TEA and LCA suggest improved cost-effectiveness and lower carbon intensity relative to conventional COFs, respectively. This work establishes a general design paradigm based on orbital-hybridized channel engineering that couples molecular-level electronic engineering with system-level process innovation to achieve balanced ion-electron transport. The concept of metallized conductive channels opens new avenues for developing multifunctional COFs capable of synchronized ion–electron transport, offering promising opportunities for sustainable uranium recovery and broader applications in photocatalysis, energy conversion, and environmental remediation.

## Author contributions

Bin Zuo: data curation, writing – original draft; Ruoyu Wang: data curation, writing – original draft; Guoze Yan: data curation, writing – original draft; Jiayu Zuo: writing – review and editing; Likun Pan: writing – review and editing; Dong Jiang: supervision, writing; Yusuke Yamauchi: supervision, writing – review and editing; Xingtao Xu: supervision, writing – review and editing.

## Conflicts of interest

The authors declare no competing financial interest.

## Supplementary Material

SC-017-D6SC00048G-s001

## Data Availability

All the data supporting this study and its findings are available within the article and supplementary information (SI). Additional supporting data for this study are available from the corresponding authors upon request. Supplementary information is available. See DOI: https://doi.org/10.1039/d6sc00048g.
