# Supplementary material for: Metallized porphyrin-based covalent organic frameworks with orbital-hybridized conduction channels for continuous-flow adsorption–photocatalytic uranium extraction from seawater
Source: Chem Sci. 2026 Aug 10;17(38):18178–90. doi: 10.1039/d6sc00048g (PMC13456040; doi:10.1039/d6sc00048g)
Supplement: Supplementary file 1 [file SC-017-D6SC00048G-s001.pdf]

This journal is © The Royal Society of Chemistry 2026

SI. Characterizations

The morphology of the materials was characterized *via* scanning electron microscopy (SEM, VEGA3, TESCAN) and transmission electron microscopy (TEM, JEOL JEM-2100F). Energy-dispersive X-ray spectroscopy (EDS) was conducted using SEM equipped with an EDX detector. Powder X-ray diffraction (XRD) measurements were performed using an analyzer (D8 Advance, Bruker, Germany) with Cu-Kα radiation (λ = 1.5406 Å). The specific surface area and pore-size distribution of the materials were recorded using a Tristar II 3020 (Micromeritics Inc., USA) at 77 K in the P/P_0 range of 0.05–0.2. Samples were degassed at 150 ºC for 4 h under vacuum before N_2 adsorption/desorption measurements. Fourier-transform infrared (FTIR) spectra were recorded on an AVATAR 360 spectrometer (Nicolet, USA) using the KBr disk method in the range of 4000−400 cm^{-1}. X-ray photoelectron spectroscopy (XPS) was performed using an ESCALAB 250 spectrometer (Thermo Fisher Scientific) with an Al Kα X-ray source. Solid-state ^{13}C NMR spectra were acquired on a Bruker AVANCE III 400 WB spectrometer operating at a MAS rate of 5 KHz. UV−VIS absorption spectra were measured using a spectrophotometer (Cary500, VARIAN, America). Metal ions concentrations were determined using an iCAP Q inductively coupled plasma mass spectrometry (ICP-MS, Thermo Fisher Scientific, USA). Electron spin resonance (ESR) was used to investigate the reactive species, which was recorded using a Bruker A300 spectrometer. The time-resolved transient PL decay (TR-PL) spectra were carried on an Edinburgh Instruments FLS 920 fluorescence spectrophotometer.

SII. Materials and Methods

2.1 Materials. All reagents and solvents were purchased from commercial sources and used without further purification. Acetone, methanol, acetonitrile, 1,4-dioxane, tetrahydrofuran (THF), o-dichlorobenzene, n-butanol, dichloromethane (DCM), 1,8-diazabicyclo-[5,4,0]undec-7-ene (DBU), 1,4-phenylenediacetonitrile (PDAN) and N,N-dimethylformamide (DMF) were purchased from Sinopharm Chemical Reagent Co., Ltd. Anhydrous acetonitrile was dried by Innovative Technology solvent purification system. Dehydrated solvents were obtained after treating solvents with standard procedures. 5,10,15,20-tetrakis(4-benzaldehyde)porphyrin (Por-CHO), two-dimensional metallized porphyrin covalent organic framework (M-Por-COFs) and metallized two-dimensional amidoxime porphyrin-based covalent organic framework (M-Por-COFs-AO) were synthesized according to the routes shown in Scheme S1.

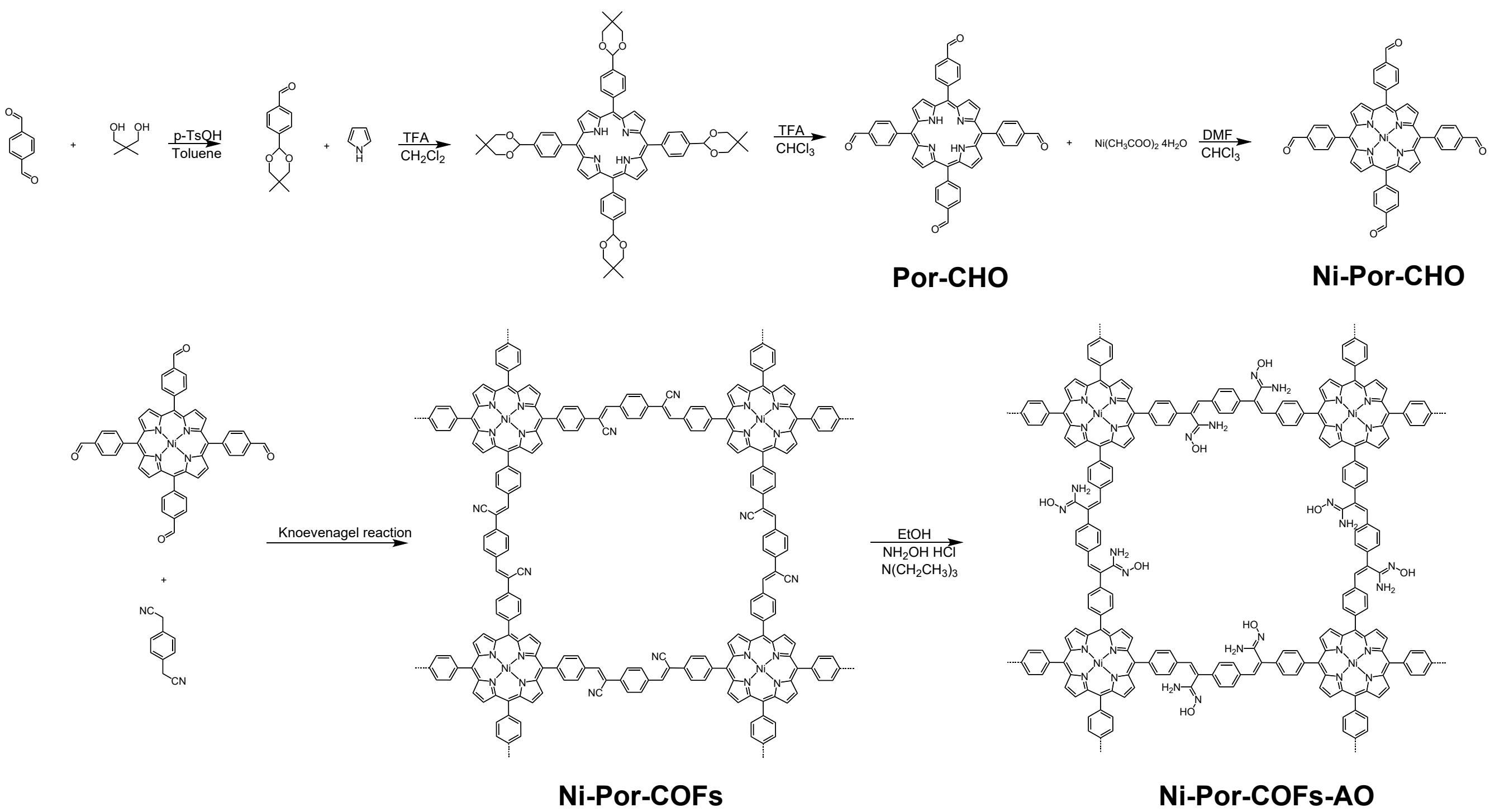

Scheme S1. Synthetic routes of Por-CHO, Ni-Por-COFs and Ni-Por-COFs-AO.

2.2 Methods

2.2.1 Synthesis of *Meso-tetra*(*p*-formylphenyl)porphyrin (Por-CHO)

Step 1 (4-(5,5-Dimethyl-1,3-dioxan-2-yl)benzaldehyde): Neopentyl glycol (8.5 g, 82 mmol), terephthalaldehyde (10.9 g, 82 mmol), and p-toluenesulfonic acid monohydrate (TsOH, 50 mg, 0.3 mmol) were dissolved in 100 mL of toluene in a round-bottom flask (250 mL volume). The mixture was refluxed under stirring and a N_2 atmosphere for 23 h, with the reaction monitored by thin-layer chromatography (TLC). The mixture was then purified by column chromatography using ethyl acetate/petroleum ether (v/v, 1:10) to yield a white product. Yield: 11.5 g, 64%. ^{1}H NMR (400 MHz, $CDCl_3$): 10.02 (s, 1H), 7.89 (d, J = 8.3 Hz, 2H), 7.68 (d, J = 8.2 Hz, 2H), 5.45 (s, 1H), 3.79 (d, J = 11.2 Hz, 2H), 3.67 (d, J = 10.7 Hz, 2H), 1.29 (s, 3H), 0.81 (s, 3H) (Figure S1). ^{13}C NMR (101 MHz, $CDCl_3$): 192.22, 144.73, 136.79, 129.90, 127.08, 100.88, 77.84, 30.45, 23.17, 22.00 (Figure S2).

Step 2 (Tetrakis[4-(1,3-dioxa-5,5-dimethylcyclohex-2-yl)phenyl]porphyrin): Under a N_2 atmosphere, the white product from step 1 (6.74 g, 30.6 mmol) and pyrrole (2.8 mL) were added to dry dichloromethane (720 mL). Trifluoroacetic acid (TFA, 1.9 mL) was then added and the mixture was stirred for 60 min. After adding 2,3,5,6-tetrachloro-1,4-benzoquinone (3.92 g, 15.9 mmol), the reaction mixture (protected from light) was stirred at room temperature under a N_2 atmosphere for 14 h, with the reaction monitored by TLC. Triethylamine (1 mL) was added dropwise to quench the reaction, followed by stirring for 30 min. The solvent was removed under reduced pressure, and the residue was purified by column chromatography using dichloromethane to afford a purple product. Yield: 951 mg, 12%. ^{1}H NMR (400 MHz, $CDCl_3$): 8.81 (s, 8H), 8.20 (d, J = 8.0 Hz, 8H), 7.88 (d, J = 8.0 Hz, 8H), 5.72 (s, 4H), 3.94 (d, J = 10.9 Hz, 8H), 3.82 (d, J = 10.8

Hz, 8H), 1.44 (s, 12H), 0.88 (s, 12H), -2.85 (brs, 2H) (Figure S3). ^{13}C NMR (101 MHz, $CDCl_3$): 143.00, 138.06, 134.62, 126.19, 124.76, 119.88, 102.19, 78.10, 30.58, 23.38, 22.13 (Figure S4).

Step 3 (Por-CHO): The purple product from step 2 (378 mg, 0.35 mmol) was dissolved in chloroform ($CHCl_3$, 40 mL). After adding TFA (18 mL), the resulting solution was stirred at room temperature overnight, with the reaction monitored by TLC. The pH of the mixture was adjusted to 5.0 using a saturated K_2CO_3 solution and stirred for 4 hours. The crude product was washed with water, then separated. The organic phase was dried over anhydrous Na_2SO_4 and concentrated under reduced pressure. The resulting product was washed with methanol and filtered to yield the deep purple aldehyde-functionalized porphyrin (H_2Por-CHO). Yield: 210 mg, 82%. ^{1}H NMR (400 MHz, $CDCl_3$): 10.40 (s, 4H), 8.83 (s, 8H), 8.42 (d, J = 7.9 Hz, 8H), 8.31 (d, J = 7.9 Hz, 8H), -2.84 (s, 2H) (Figure S5). ^{13}C NMR (101 MHz, $CDCl_3$): 192.40, 147.89, 140.89, 140.16, 135.98, 135.28, 128.29, 119.42 (Figure S6).

2.2.2 Synthesis of M-Por-CHO

Synthesis of Ni-Por-CHO: Por-CHO (200 mg, 0.276 mmol) and $Ni(CH_3COO)_2\cdot 4H_2O$ (137.4 mg, 0.55 mmol) were dissolved in DMF (40 mL) and stirred at 120 °C under a N_2 atmosphere for 4 h. After cooling to room temperature, 160 mL of deionized water was added. The resulting precipitate was collected by filtration, washed with methanol, and dried under vacuum to yield a dark red product (Ni-Por-CHO). Yield: 158 mg, 76%. ^{1}H NMR (400 MHz, $CDCl_3$): 10.33 (s, 4H), 8.72 (s, 8H), 8.21 (s, 16H) (Figure S7). ^{13}C NMR (101 MHz, $CDCl_3$): 191.12, 141.28, 134.87, 133.28, 131.38, 127.28, 126.13, 117.13 (Figure S8).

Synthesis of Cu-Por-CHO: Por-CHO (200 mg, 0.276 mmol) and $CuCl_2\cdot 2H_2O$ (94 mg, 0.55 mmol) were dissolved in DMF (40 mL) and stirred at 120 °C under a N_2 atmosphere for 4 h. After cooling to room temperature, 160 mL of deionized water was added. The resulting precipitate was collected by filtration, washed with methanol, and dried under vacuum to yield a dark red product (Cu-Por-CHO).

Synthesis of Co-Por-CHO: Por-CHO (200 mg, 0.276 mmol) and cobalt(II) acetate tetrahydrate ($Co(CH_3COO)_2\cdot 4H_2O$) were dissolved in DMF (40 mL) and stirred at 120 °C under a N_2 atmosphere for 4 h. After cooling to room temperature, 160 mL of deionized water was added. The resulting precipitate was collected by filtration, washed with methanol, and dried under vacuum to yield a dark red product (Co-Por-CHO).

2.2.3 Synthesis of M-Por-COFs

A metalated aldehyde-functionalized porphyrin (69.72 mg, 0.096 mmol), PDAN (30 mg, 0.192 mmol), 1,2-dichlorobenzene (4 mL), and an aqueous solution of DBU (0.4 mL, 5 M) were placed in an ampoule. The mixture was sonicated for 5 min and subjected to three freeze-thaw cycles. The ampoule was then flame-sealed under vacuum using a butane torch and heated at 80 °C for 3 days. The product was washed with dichloromethane, followed by Soxhlet extraction with tetrahydrofuran and dichloromethane for two days.

Finally, the product was dried under vacuum at 80 °C for 12 h to yield the metal-porphyrin covalent organic framework (M-Por-COF, M = Ni, Cu, Co).

2.2.4 Synthesis of M-Por-COFs-AO

M-Por-COF (0.2 g) was dispersed in anhydrous ethanol (20 mL) and sonicated for 10 min. $NH_2OH{\cdot}HCl$ (0.5 g) and triethylamine ($N(CH_2CH_3)_3$, 3 mL) were added. The mixture was stirred at 85 °C for 24 h. After the reaction, the mixture was filtered. The product was washed sequentially with water, ethanol, and methanol. Finally, the product was dried under vacuum at 60 °C for 12 h to yield the amidoximated metal-porphyrin covalent organic framework (M-Por-COFs-AO, M = Cu, Ni, Co).

SIII. Electrochemical measurements

A three-electrode cell was employed to measure transient photocurrent responses (I–t), electrochemical impedance spectra (EIS) and Mott-Schottky plots were recorded on a CHI650E (Shanghai Chenhua, China) electrochemical workstation. The supporting electrolyte is 0.5 M Na_2SO_4 aqueous solution and the light source is 300 W Xe-lamp (400 nm $\leq \lambda \leq$ 800 nm). A platinum plate and an Ag/AgCl electrode were employed as the counter electrode and reference electrode, respectively. The working electrodes were prepared as follows: 5.0 mg of the sample was separately ground with 10 μL of a Nafion (5%) aqueous solution and 50 μL of ethanol to make slurry. The slurry was then coated onto ITO glass electrodes with an active area of 0.25 cm^2, and these electrolytes were dried at 120 °C for 1 h to evaporate the solvent in muffle furnace. The photocurrent intensity of as-prepared electrodes was measured at 0 V versus Ag/AgCl with the light on and off. EIS was determined over the frequency range of 102–106 Hz with an ac amplitude of 10 mV at the open circuit voltage under room-light illumination. With reference to the Mott–Schottky plots, the flat-band potentials of the samples were converted according to the following relationship: E(NHE) = E(Ag/AgCl) + 0.197, where 0.197 V is the standard potential difference between the Ag/AgCl electrode and the NHE.

SIV. U(VI) photocatalytic tests.

4.1 Photocatalytic reduction of U(VI)

To evaluate the photocatalytic reduction effect on the uranium adsorption capacity, a 300 W Xe lamp with an irradiance of 1 kW/m^{-2} was utilized as the simulated sunlight. A dark condition was used for the control experiment.

4.2 Uranium sorption kinetics test

An appropriate amount of $UO_2(NO_3)_2{\cdot}6H_2O$ was dissolved in deionized water to prepare the U(VI) solutions. The pH of the U(VI) solution was regulated to 6.0 unless otherwise specified. Photocatalysis experiments were carried out by circulating cooling water to ensure the reaction temperature was close to

room temperature (T = 298.15 K). To determine the uranium adsorption ability, a uranyl nitrate solution with a concentration of 20 ppm U(VI) was used. The adsorbent with a dry weight of 5 mg was added into 150 mL of 20 ppm U(VI) solution and allowed to adsorb with moderate stirring at 25 °C. At each time point, an aliquot was taken out and analyzed using Arsenazo III assay to determine the concentration of U(VI). The uranium sorption capacity was calculated using the following equation:

$$q_t = \frac{(C_0 - C_t) \times V}{m} \qquad \text{(S1)}$$

where q_t (mg-U per g-Ads) is the uranium sorption amounts at exposure time (t); C_0 and C_t (mg L^{-1}) is the uranium concentrations at exposure time 0 and t, respectively; V is the volume (L) and m is the adsorbent mass (g). At equilibrium, q_t and C_t are replaced by q_e and C_e, respectively.

Natural seawater used in the continuous-flow experiment was commercially sourced from the East China Sea. The seawater was pretreated by coarse quartz-sand filtration to remove suspended particulates and most plankton, followed by powdered activated carbon treatment and 0.45 μm membrane filtration. It was stored at 4 °C after receipt and used within 5 days. During operation, the seawater had a pH of 8.1–8.2 and a salinity of 32–34, and the experiment was conducted at 25 ± 2 °C. The continuous-flow reactor was irradiated from the top by a 300 W Xe lamp with a light intensity of 1 kW m^{-2}. The lamp was positioned approximately 50 cm above the reactor. Illumination was applied from one side only, and the light passed through the quartz plate to the Ni-Por-COF-AO-coated microchannel region, covering nearly the entire 8 × 8 cm^2 reactor plate. Outlet samples were collected every 24 h during the 7-day continuous-flow experiment.

For ppb-level uranium quantification in natural seawater, the outlet samples were analyzed by external Thermo Scientific iCAP Q ICP-MS. A low-level calibration curve in the range of 0–10 ppb was used. The calibration blank was 2% HNO_3 in ultrapure water. Prior to ICP-MS measurement, samples were filtered through a 0.45 μm membrane, acidified to 1–2% HNO_3, and diluted when necessary to ensure that the measured concentration remained within the calibration range. Quality-control measurements included spike-recovery tests, which gave recoveries in the range of 95–105%, and all measurements were conducted in triplicate (n = 3).

The cumulative uranium uptake in the continuous-flow experiment was calculated by summing the uranium removed over each sampling interval according to

$$q_{cum} = \frac{\sum_i (C_{in} - C_{out,i}) Q \Delta t_i}{m} \qquad \text{(S2)}$$

where C_{in} is the inlet uranium concentration in natural seawater, $C_{out,i}$ is the outlet uranium concentration at the i-th sampling interval, Q is the flow rate, Δt_i is the sampling interval, and m is the mass of Ni-Por-COF-AO loaded in the reactor.

The cumulative uranium uptake reached 234 mg g^{-1} over the 7-day continuous-flow seawater experiment. This value was calculated from Eq. (S2) by integrating the uranium removed during each 24 h sampling interval and normalizing the total amount of uranium captured to the mass of Ni-Por-COF-AO loaded in the reactor.

The 92% retention value was obtained from the adsorption-elution recyclability test and is independent of the cumulative-flow calculation. Capacity retention was calculated as q_N/q_1 x 100%, where q_1 and q_N are the adsorption capacities in the first and final tested adsorption-elution cycles, respectively. Thus, 234 mg g^{-1} represents the total uranium captured in the continuous-flow seawater test, whereas 92% describes the retained adsorption capacity after regeneration.

4.3 Equilibrium sorption isotherm test

150 mL of U(VI) solutions with different initial concentrations of uranium (5, 10, 20, 40, 60, 80, 100, 120 ppm) were prepared for equilibrium sorption isotherm experiments. After being stirred at room temperature for 14 h, aliquots were taken out and analyzed by Arsenazo III assay.

4.4 Effect of competing ions on the photocatalytic uranium extraction

In the selectivity test, ASTM D1141-98 artificial seawater was used as the background electrolyte. The experimental conditions were based on the equilibrium sorption protocol, except that competing metal ions were simultaneously introduced into the photocatalytic system. Specifically, U(VI), V, Cu, Fe, Zn, and Co were each fixed at 5 ppm, and V was introduced as $NaVO_3$. The pH was adjusted to 8, and the experiment was performed under visible-light irradiation from a 300 W Xe lamp with a light intensity of 1 kW m^{-2}. The selectivity coefficient $S_{U/V}$ was calculated from the ratio of the adsorption capacities of U and V under the same conditions. Supplemental experiments on Ni-Por-COF-AO further showed that HCO_3^-, SO_4^{2-}, Cl^-, and ionic strength influence the absolute uranium uptake and $S_{U/V}$ value to different extents. Among these factors, bicarbonate shows the most noticeable effect, whereas chloride and sulfate lead to comparatively smaller changes. An increase in ionic strength also causes a certain attenuation in uptake/selectivity. Nevertheless, Ni-Por-COF-AO retains a clear preference for uranium over vanadium under all tested conditions. These additional results are summarized in Figure S21.

4.5 pH-dependent uranium adsorption in U-spiked aqueous solution

5 mg adsorbents were put into 100 mL of deionized water containing 20 ppm uranyl ions. The pH of the aqueous solution was adjusted by using 0.1 M NaOH and 0.1 M HNO_3 solutions to 2.0, 3.0, 4.0, 5.0, 6.0, 7.0, 8.0, 9.0, respectively. Uranium adsorption amount was calculated using Equation (S1).

4.6 Elution experiments

After being sufficiently dried, the used photocatalysts were placed in a solution of 0.1 M HNO_3. For each 5 mg adsorbents, 50 mL elution solution was used to elute the binding uranium for 90 min at room temperature. The resulting suspension was filtered and washed with ultra-pure water till the supernatant became neutral. After being dried under vacuum, the resultant material was used for another adsorption experiment. Equation (S3) can be used to calculate the elution efficiency (E_E, %):

$$E_E = \frac{C_E \times V_E}{W_E} \times 100\% \qquad \text{(S3)}$$

where C_E (mg L^{-1}) stands for the uranium content in the eluate. W_E (mg) stands for the amount of uranium adsorbed, V_E (L) stands for the volume of the eluent.

For recyclability evaluation, the adsorption-elution procedure was repeated under the same adsorption and elution conditions. The capacity retention after regeneration was calculated as q_N/q_1 x 100%, where q_1 and q_N denote the adsorption capacities in the first and final tested cycles, respectively. This retention metric evaluates reusable capacity after acid elution and should not be summed with the cumulative uranium uptake from the continuous-flow seawater experiment.

4.7 Anti-biofouling measurements

Anti-biofouling measurements were carried out according to a reported literature procedure.[1] The bacterial strains *Staphylococcus aureus*, *Streptococcus pneumoniae*, *Pseudomonas aeruginosa* were used to test the antimicrobial spectrum of the adsorbents. Exponentially growing bacterial cultures and the adsorbents were transferred into fresh LB broth at a ratio of 1% (V/V) and 0.05% (m/V), respectively. After cultivation at 37 °C for 4 h with moderate shaking (180 rpm), the viability of the bacterium was determined. The dilution plate counting method was used according to the Chinese standard GB/T20944 to determine the viability of the adsorbents treated bacterial cultures. In brief, sterilized LB solid medium was poured into the aseptic plates to prepare sterile plate count agar plates under aseptic conditions and bacterial cultures were spread onto the plate after gradient dilution. After cultivating overnight at 37 °C, the number of viable bacterial cells was determined by counting the bacterial colonies formed on the plate. The bacterial cultures without adsorbent treatment were used as control. The inhibition rate was calculated using the Equation IR = $(C_i - C_a)/C_i \times 100$, where C_a (CFU/mL) indicates the concentration of bacterial cultures treated with adsorbent and C_i (CFU/mL) indicates the concentration of bacterial cultures without treatment. The simulated sunlight with a light density of 1 kW m^{-2} was used to illuminate the adsorbents.

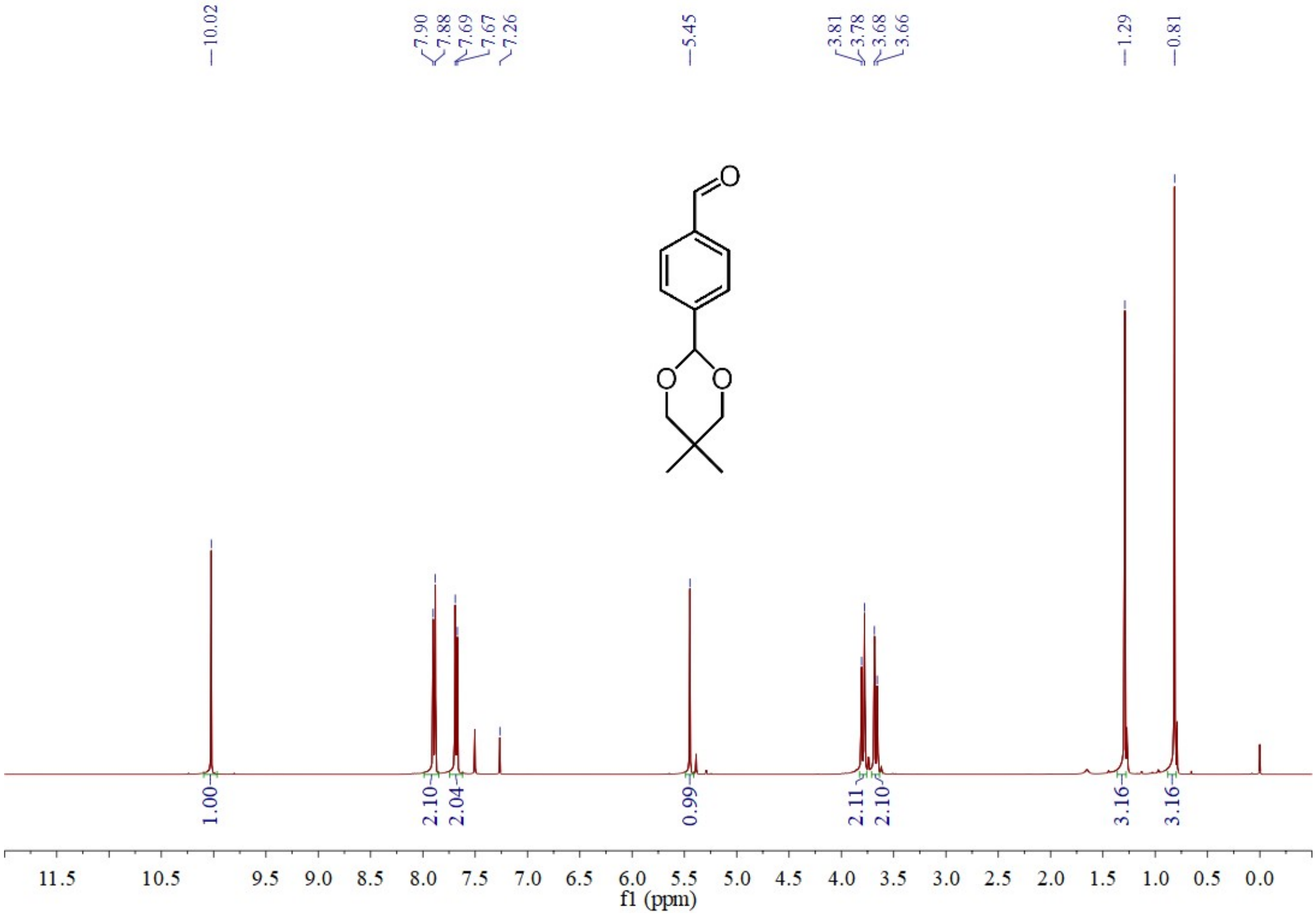

Figure S1. ^{1}H NMR spectrum (in CDCl3) of the 4-(5,5-dimethyl-1,3-dioxan-2-yl)benzaldehyde.

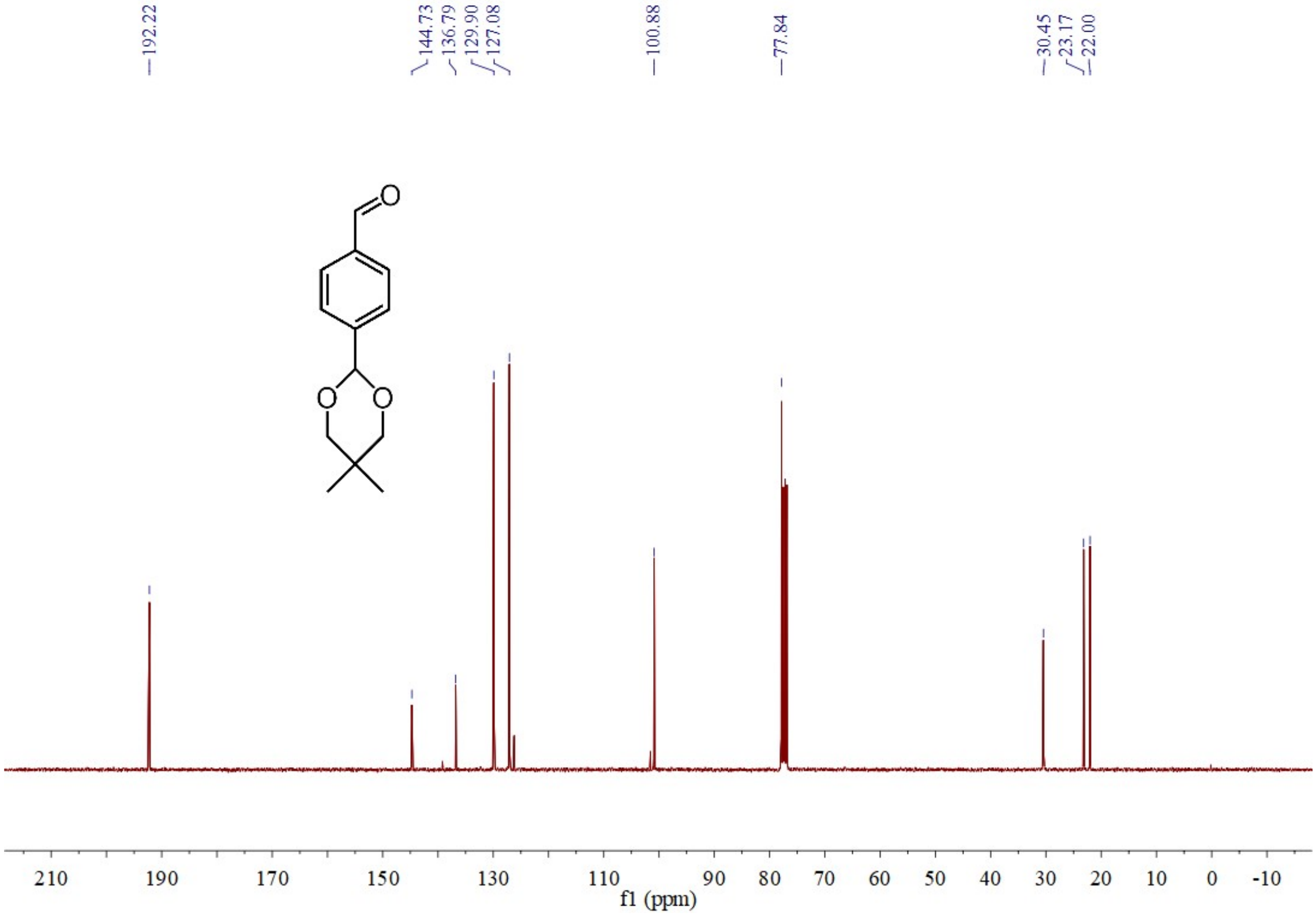

Figure S2. ^{13}C NMR spectrum (in $CDCl_3$) of the 4-(5,5-dimethyl-1,3-dioxan-2-yl)benzaldehyde.

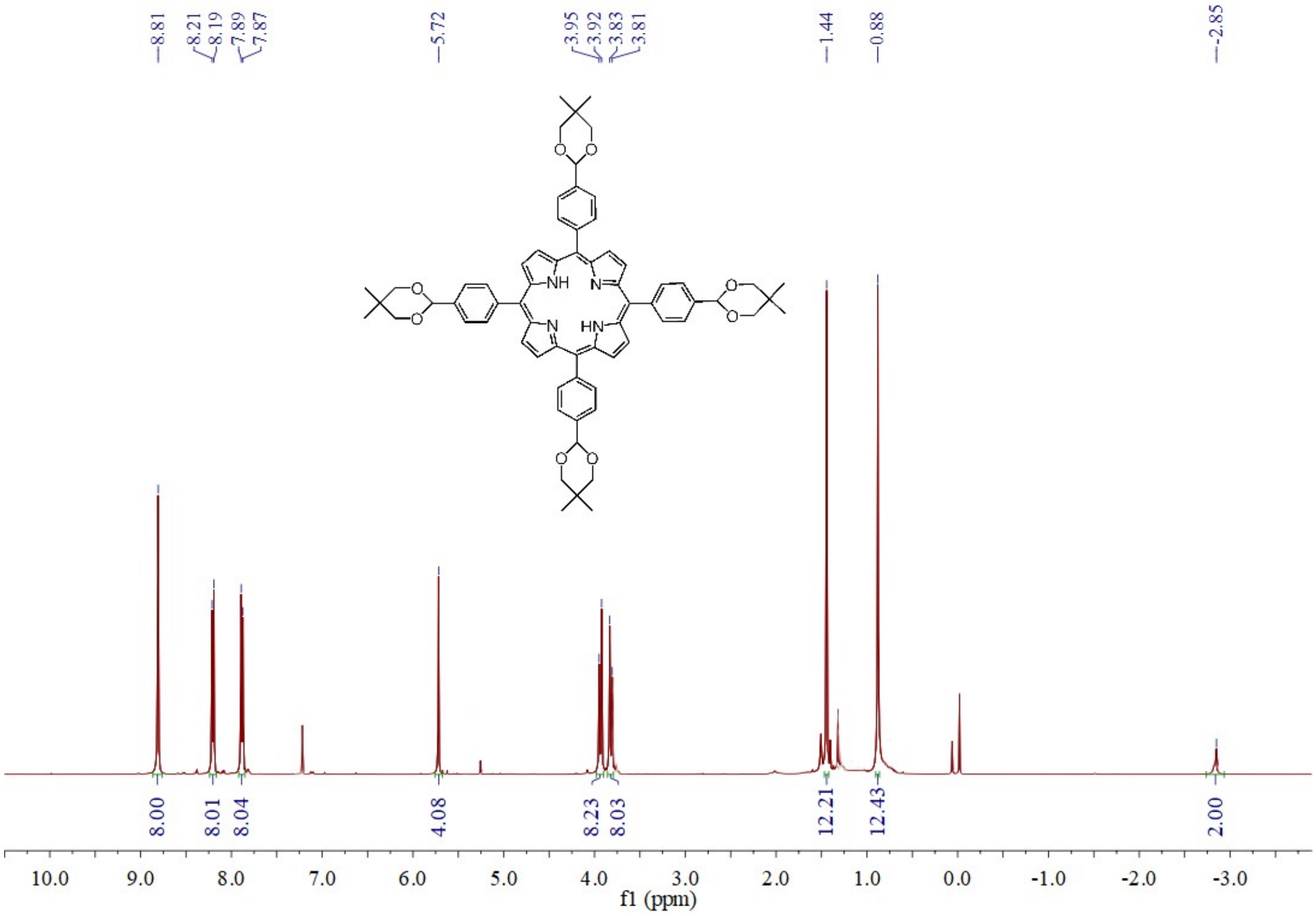

Figure S3. ^{1}H NMR spectrum (in $CDCl_3$) of the tetrakis[4-(1,3-dioxa-5,5-dimethylcyclohex-2-yl)phenyl] porphyrin.

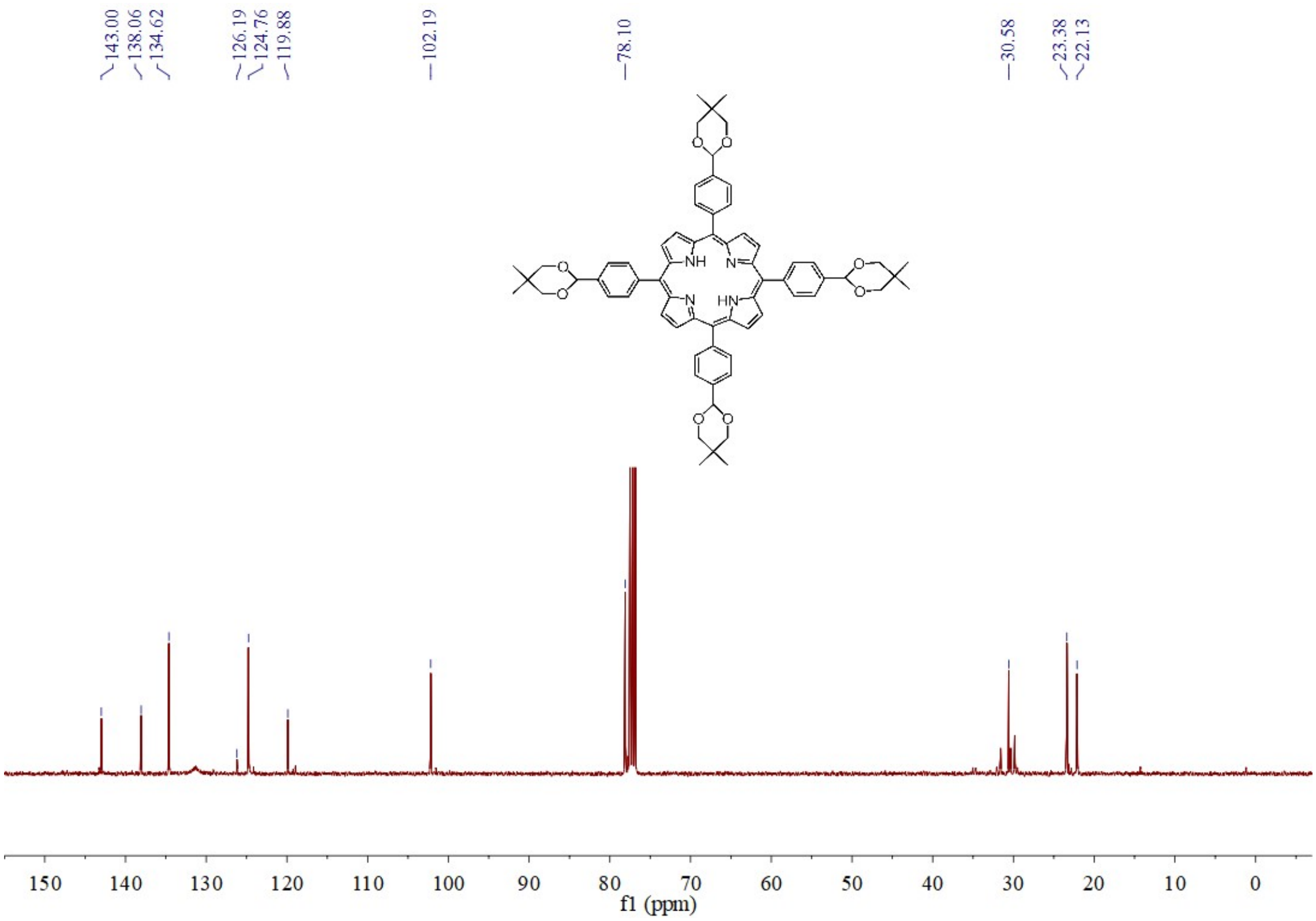

Figure S4. ^{13}C NMR spectrum (in $CDCl_3$) of the tetrakis[4-(1,3-dioxa-5,5-dimethylcyclohex-2-yl)phenyl] porphyrin.

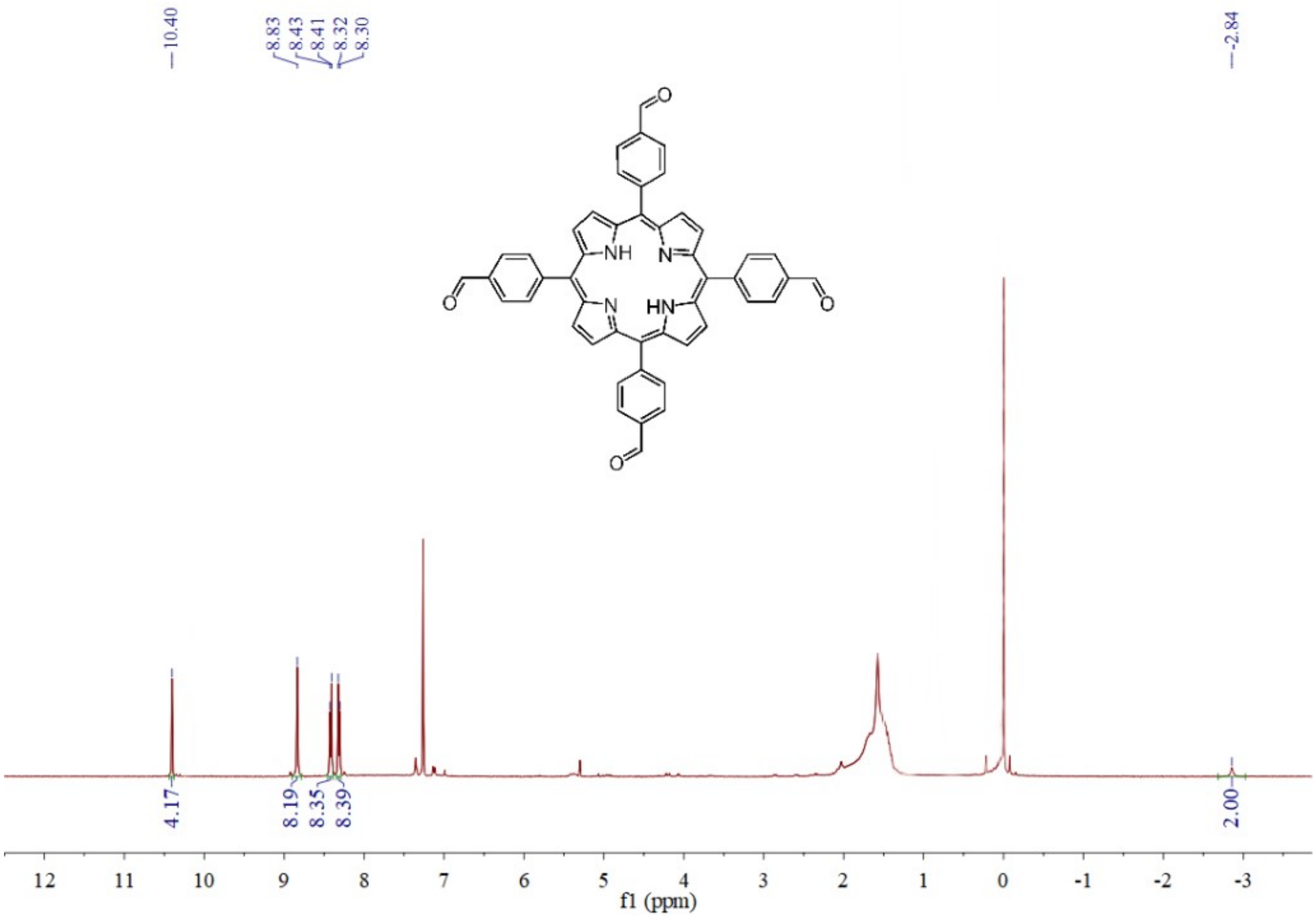

Figure S5. ^{1}H NMR spectrum (in $CDCl_3$) of the Por-CHO.

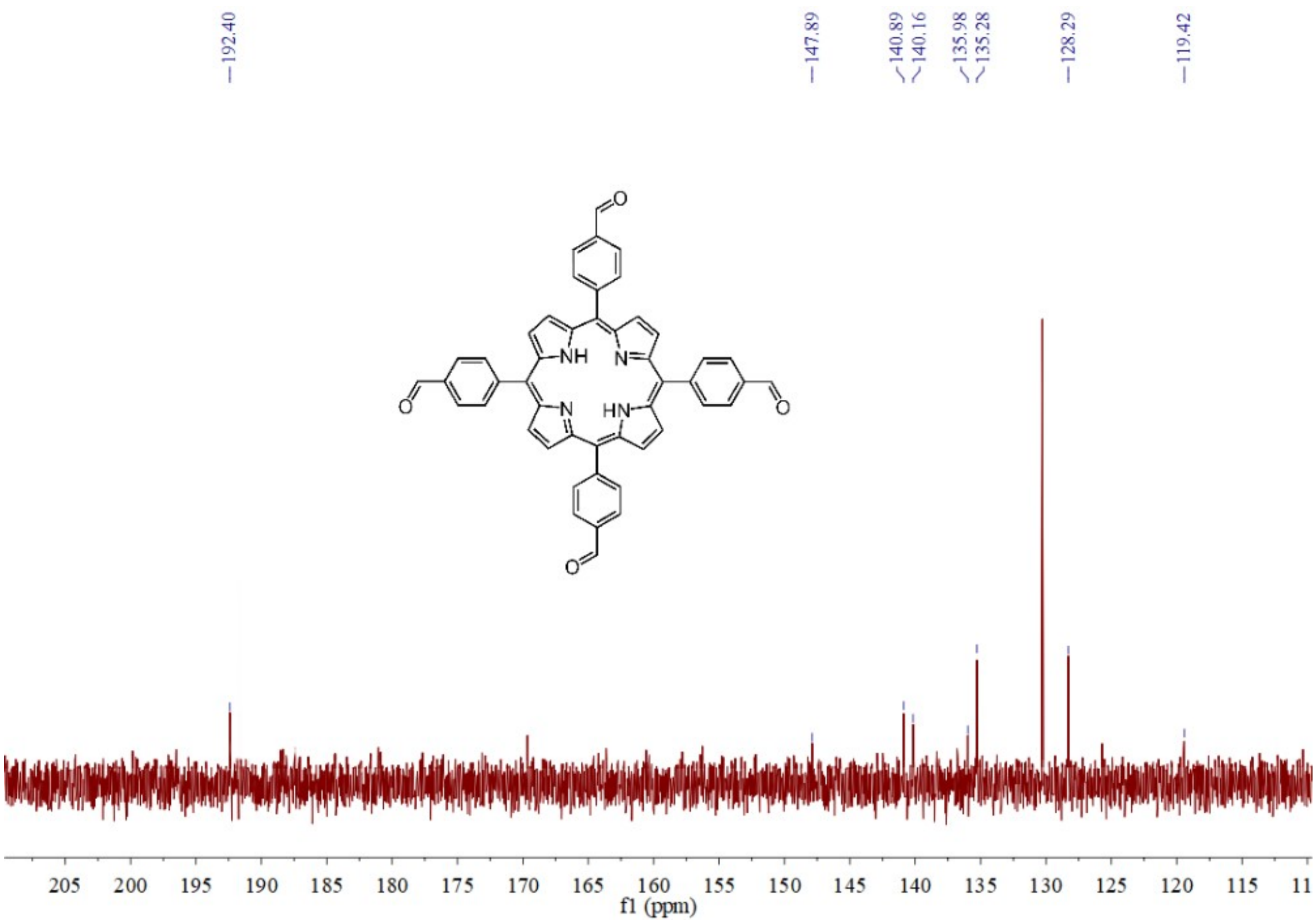

Figure S6. ^{13}C NMR spectrum (in $CDCl_3$) of the Por-CHO.

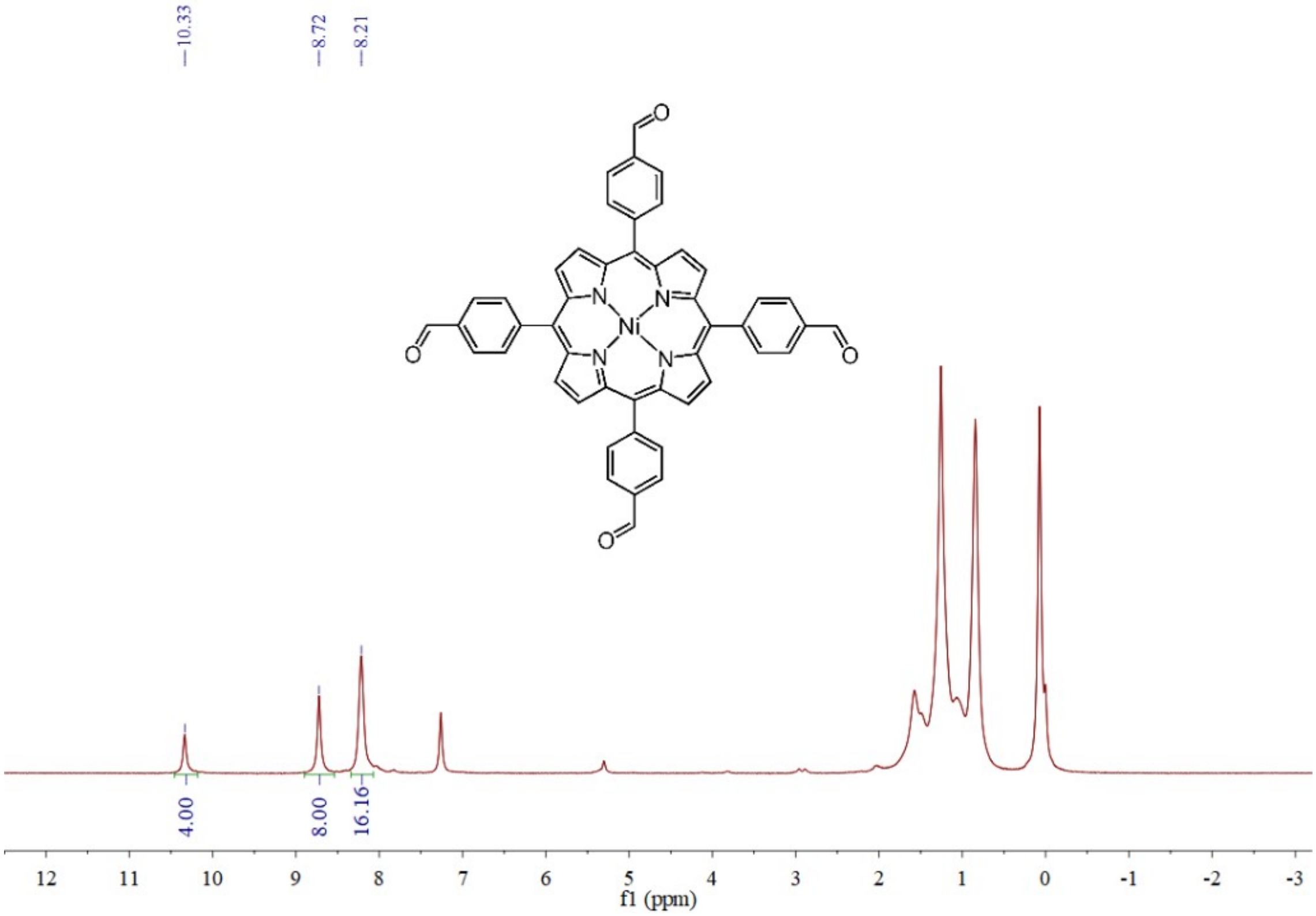

Figure S7. ^{1}H NMR spectrum (in $CDCl_3$) of the Ni-Por-CHO.

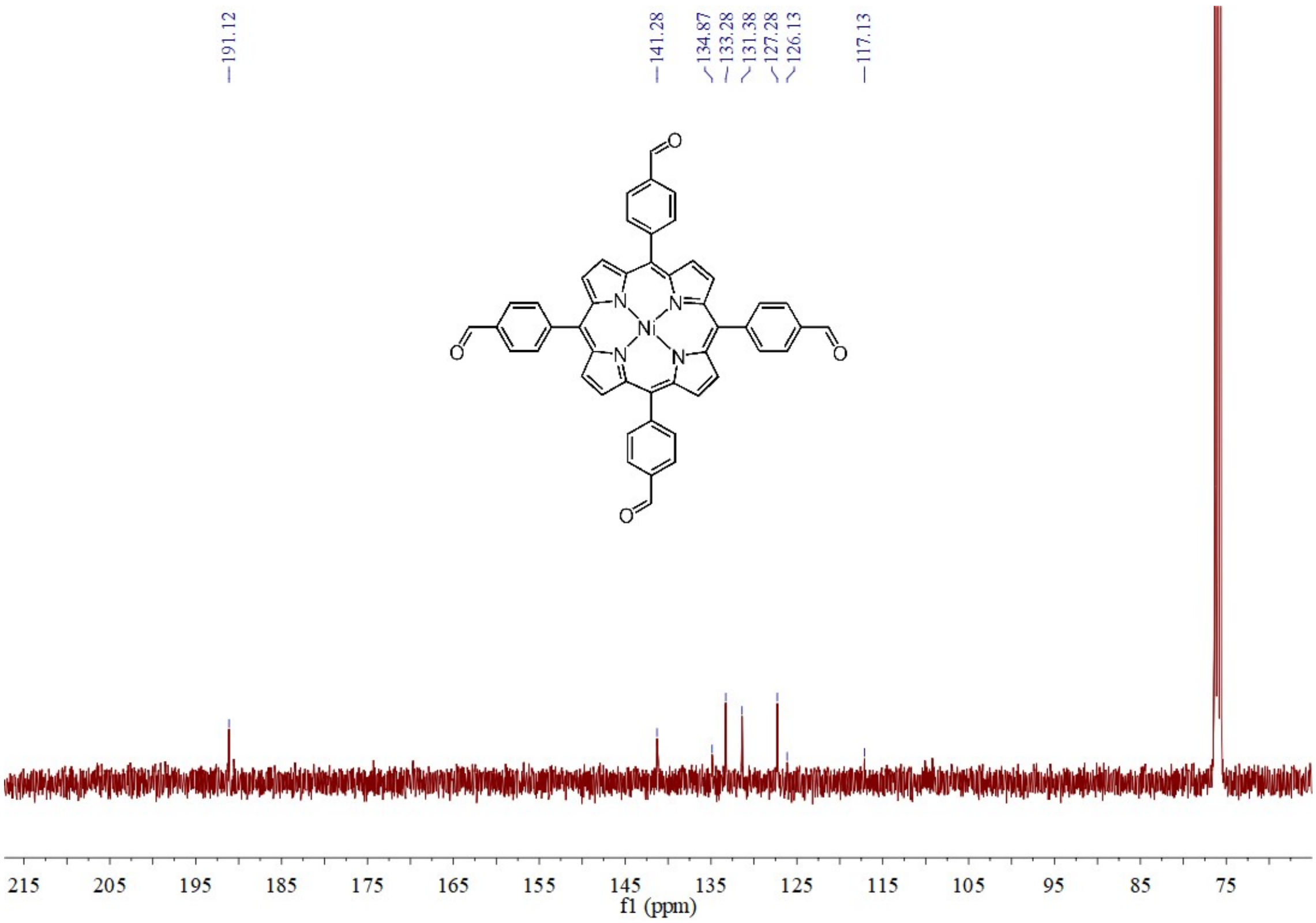

Figure S8. ^{13}C NMR spectrum (in $CDCl_3$) of the Ni-Por-CHO.

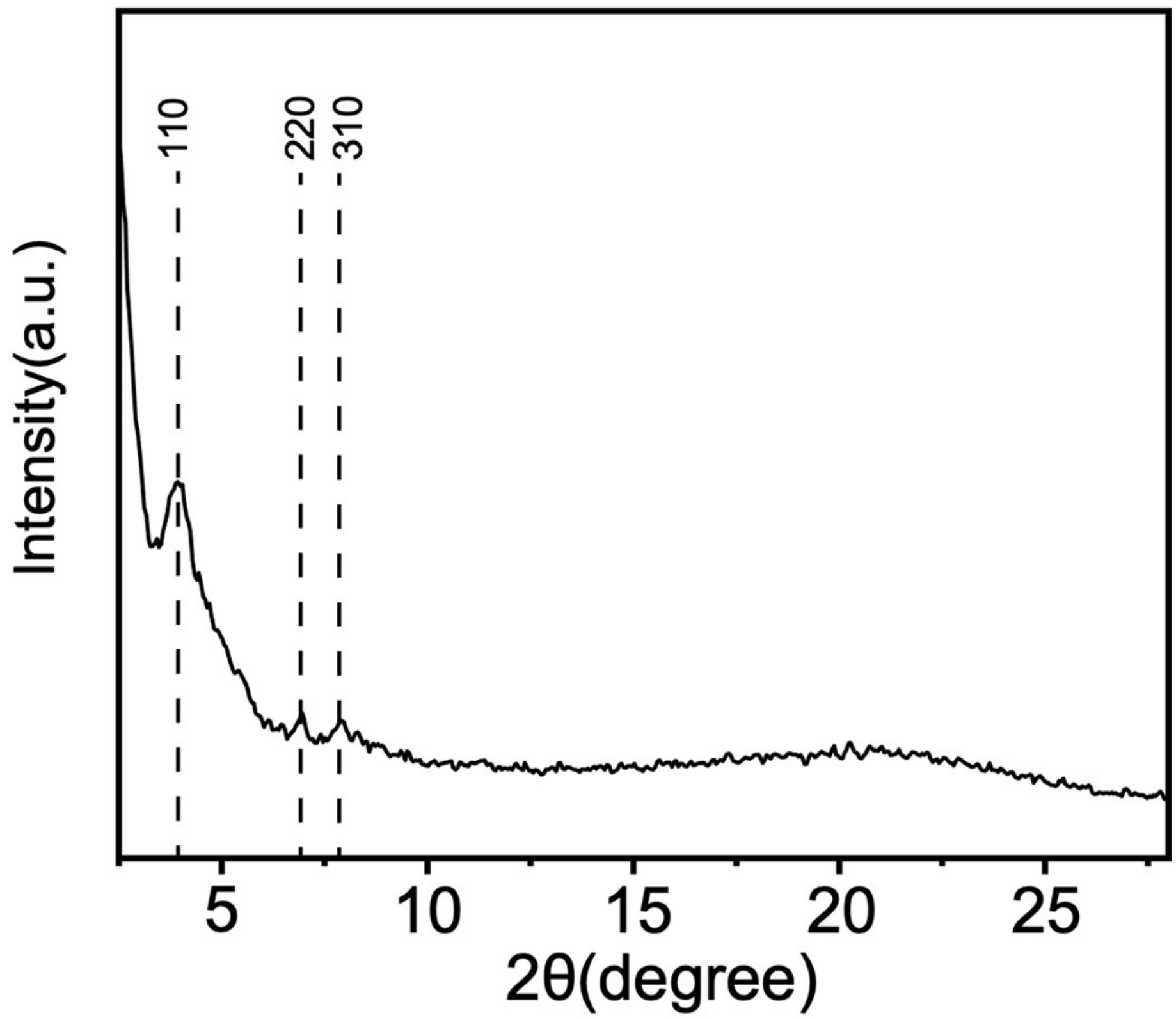

Figure S9. PXRD pattern of the unmodified Por-COF.

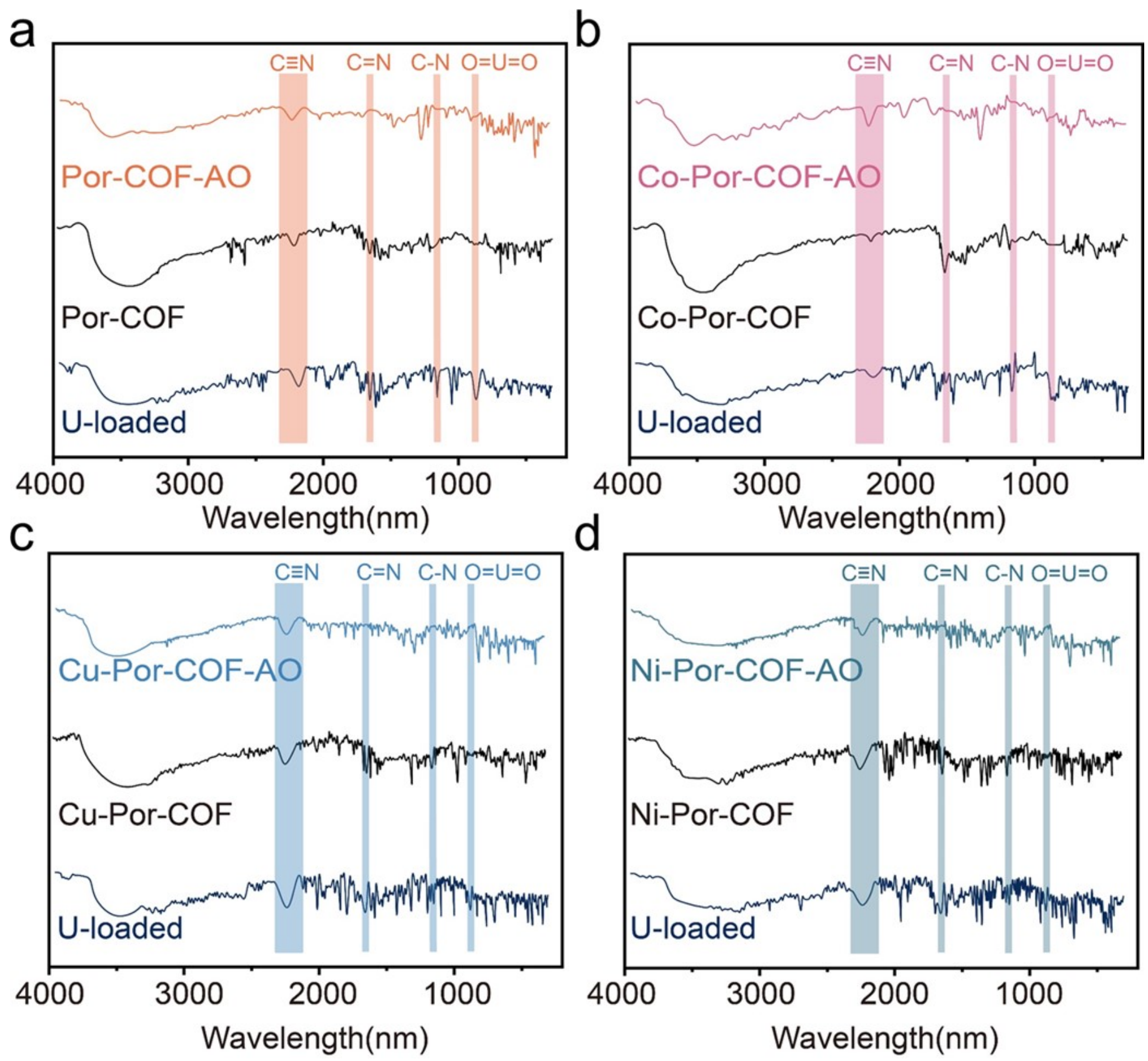

Figure S10. FT-IR spectra of the four samples before and after grafting the amidoxime groups.

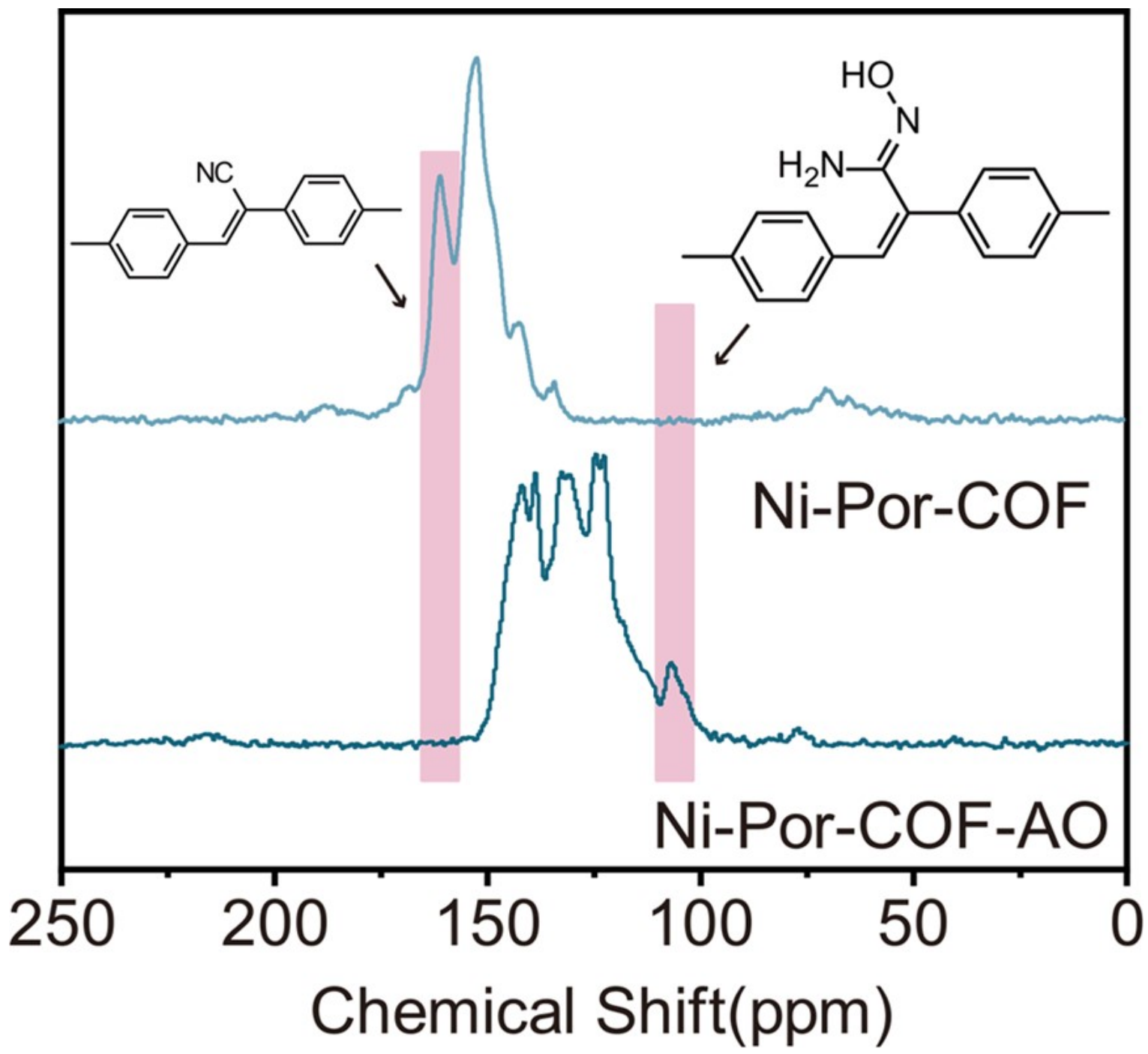

Figure S11. Solid-state ^{13}C CP-MAS NMR spectra of Ni-Por-COFs and Ni-Por-COFs-AO.

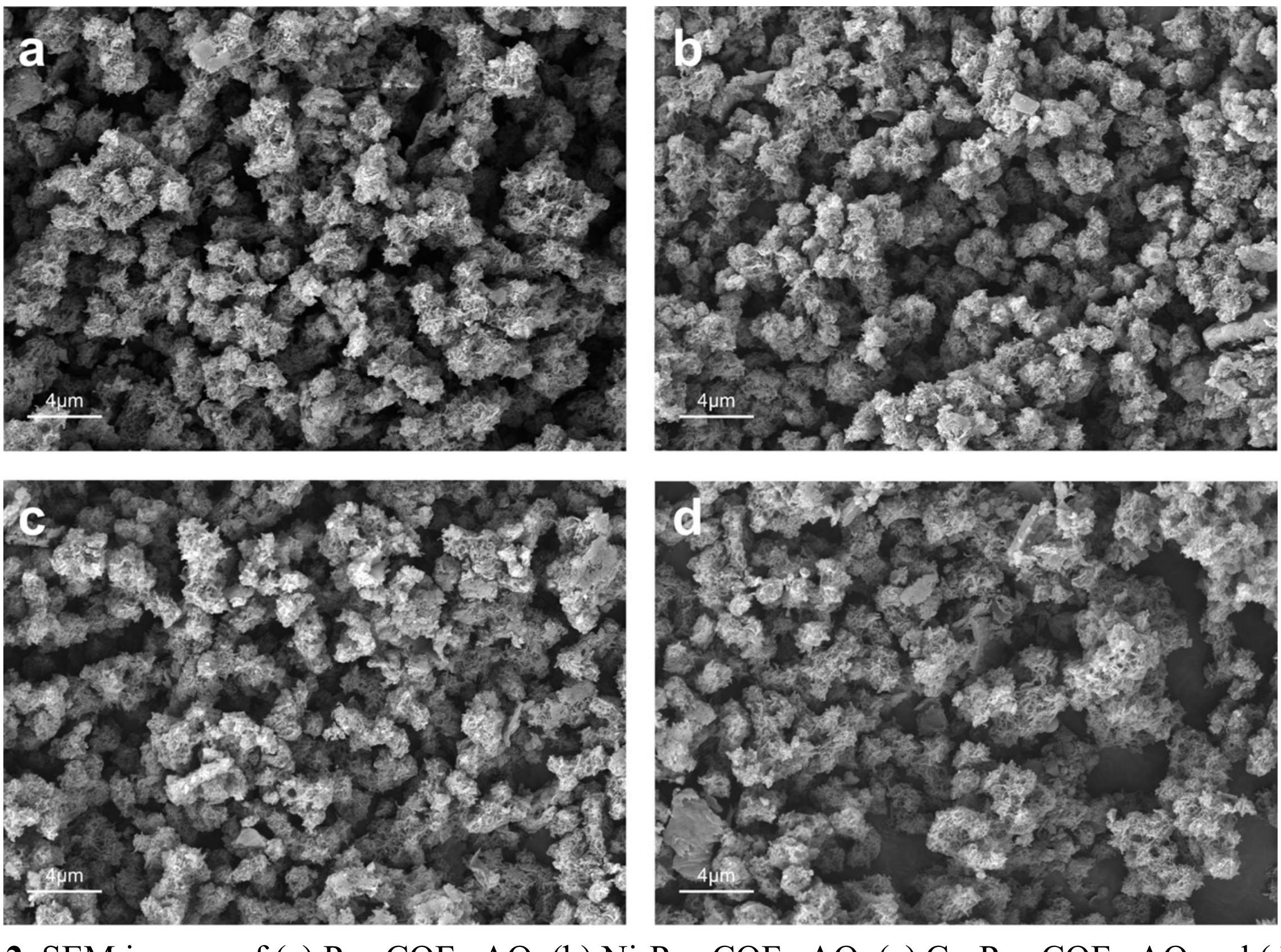

Figure S12. SEM images of (a) Por-COFs-AO, (b) Ni-Por-COFs-AO, (c) Cu-Por-COFs-AO and (d) Co-Por-COFs-AOs

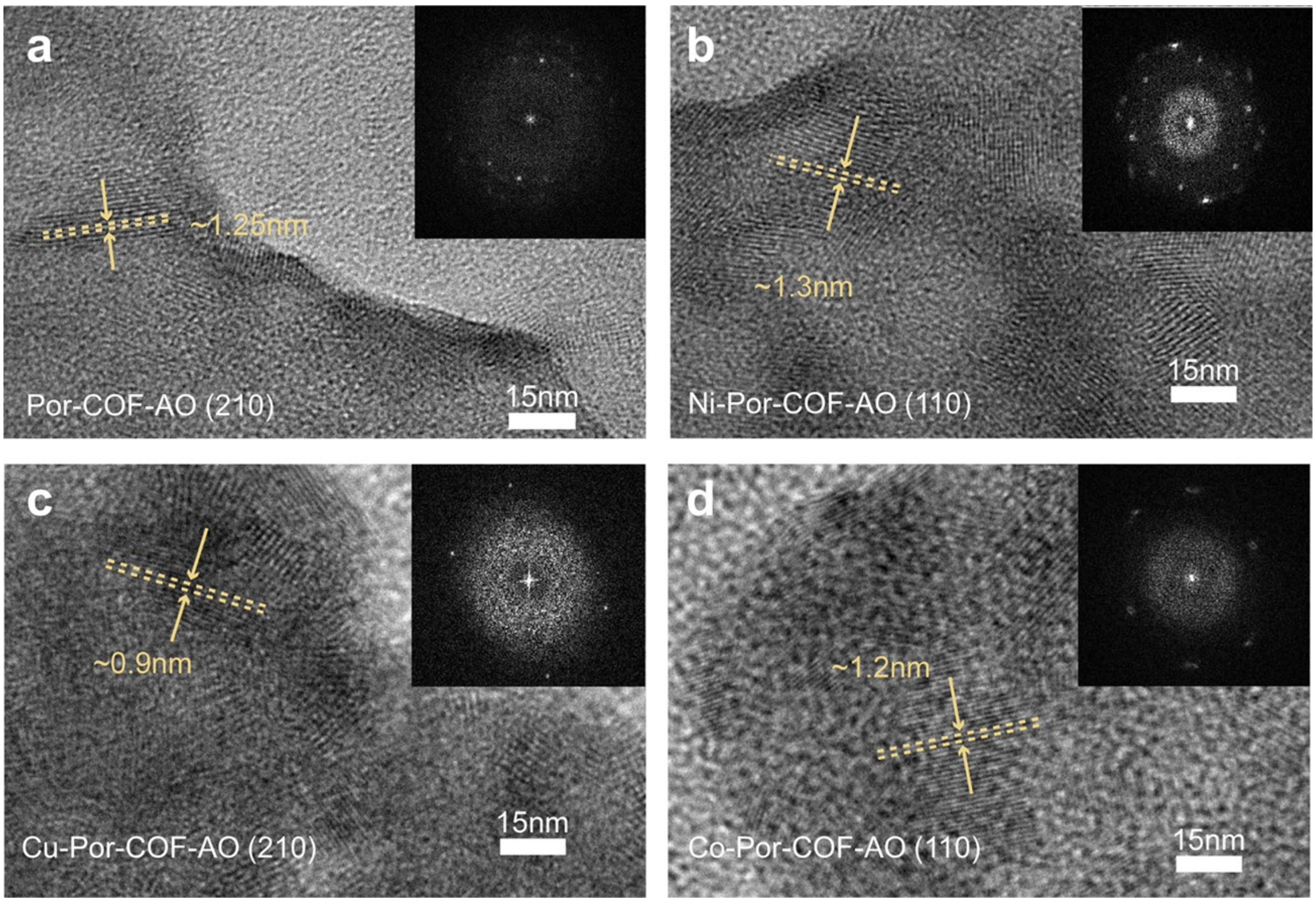

Figure S13. HRTEM images of (a) Por-COFs-AO, (b) Ni-Por-COFs-AO, (c) Cu-Por-COFs-AO and (d) Co-Por-COFs-AO.

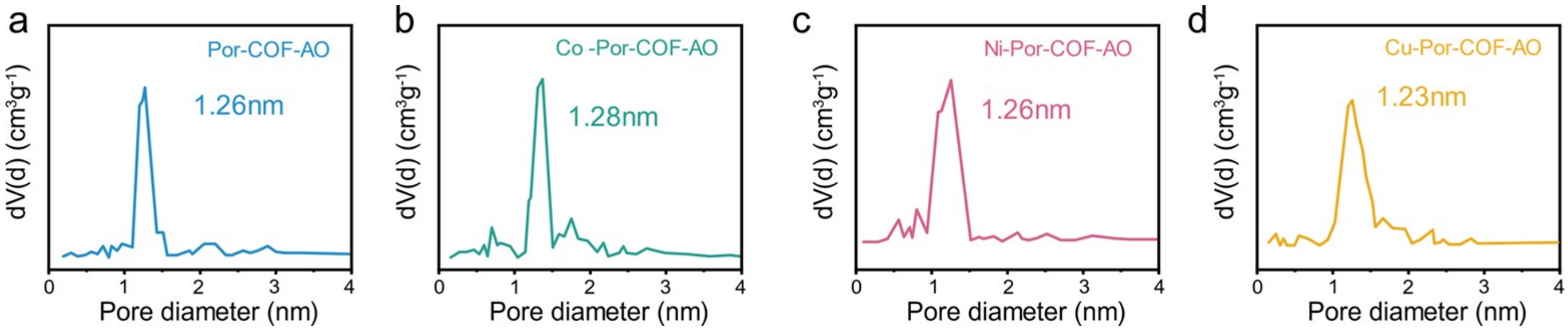

Figure S14. Pore size distribution diagram of (a) Por-COFs-AO, (b) Co-Por-COFs-AO, (c) Ni-Por-COFs-AO and (d) Cu-Por-COFs-AO.

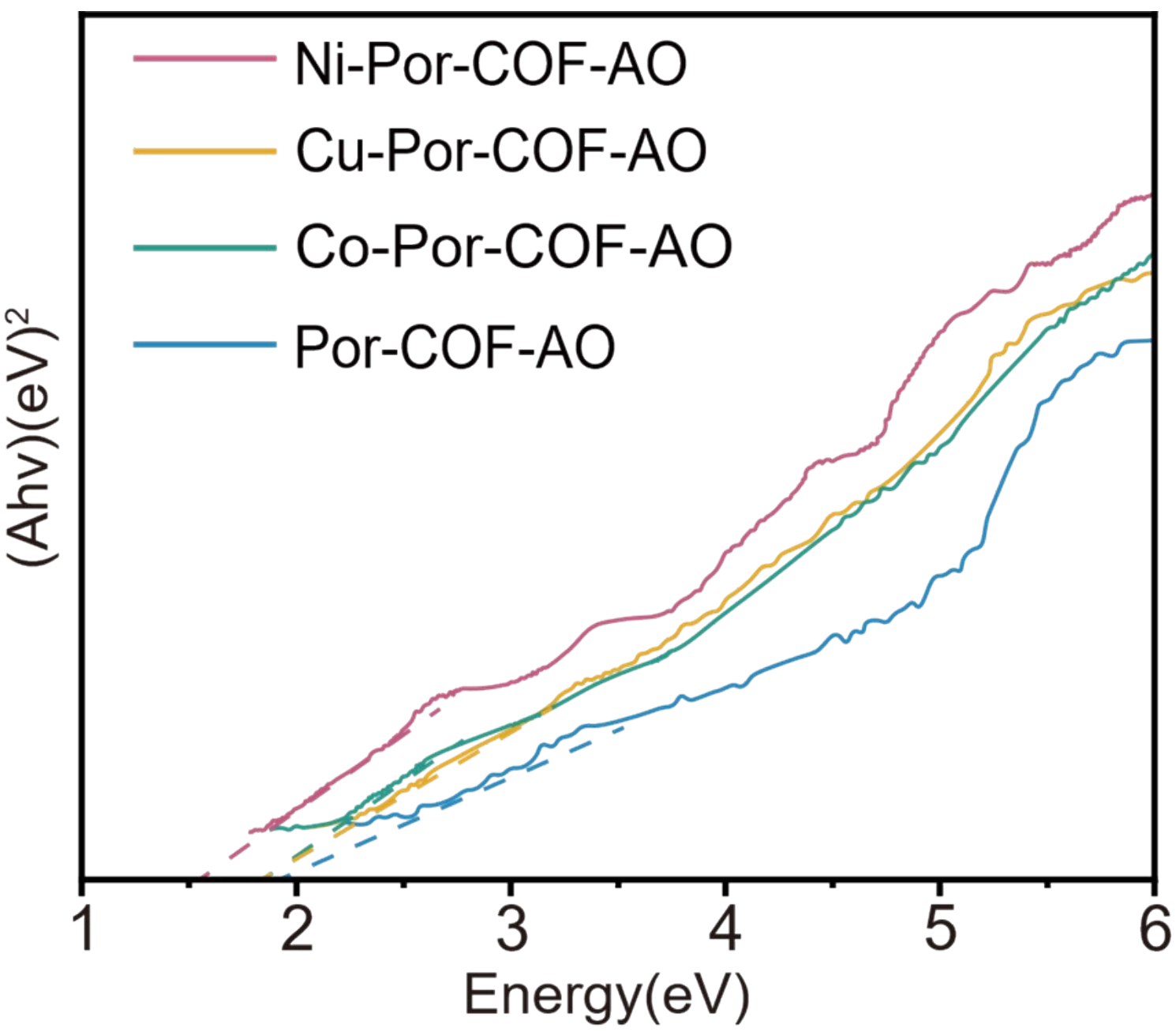

Figure S15. Band gap calculated from the Kubelka–Munk-transformed equation.

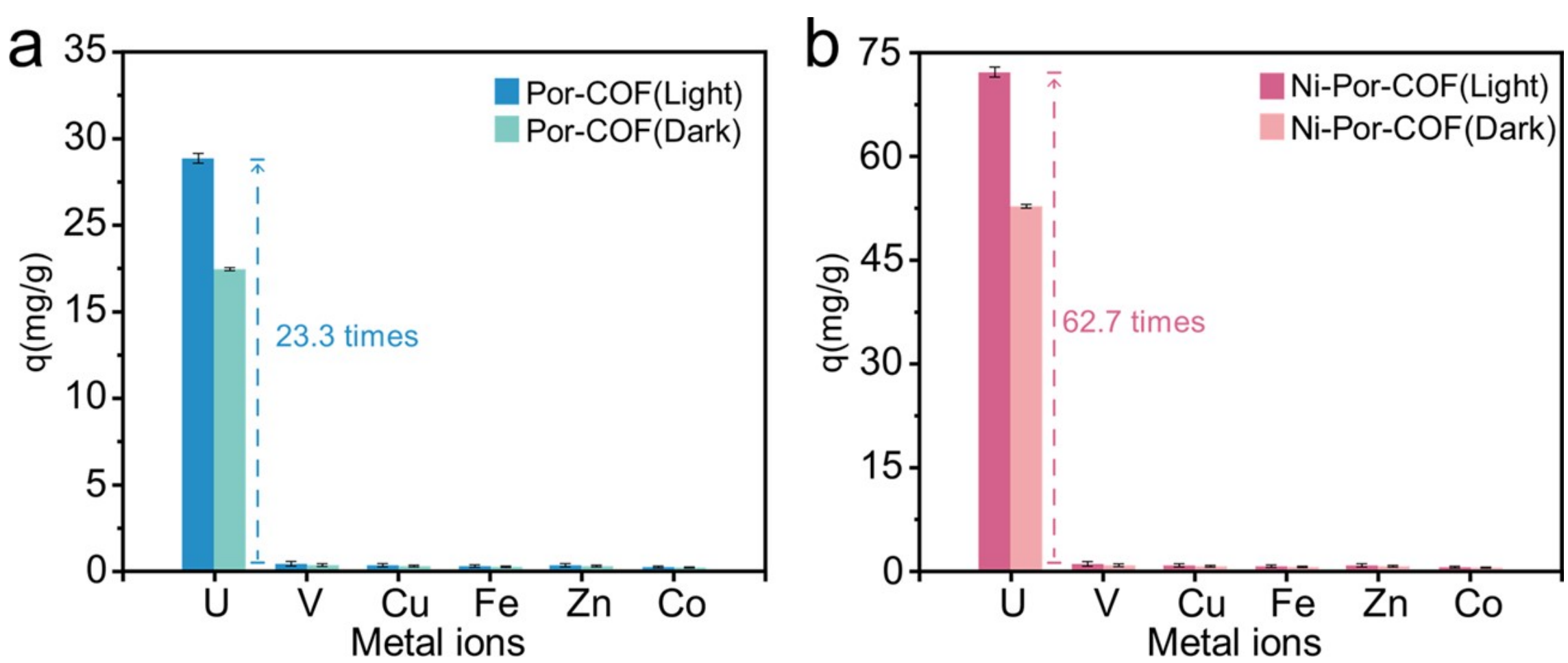

Figure S16. Comparison in ion extraction toward uranium over Fe, Cu, Zn, Co and V for (a) Por-COFs-AO and (b) Ni-Por-COFs-AO.

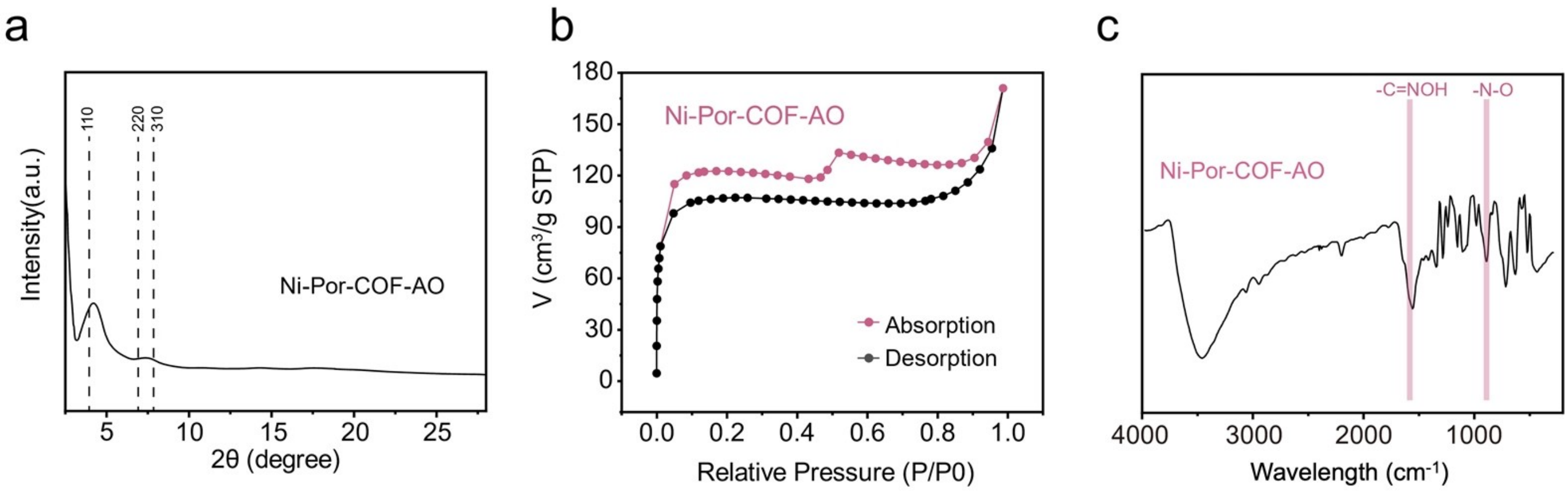

Figure S17. Post-cycle structural characterization of Ni-Por-COF-AO: (a) PXRD patterns after cycling; (b) N_2 adsorption–desorption isotherm of the cycled sample; (c) FT-IR spectra after cycling.

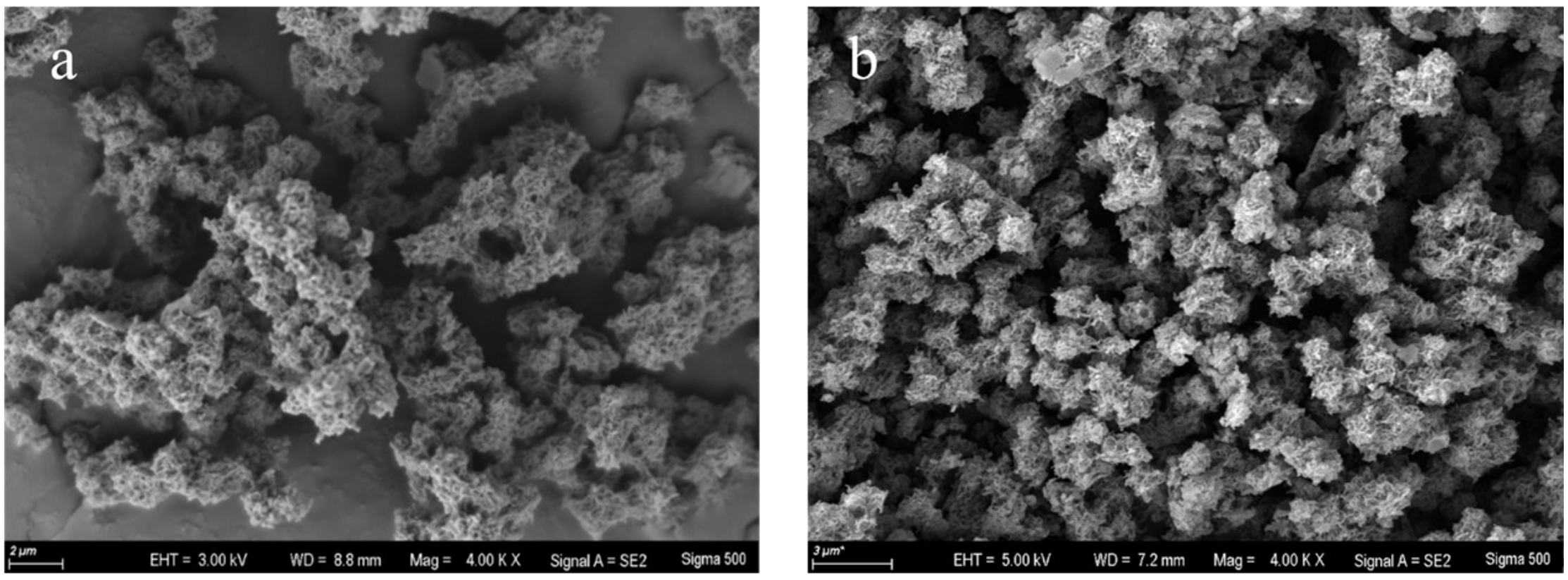

Figure S18. SEM images of Ni-Por-COF-AO after adsorption of uranyl ions.

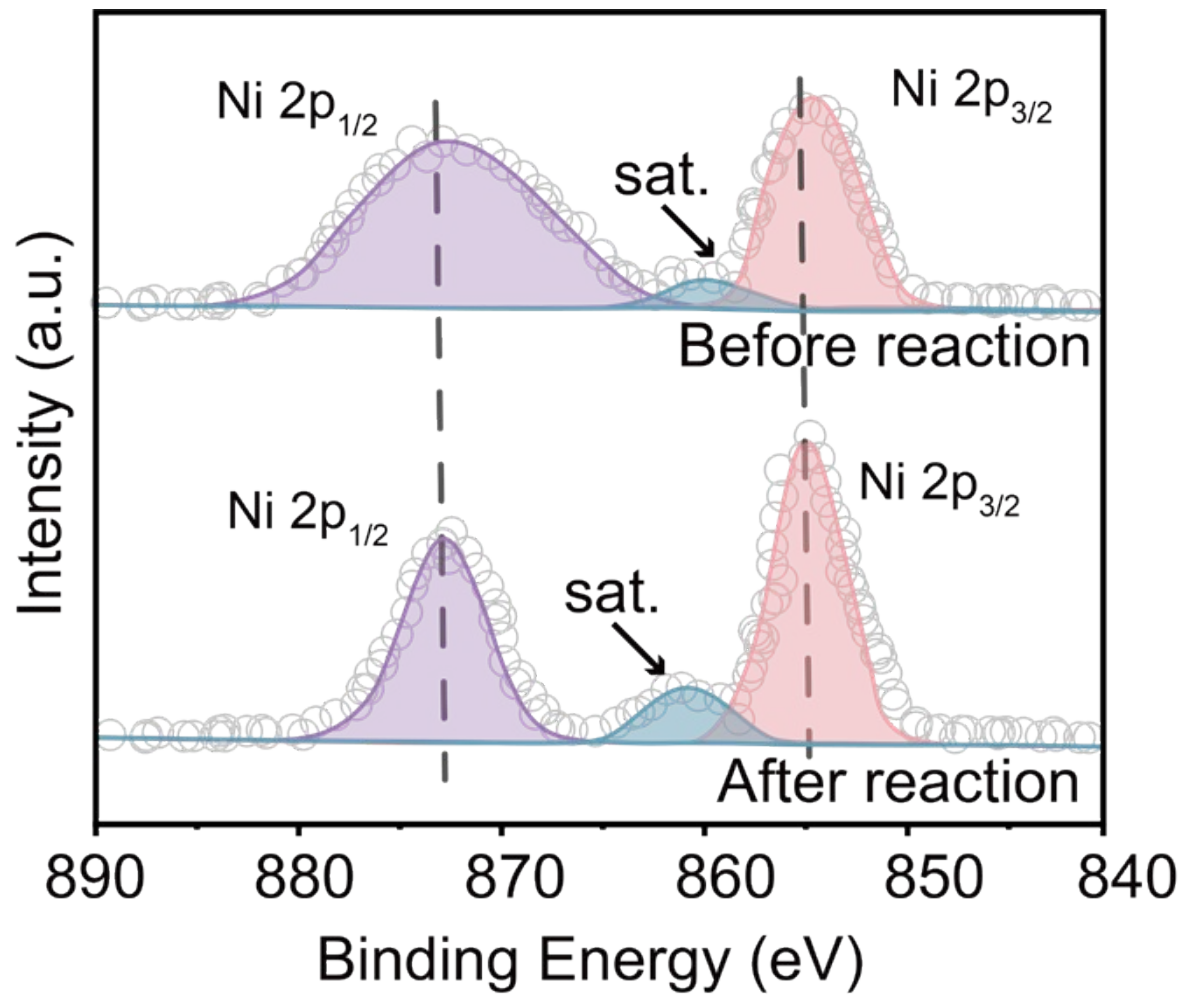

Figure S19. High-resolution Ni 2p XPS spectra of Ni-Por-COF-AO before and after the adsorption–photocatalytic uranium extraction reaction.

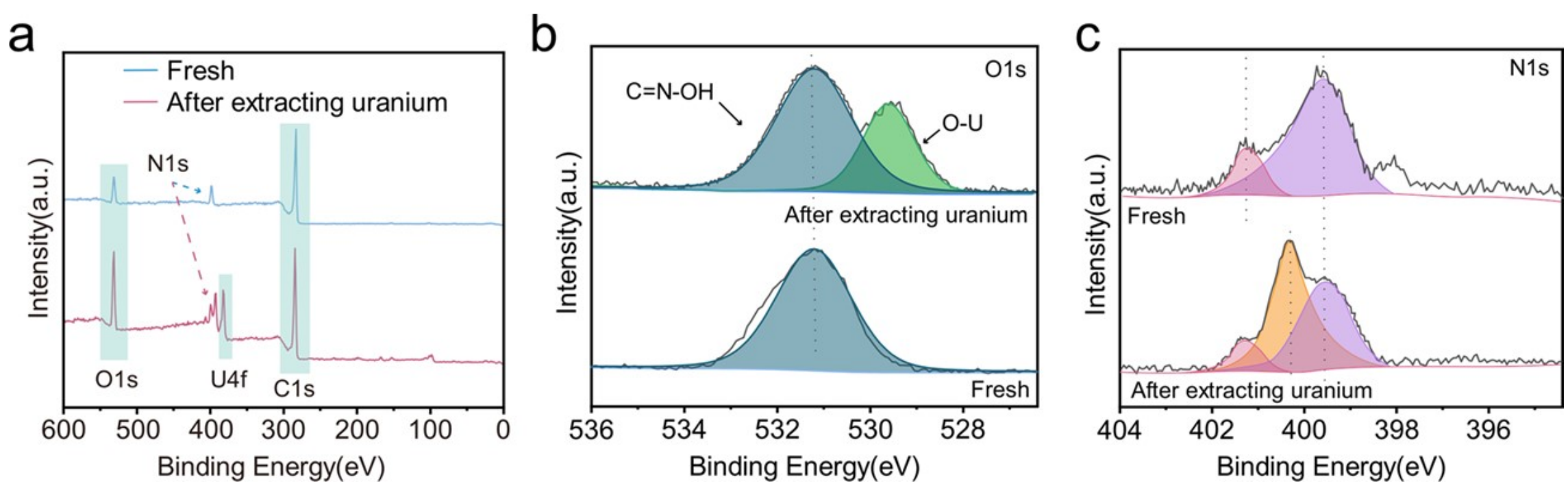

Figure S20. (a) XPS survey spectra of Ni-Por-COF-AO before and after uranium adsorption. (b) O1s XPS spectra of Ni-Por-COF-AO pre-/post-uranium extraction. (c) N1s XPS spectra of Ni-Por-COF-AO pre-/post-uranium extraction.

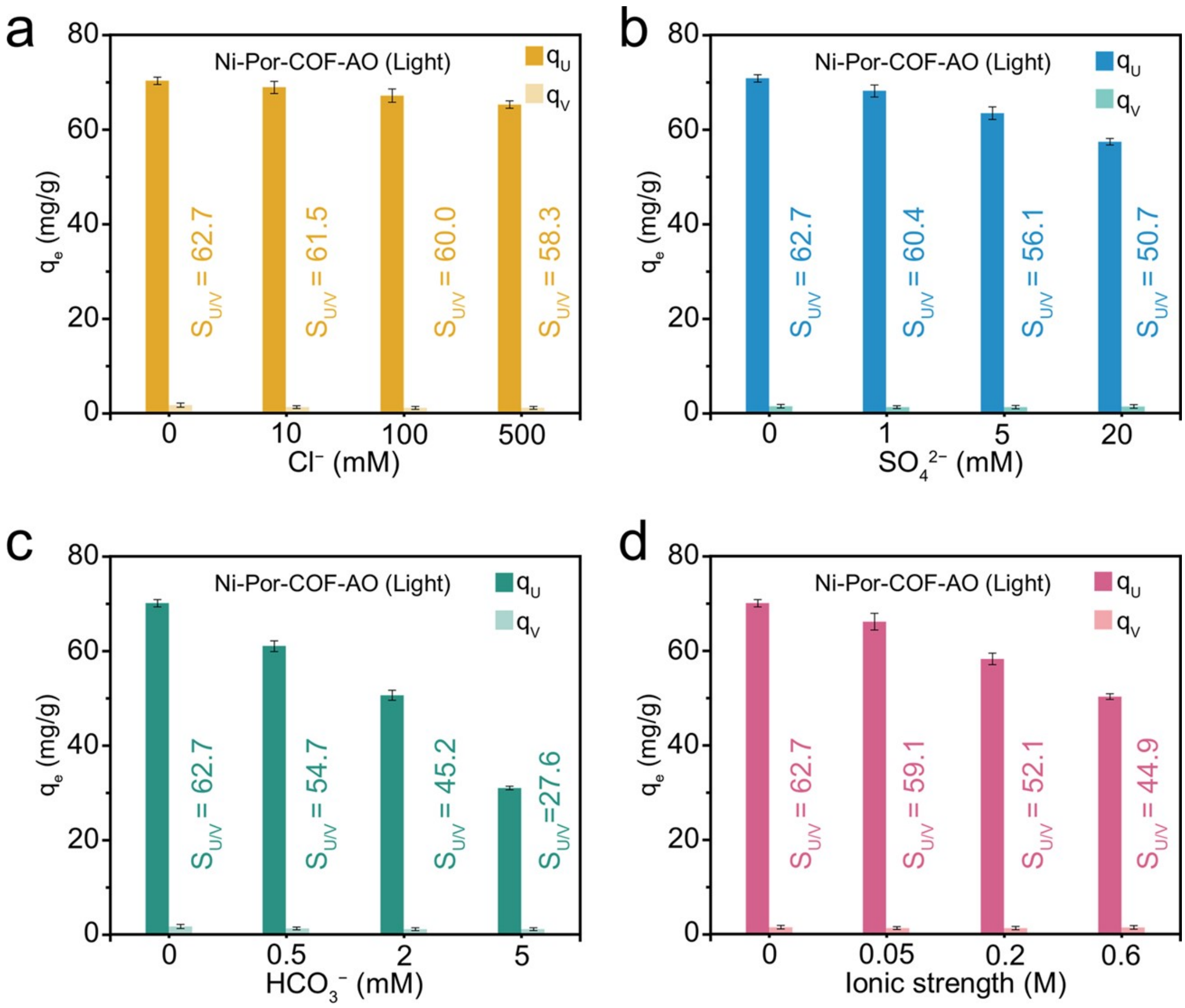

Figure S21. Effects of additional Cl^-, SO_4^{2-}, HCO_3^-, and ionic strength on the adsorption capacities of U and V and the corresponding $S_{U/V}$ values of Ni-Por-COF-AO under visible-light irradiation.

Table S1. Comparison of charge-transport descriptors and uranium-capture performance descriptors for Por-COF-AO, Co-Por-COF-AO, Ni-Por-COF-AO, and Cu-Por-COF-AO. The normalized 1/Rct, photocurrent, and τavg values are given relative to Por-COF-AO (set as 1.00).

Sample	Por-COF-AO	Co-Por-COF-AO	Ni-Por-COF-AO	Cu-Por-COF-AO
Rct (Ω)	4300	4100	4000	4700
normalized 1/Rct	1	1.05	1.08	0.91
Photocurrent (μA cm^{-2})	0.095	0.35	0.41	0.32
normalized photocurrent	1	3.68	4.32	3.37
τavg (ns)	3.52	3.97	5.72	3.68
normalized τavg	1	1.13	1.63	1.05
U uptake under light (mg g^{-1})	180	470	516.9	485
U uptake in dark (mg g^{-1})	120	310	357.7	325
Equilibrium capacity q_e (mg g^{-1})	220	480	759.1	545

K_d (mL g^{-1})	5.3×10^4	2.6×10^5	1.2×10^9	3.7×10^7

Table S2. Binding affinity of different adsorbents for uranium uptake from the spiked solution with an initial concentration range of 1–10 ppm**.**

Sample	V/m (mL/g)	K_d (mL g^{-1})	pH; concentration value	Uranium extraction method	Ref.
Ni-Por-COF-AO	30000	1.2×10^9	pH = 8-9; 5 ppm	Adsorption-photocatalysis	This work
Co-Por-COF-AO	30000	2.6×10^5	pH = 8-9; 5 ppm	Adsorption-photocatalysis	This work
Cu-Por-COF-AO	30000	3.7×10^7	pH = 8-9; 5 ppm	Adsorption-photocatalysis	This work
Por-COF-AO	30000	5.3×10^4	pH = 8-9; 5 ppm	Adsorption-photocatalysis	This work
MOF-808-4	20000	6.5×10^6	pH = 8-9; 5 ppm	adsorption	[2]
P-HOF	20000	8.42×10^5	pH = 6; 5 ppm	adsorption	[3]
MISS-PAF-1	5000	1.4×10^7	pH = 6.5;10 ppm	adsorption	[4]
COF-TpDb-AO	10000	3.6×10^8	pH = 6.0; 9.25 ppm	adsorption	[5]
HPC	2000	1.02×10^4	pH = 8; 10.3 ppm	adsorption	[6]
PIDO NF	330000	2.84×10^5	pH = 8; 8 ppm	adsorption	[7]
MIGPAF-13	10050	2.0×10^6	pH = 8; 1 ppm	electrocatalysis	[8]

Table S3. Maximum adsorption capacity and selectivity coefficient of different adsorbents.

Sample	Maximum adsorption capacity (mg g^{-1})	pH	$S_{U/V}$	concentration value	Ref.
Ni-Por-COF-AO	759.1	pH = 8-9	62.7	5 ppm	This work
UiO-66-AO	227.8	pH = 8-9	22.5	50 ppm	[9]
KMS	178	pH = 8-9	11.2	20 ppm	[10]
PEDOT@COF-AO	720	pH = 8.0	7.8	20 ppm	[11]
Ti_3C_2-AO-PA	108.5	pH = 8.0	2.1	3.3 ppm	[12]
PEI/SA-PAO	467.56	pH = 8-9	22	8 ppm	[13]
In–N_x–C–R	335	pH = 7	8.7	10 ppm	[14]
PAO-C Fs	52.88	pH = 5	15	14 ppm	[15]
Zn_2SnO_4/SnO_2	45	pH = 8-9	16.2	50 ppm	[16]
AO-PAN NFM	163.33	Ambient pH	18.8	3.3 ppm	[17]
PVPA/pine	111	pH = 7	15.2	100 ppm	[18]
DCQ-VP	33	pH = 7	30	6 ppm	[19]
SA-VP	28	pH = 7	25	10 ppm	[20]
COF-HHTF-AO	450	pH = 7	1.6	10 ppm	[21]

Table S4. Summary of adsorbents used for uranium extraction from seawater (pH in the range of 7.5~8.5).

Adsorbent	Time (day)	Method	Uranium capacity (mg/g)	Ref.
COF-PA	15	Chemical adsorption	2	[22]
38H-AO	56	Chemical adsorption	4.49	[23]
COF-HHTF-AO	25	Chemical adsorption	5.12	[21]
PEI/SA-PAO	/	Chemical adsorption	5.6	[13]
MOF-808-4	28	Chemical adsorption	5.83	[2]
n-Nx-C-R	1	Electrocatalysis	6.35	[14]
AI8-AO	56	Chemical adsorption	5.17	[23]
UUS-1/CdS	3	Photoreduction	7.03	[24]
P-HOF	13	Chemical adsorption	7.58	[3]
ECUT-COF-102	7	Chemical adsorption	9.2	[25]
PAN-PAO-OH-B-T	28	Chemical adsorption	9.66	[26]
CSMCRIHOF-1	5	Chemical adsorption	11	[27]
AF1-AO	56	Chemical adsorption	5.43	[23]
COF-R5	15	Chemical adsorption	11.3	[28]
GDT	30	Chemical adsorption	11.9	[29]
VA-PG	42	Photoreduction	13.63	[30]
MIGPAF-13-PM	56	Electrocatalysis	16	[8]
PPA@MISS-PAF-1	60	Electrocatalysis	16.5	[31]
Ni-Por-COF-AO	7	Adsorption-photocatalysis	21.2	This work

Table S5. Fractional atom coordinates for AA stacking modes of the Por-COF-AO unit cells.

Calculated unit cell	a=b=25.5478,c=3.6261, α=β=γ=90					
AA-stacked Por-COF-AO						
atoms	Type	*x*	*y*	*z*	Occupancy	Uiso
C1	C	-0.41704	-0.18603	1.50408	1.0	N/A
C2	C	-0.4731	-0.20611	1.45503	1.0	N/A
C3	C	-0.48307	-0.26451	1.41359	1.0	N/A
C4	C	-0.43663	-0.30236	1.39862	1.0	N/A
C5	C	-0.38	-0.28162	1.41546	1.0	N/A
C6	C	-0.37007	-0.22313	1.4764	1.0	N/A
C7	C	-0.44711	-0.3611	1.37819	1.0	N/A
C8	C	-0.40184	-0.39897	1.45087	1.0	N/A
C9	C	-0.34712	-0.38248	1.5825	1.0	N/A
C10	C	-0.31217	-0.43039	1.55845	1.0	N/A
C11	C	-0.34644	-0.47526	1.41737	1.0	N/A
N12	N	-0.4023	-0.45679	1.36536	1.0	N/A
C13	C	-0.32627	-0.52922	1.30875	1.0	N/A
C14	C	-0.26762	-0.53788	1.25496	1.0	N/A
C15	C	-0.36511	-0.57348	1.24337	1.0	N/A
N16	N	-0.42283	-0.57049	1.33622	1.0	N/A
C17	C	-0.44339	-0.62548	1.29656	1.0	N/A
C18	C	-0.39989	-0.66165	1.15521	1.0	N/A
C19	C	-0.35066	-0.62885	1.11939	1.0	N/A
C20	C	-0.49693	-0.70159	1.57994	1.0	N/A

C21	C	-0.54907	-0.72945	1.65747	1.0	N/A
C22	C	-0.54855	-0.78795	1.74536	1.0	N/A
C23	C	-0.49617	-0.8173	1.73654	1.0	N/A
C24	C	-0.44412	-0.78818	1.70851	1.0	N/A
C25	C	-0.44451	-0.72985	1.63087	1.0	N/A
C26	C	-0.49531	-0.6452	1.45041	1.0	N/A
C27	C	-0.23154	-0.49015	1.22868	1.0	N/A
C28	C	-0.17339	-0.49773	1.14587	1.0	N/A
C29	C	-0.15129	-0.55297	1.09717	1.0	N/A
C30	C	-0.187	-0.60105	1.12859	1.0	N/A
C31	C	-0.24544	-0.59342	1.21207	1.0	N/A
C32	C	-0.41061	-0.12888	1.60681	1.0	N/A
C33	C	-0.09218	-0.5586	1.0583	1.0	N/A
C34	C	-0.49457	-0.87628	1.72145	1.0	N/A
C35	C	-0.54292	-0.60954	1.49275	1.0	N/A
C36	C	-0.59508	-0.62592	1.66731	1.0	N/A
C37	C	-0.63068	-0.57848	1.65788	1.0	N/A
C38	C	-0.59948	-0.53386	1.48024	1.0	N/A
N39	N	-0.54526	-0.55275	1.37648	1.0	N/A
C40	C	-0.6193	-0.47773	1.4432	1.0	N/A
C41	C	-0.67574	-0.46587	1.55755	1.0	N/A
C42	C	-0.71366	-0.51167	1.61284	1.0	N/A
C43	C	-0.76584	-0.50277	1.80939	1.0	N/A
C44	C	-0.78136	-0.44756	1.93188	1.0	N/A
C45	C	-0.7458	-0.40063	1.84437	1.0	N/A
C46	C	-0.69277	-0.40981	1.65292	1.0	N/A
C47	C	-0.83486	-0.44047	2.11457	1.0	N/A
C48	C	-0.5818	-0.43357	1.33593	1.0	N/A
C49	C	-0.5989	-0.37835	1.2275	1.0	N/A
C50	C	-0.54942	-0.3455	1.19785	1.0	N/A
C51	C	-0.50295	-0.38082	1.29947	1.0	N/A
N52	N	-0.52251	-0.43596	1.367	1.0	N/A
C53	C	-0.05962	-0.50854	1.07795	1.0	N/A
C54	C	-0.44237	-0.90223	1.62578	1.0	N/A
C55	C	-0.86811	-0.48964	2.17402	1.0	N/A
C56	C	-0.46123	-0.09732	1.63683	1.0	N/A
C57	C	-0.92148	-0.49304	1.98441	1.0	N/A
C58	C	-0.94975	-0.54591	1.95702	1.0	N/A
C59	C	-0.94272	-0.44501	1.77891	1.0	N/A
C60	C	-0.44269	-0.96033	1.54465	1.0	N/A
C61	C	-0.39509	-0.99419	1.6377	1.0	N/A
C62	C	-0.49475	-0.98687	1.45958	1.0	N/A
C63	C	-0.46467	-0.05101	1.96606	1.0	N/A
C64	C	-0.41227	-0.02223	2.00147	1.0	N/A
C65	C	-0.51451	-0.01886	1.91153	1.0	N/A
H66	H	-0.93525	-0.58077	2.12347	1.0	N/A
H67	H	-0.54226	-0.0606	1.62862	1.0	N/A
H68	H	-0.35906	-0.96903	1.69113	1.0	N/A
C69	C	-0.01349	-0.5054	1.45391	1.0	N/A

C70	C	0.00806	-0.55495	1.51218	1.0	N/A
C71	C	0.01947	-0.46201	1.40915	1.0	N/A
H72	H	-0.00456	-0.41598	1.35486	1.0	N/A
H73	H	-0.50739	-0.17767	1.46525	1.0	N/A
H74	H	-0.52489	-0.27982	1.40725	1.0	N/A
H75	H	-0.34555	-0.30953	1.3851	1.0	N/A
H76	H	-0.32846	-0.20757	1.50469	1.0	N/A
H77	H	-0.33477	-0.34181	1.67583	1.0	N/A
H78	H	-0.26864	-0.43233	1.62542	1.0	N/A
H79	H	-0.40318	-0.70553	1.10657	1.0	N/A
H80	H	-0.31047	-0.64313	1.02766	1.0	N/A
H81	H	-0.58768	-0.70762	1.63427	1.0	N/A
H82	H	-0.58672	-0.80983	1.79738	1.0	N/A
H83	H	-0.40555	-0.81024	1.73264	1.0	N/A
H84	H	-0.40604	-0.70757	1.60386	1.0	N/A
H85	H	-0.2481	-0.44915	1.26341	1.0	N/A
H86	H	-0.14628	-0.4625	1.12695	1.0	N/A
H87	H	-0.17007	-0.64202	1.09821	1.0	N/A
H88	H	-0.272	-0.62874	1.24691	1.0	N/A
H89	H	-0.60475	-0.66545	1.79411	1.0	N/A
H90	H	-0.67188	-0.57597	1.7764	1.0	N/A
H91	H	-0.70257	-0.55244	1.5145	1.0	N/A
H92	H	-0.79336	-0.53699	1.86205	1.0	N/A
H93	H	-0.75852	-0.35941	1.92256	1.0	N/A
H94	H	-0.66553	-0.37557	1.59436	1.0	N/A
H95	H	-0.64083	-0.36418	1.1874	1.0	N/A
H96	H	-0.54746	-0.30238	1.12264	1.0	N/A
H97	H	-0.07608	-0.47009	0.96973	1.0	N/A
H98	H	-0.40426	-0.87913	1.6313	1.0	N/A
H99	H	-0.84979	-0.52615	2.29906	1.0	N/A
H100	H	-0.49931	-0.11447	1.52599	1.0	N/A
H101	H	-0.92301	-0.40539	1.81415	1.0	N/A
H102	H	-0.52653	-0.96706	1.28876	1.0	N/A
H103	H	-0.37944	-0.05236	2.06501	1.0	N/A
H104	H	-0.01638	-0.5915	1.65993	1.0	N/A
C105	C	0.14247	0.61431	1.18434	1.0	N/A
C106	C	0.64301	0.8942	0.7113	1.0	N/A
C107	C	0.93396	0.38725	0.05832	1.0	N/A
C108	C	0.45553	0.0913	0.78363	1.0	N/A
N109	N	0.12021	0.668	1.25413	1.0	N/A
N110	N	0.6955	0.9169	0.8153	1.0	N/A
N111	N	0.95929	0.33401	0.05674	1.0	N/A
N112	N	0.40655	0.05958	0.84526	1.0	N/A

Table S6. Fractional atom coordinates for AA stacking modes of the Co-Por-COF-AO unit cells.

Calculated unit cell a=b=25.5478,c=3.6261, α=β=γ=90						
AA-stacked Co-Por-COF-AO						
atoms	Type	*x*	*y*	*z*	Occupancy	Uiso

C1	C	-0.42322	-0.20272	1.80977	1.0	N/A
C2	C	-0.47571	-0.22226	1.65752	1.0	N/A
C3	C	-0.48011	-0.27789	1.50974	1.0	N/A
C4	C	-0.43225	-0.31396	1.52685	1.0	N/A
C5	C	-0.37855	-0.2928	1.647	1.0	N/A
C6	C	-0.37411	-0.237	1.79545	1.0	N/A
C7	C	-0.43955	-0.37251	1.47127	1.0	N/A
C8	C	-0.40236	-0.40985	1.67039	1.0	N/A
C9	C	-0.34966	-0.3941	1.84171	1.0	N/A
C10	C	-0.31654	-0.44357	1.86492	1.0	N/A
C11	C	-0.34969	-0.4881	1.70391	1.0	N/A
N12	N	-0.40472	-0.46843	1.6459	1.0	N/A
C13	C	-0.32683	-0.53506	1.50045	1.0	N/A
C14	C	-0.26786	-0.54415	1.49155	1.0	N/A
C15	C	-0.36223	-0.56976	1.26637	1.0	N/A
N16	N	-0.42117	-0.56673	1.26068	1.0	N/A
C17	C	-0.4404	-0.62159	1.19959	1.0	N/A
C18	C	-0.39467	-0.65492	1.05696	1.0	N/A
C19	C	-0.3452	-0.62227	1.10188	1.0	N/A
C20	C	-0.49472	-0.70289	1.42921	1.0	N/A
C21	C	-0.54872	-0.72465	1.53505	1.0	N/A
C22	C	-0.55376	-0.78193	1.64662	1.0	N/A
C23	C	-0.50438	-0.81575	1.65401	1.0	N/A
C24	C	-0.45136	-0.79493	1.52286	1.0	N/A
C25	C	-0.44674	-0.73841	1.39884	1.0	N/A
C26	C	-0.48721	-0.6444	1.40496	1.0	N/A
C27	C	-0.23084	-0.4976	1.54358	1.0	N/A
C28	C	-0.17363	-0.50297	1.42836	1.0	N/A
C29	C	-0.15361	-0.55534	1.27968	1.0	N/A
C30	C	-0.18862	-0.60396	1.27933	1.0	N/A
C31	C	-0.24636	-0.59824	1.3861	1.0	N/A
C32	C	-0.4202	-0.14658	1.94231	1.0	N/A
C33	C	-0.09706	-0.55824	1.1535	1.0	N/A
C34	C	-0.50663	-0.87235	1.76707	1.0	N/A
C35	C	-0.52247	-0.6097	1.63751	1.0	N/A
C36	C	-0.57374	-0.62767	1.81779	1.0	N/A
C37	C	-0.60696	-0.57888	1.87285	1.0	N/A
C38	C	-0.57522	-0.53277	1.71886	1.0	N/A
N39	N	-0.52058	-0.55116	1.63884	1.0	N/A
C40	C	-0.59964	-0.48409	1.54848	1.0	N/A
C41	C	-0.65776	-0.47388	1.61843	1.0	N/A
C42	C	-0.69297	-0.5206	1.7119	1.0	N/A
C43	C	-0.74849	-0.51148	1.85382	1.0	N/A
C44	C	-0.76925	-0.45563	1.893	1.0	N/A
C45	C	-0.73561	-0.40869	1.7729	1.0	N/A
C46	C	-0.67982	-0.41801	1.62589	1.0	N/A
C47	C	-0.82562	-0.44843	2.02445	1.0	N/A
C48	C	-0.56584	-0.44665	1.31995	1.0	N/A
C49	C	-0.58366	-0.39234	1.19306	1.0	N/A

C50	C	-0.53434	-0.35907	1.1535	1.0	N/A
C51	C	-0.48772	-0.39346	1.26674	1.0	N/A
N52	N	-0.50664	-0.44907	1.29964	1.0	N/A
C53	C	-0.06587	-0.50736	1.15241	1.0	N/A
C54	C	-0.45465	-0.90138	1.7767	1.0	N/A
C55	C	-0.85897	-0.49769	2.07824	1.0	N/A
C56	C	-0.47008	-0.11414	1.90827	1.0	N/A
C57	C	-0.91455	-0.49872	1.92166	1.0	N/A
C58	C	-0.94448	-0.55072	1.89783	1.0	N/A
C59	C	-0.93703	-0.44898	1.7446	1.0	N/A
C60	C	-0.45351	-0.95529	1.60852	1.0	N/A
C61	C	-0.40478	-0.99004	1.63922	1.0	N/A
C62	C	-0.50245	-0.97471	1.40881	1.0	N/A
C63	C	-0.46623	-0.06654	1.55136	1.0	N/A
C64	C	-0.41606	-0.0446	1.54968	1.0	N/A
C65	C	-0.5105	-0.03407	1.55029	1.0	N/A
H66	H	-0.92876	-0.58695	2.04273	1.0	N/A
H67	H	-0.54032	-0.05111	1.18904	1.0	N/A
H68	H	-0.37502	-0.97157	1.83387	1.0	N/A
C69	C	-0.01349	-0.5054	1.45391	1.0	N/A
C70	C	0.00806	-0.55495	1.51218	1.0	N/A
C71	C	0.01947	-0.46201	1.40915	1.0	N/A
H72	H	-0.00339	-0.4158	1.37324	1.0	N/A
H73	H	-0.51133	-0.19545	1.6566	1.0	N/A
H74	H	-0.51944	-0.29321	1.41067	1.0	N/A
H75	H	-0.3424	-0.31871	1.63139	1.0	N/A
H76	H	-0.3348	-0.22114	1.89345	1.0	N/A
H77	H	-0.33601	-0.3528	1.90721	1.0	N/A
H78	H	-0.27352	-0.44576	1.94621	1.0	N/A
H79	H	-0.39628	-0.69812	0.98292	1.0	N/A
H80	H	-0.3033	-0.63649	1.06331	1.0	N/A
H81	H	-0.5845	-0.69811	1.53698	1.0	N/A
H82	H	-0.59335	-0.79894	1.72807	1.0	N/A
H83	H	-0.41564	-0.82158	1.5189	1.0	N/A
H84	H	-0.40731	-0.72211	1.30942	1.0	N/A
H85	H	-0.24641	-0.45841	1.64194	1.0	N/A
H86	H	-0.14607	-0.46806	1.44812	1.0	N/A
H87	H	-0.1722	-0.64351	1.19344	1.0	N/A
H88	H	-0.27342	-0.63358	1.37885	1.0	N/A
H89	H	-0.5868	-0.66969	1.86441	1.0	N/A
H90	H	-0.64936	-0.57785	1.96965	1.0	N/A
H91	H	-0.67777	-0.56207	1.67616	1.0	N/A
H92	H	-0.77447	-0.54607	1.92902	1.0	N/A
H93	H	-0.75199	-0.36738	1.79124	1.0	N/A
H94	H	-0.65439	-0.38364	1.5376	1.0	N/A
H95	H	-0.62576	-0.37774	1.17822	1.0	N/A
H96	H	-0.53316	-0.31536	1.09633	1.0	N/A
H97	H	-0.08527	-0.46872	1.07716	1.0	N/A
H98	H	-0.41718	-0.88139	1.87237	1.0	N/A

H99	H	-0.8401	-0.53533	2.18071	1.0	N/A
H100	H	-0.50998	-0.13179	1.97345	1.0	N/A
H101	H	-0.91582	-0.41013	1.77654	1.0	N/A
H102	H	-0.53491	-0.94689	1.31976	1.0	N/A
H103	H	-0.38804	-0.0704	1.80604	1.0	N/A
H104	H	-0.01618	-0.59263	1.63474	1.0	N/A
C105	C	0.15038	0.60639	1.06188	1.0	N/A
C106	C	0.63291	0.87896	1.03179	1.0	N/A
C107	C	0.92973	0.38872	0.08814	1.0	N/A
C108	C	0.4408	0.09882	0.80648	1.0	N/A
N109	N	0.12683	0.66012	1.1017	1.0	N/A
N110	N	0.68499	0.90393	1.11987	1.0	N/A
N111	N	0.95589	0.33665	0.02481	1.0	N/A
N112	N	0.38933	0.07039	0.8439	1.0	N/A
Co113	Co	0.53667	0.49137	0.44708	1.0	N/A

Table S7. Fractional atom coordinates for AA stacking modes of the Ni-Por-COF-AO unit cells.

Calculated unit cell	a=b=25.5478,c=3.6261, α=β=γ=90					
AA-stacked Ni-Por-COF-AO						
atoms	Type	*x*	*y*	*z*	Occupancy	Uiso
C2	C	-0.4842	-0.2346	1.63699	1.0	N/A
C3	C	-0.4878	-0.289	1.64305	1.0	N/A
C4	C	-0.4461	-0.3203	1.52054	1.0	N/A
C5	C	-0.4005	-0.2954	1.39494	1.0	N/A
C6	C	-0.3965	-0.241	1.39312	1.0	N/A
C7	C	-0.4503	-0.3787	1.52361	1.0	N/A
C8	C	-0.4057	-0.4089	1.60744	1.0	N/A
C9	C	-0.3562	-0.3876	1.74311	1.0	N/A
C10	C	-0.3223	-0.4264	1.74171	1.0	N/A
C11	C	-0.3503	-0.4722	1.60524	1.0	N/A
N12	N	-0.4008	-0.4603	1.53248	1.0	N/A
C13	C	-0.3266	-0.5204	1.51922	1.0	N/A
C14	C	-0.268	-0.5244	1.51642	1.0	N/A
C15	C	-0.357	-0.5651	1.44077	1.0	N/A
N16	N	-0.4084	-0.57	1.52249	1.0	N/A
C17	C	-0.4204	-0.6202	1.44533	1.0	N/A
C18	C	-0.3774	-0.6465	1.30674	1.0	N/A
C19	C	-0.3369	-0.6113	1.30393	1.0	N/A
C20	C	-0.4731	-0.7024	1.52715	1.0	N/A
C21	C	-0.5189	-0.7273	1.404	1.0	N/A
C22	C	-0.5229	-0.7817	1.40205	1.0	N/A
C23	C	-0.4809	-0.8124	1.51837	1.0	N/A
C24	C	-0.4349	-0.7881	1.6413	1.0	N/A
C25	C	-0.4313	-0.7337	1.64742	1.0	N/A
C26	C	-0.4688	-0.644	1.52921	1.0	N/A
C27	C	-0.237	-0.4829	1.38814	1.0	N/A
C28	C	-0.1824	-0.4864	1.38902	1.0	N/A
C29	C	-0.1577	-0.532	1.51498	1.0	N/A

C30	C	-0.1882	-0.5736	1.64028	1.0	N/A
C31	C	-0.2427	-0.5697	1.64297	1.0	N/A
C32	C	-0.4334	-0.1547	1.50462	1.0	N/A
C33	C	-0.102	-0.5369	1.52073	1.0	N/A
C34	C	-0.4859	-0.868	1.51038	1.0	N/A
C35	C	-0.5132	-0.6138	1.61847	1.0	N/A
C36	C	-0.5624	-0.6351	1.75997	1.0	N/A
C37	C	-0.5963	-0.5963	1.76269	1.0	N/A
C38	C	-0.5686	-0.5504	1.62264	1.0	N/A
N39	N	-0.5183	-0.5624	1.544	1.0	N/A
C40	C	-0.5926	-0.5023	1.53735	1.0	N/A
C41	C	-0.6513	-0.4985	1.53868	1.0	N/A
C42	C	-0.6823	-0.5405	1.41957	1.0	N/A
C43	C	-0.7369	-0.5371	1.42198	1.0	N/A
C44	C	-0.7615	-0.4911	1.53982	1.0	N/A
C45	C	-0.7311	-0.449	1.65545	1.0	N/A
C46	C	-0.6765	-0.4528	1.65775	1.0	N/A
C47	C	-0.8173	-0.486	1.53774	1.0	N/A
C48	C	-0.5624	-0.4577	1.45318	1.0	N/A
C49	C	-0.5827	-0.4116	1.31571	1.0	N/A
C50	C	-0.5422	-0.3764	1.31152	1.0	N/A
C51	C	-0.4989	-0.4026	1.4467	1.0	N/A
N52	N	-0.5108	-0.4527	1.52846	1.0	N/A
C53	C	-0.0706	-0.4987	1.44341	1.0	N/A
C54	C	-0.4465	-0.8992	1.5564	1.0	N/A
C55	C	-0.8485	-0.5256	1.50854	1.0	N/A
C56	C	-0.4727	-0.1235	1.55479	1.0	N/A
C57	C	-0.9056	-0.519	1.49471	1.0	N/A
C58	C	-0.9385	-0.5625	1.53475	1.0	N/A
C59	C	-0.9271	-0.4694	1.43611	1.0	N/A
C60	C	-0.4529	-0.9561	1.55219	1.0	N/A
C61	C	-0.4087	-0.9886	1.5458	1.0	N/A
C62	C	-0.5031	-0.9781	1.55494	1.0	N/A
C63	C	-0.4662	-0.0665	1.55136	1.0	N/A
C64	C	-0.4161	-0.0446	1.54968	1.0	N/A
C65	C	-0.5105	-0.0341	1.55029	1.0	N/A
H66	H	-0.9198	-0.6025	1.5749	1.0	N/A
H67	H	-0.5509	-0.053	1.55828	1.0	N/A
H68	H	-0.3683	-0.9697	1.55019	1.0	N/A
C69	C	-0.0135	-0.5054	1.45391	1.0	N/A
C70	C	0.00806	-0.555	1.51218	1.0	N/A
C71	C	0.01947	-0.462	1.40915	1.0	N/A
H72	H	-0.0036	-0.4125	1.35695	1.0	N/A
H73	H	-0.5188	-0.21	1.73408	1.0	N/A
H74	H	-0.525	-0.3086	1.74845	1.0	N/A
H75	H	-0.366	-0.3196	1.29252	1.0	N/A
H76	H	-0.3588	-0.2217	1.29408	1.0	N/A
H77	H	-0.3534	-0.3447	1.82438	1.0	N/A
H78	H	-0.2803	-0.4195	1.8369	1.0	N/A

H79	H	-0.3811	-0.6892	1.22281	1.0	N/A
H80	H	-0.2941	-0.6137	1.21692	1.0	N/A
H81	H	-0.5535	-0.703	1.30374	1.0	N/A
H82	H	-0.5606	-0.8011	1.30524	1.0	N/A
H83	H	-0.4003	-0.8127	1.73662	1.0	N/A
H84	H	-0.394	-0.7141	1.75102	1.0	N/A
H85	H	-0.2563	-0.4457	1.28146	1.0	N/A
H86	H	-0.1578	-0.4522	1.2872	1.0	N/A
H87	H	-0.1686	-0.6109	1.74102	1.0	N/A
H88	H	-0.2674	-0.6036	1.7489	1.0	N/A
H89	H	-0.565	-0.678	1.8414	1.0	N/A
H90	H	-0.638	-0.6032	1.86312	1.0	N/A
H91	H	-0.6631	-0.5781	1.31911	1.0	N/A
H92	H	-0.7615	-0.5718	1.32799	1.0	N/A
H93	H	-0.7507	-0.4112	1.74846	1.0	N/A
H94	H	-0.6518	-0.4185	1.75726	1.0	N/A
H95	H	-0.6257	-0.4093	1.23354	1.0	N/A
H96	H	-0.5491	-0.335	1.20837	1.0	N/A
H97	H	-0.0871	-0.4589	1.36528	1.0	N/A
H98	H	-0.4056	-0.8825	1.60151	1.0	N/A
H99	H	-0.8318	-0.5669	1.49298	1.0	N/A
H100	H	-0.5135	-0.1402	1.60303	1.0	N/A
H101	H	-0.9004	-0.434	1.40945	1.0	N/A
H102	H	-0.539	-0.9517	1.56094	1.0	N/A
H103	H	-0.3801	-0.071	1.55137	1.0	N/A
H104	H	-0.0187	-0.5903	1.54226	1.0	N/A
C105	C	0.16956	0.55619	0.826	1.0	N/A
C106	C	0.62583	0.84904	0.58036	1.0	N/A
C107	C	0.91278	0.40504	0.48773	1.0	N/A
C108	C	0.45652	0.13639	0.62938	1.0	N/A
N109	N	0.15667	0.59766	1.10838	1.0	N/A
N110	N	0.68395	0.8527	0.65442	1.0	N/A
N111	N	0.9273	0.348	0.45532	1.0	N/A
N112	N	0.39979	0.1407	0.74678	1.0	N/A
Ni113	Ni	0.54382	0.48934	0.61332	1.0	N/A

Table S8. Fractional atom coordinates for AA stacking modes of the Cu-Por-COF-AO unit cells.

Calculated unit cell	a=b=25.5478,c=3.6261, α=β=γ=90					
AA-stacked Cu-Por-COF-AO						
atoms	Type	*x*	*y*	*z*	Occupancy	Uiso
C1	C	-0.404	-0.20417	1.84046	1.0	N/A
C2	C	-0.46114	-0.22172	1.79469	1.0	N/A
C3	C	-0.47337	-0.27709	1.66298	1.0	N/A
C4	C	-0.42837	-0.31566	1.59824	1.0	N/A
C5	C	-0.37103	-0.29812	1.6439	1.0	N/A
C6	C	-0.35868	-0.24197	1.76334	1.0	N/A
C7	C	-0.44096	-0.3726	1.50865	1.0	N/A
C8	C	-0.40214	-0.41503	1.61803	1.0	N/A

C9	C	-0.34513	-0.40449	1.72926	1.0	N/A
C10	C	-0.31438	-0.45402	1.644	1.0	N/A
C11	C	-0.35355	-0.49356	1.48501	1.0	N/A
N12	N	-0.40816	-0.47209	1.52628	1.0	N/A
C13	C	-0.3383	-0.53822	1.22869	1.0	N/A
C14	C	-0.28015	-0.55006	1.17314	1.0	N/A
C15	C	-0.38074	-0.57037	1.03804	1.0	N/A
N16	N	-0.43868	-0.5659	1.12297	1.0	N/A
C17	C	-0.46029	-0.62078	1.09478	1.0	N/A
C18	C	-0.42048	-0.65492	0.88772	1.0	N/A
C19	C	-0.37014	-0.62328	0.85456	1.0	N/A
C20	C	-0.5053	-0.70221	1.42085	1.0	N/A
C21	C	-0.55356	-0.72464	1.61866	1.0	N/A
C22	C	-0.55197	-0.78039	1.77181	1.0	N/A
C23	C	-0.50243	-0.81346	1.71841	1.0	N/A
C24	C	-0.45721	-0.79359	1.47673	1.0	N/A
C25	C	-0.45888	-0.73787	1.32342	1.0	N/A
C26	C	-0.50077	-0.64318	1.36304	1.0	N/A
C27	C	-0.24105	-0.50501	1.21617	1.0	N/A
C28	C	-0.18244	-0.5157	1.18948	1.0	N/A
C29	C	-0.16265	-0.57183	1.14317	1.0	N/A
C30	C	-0.20143	-0.61726	1.09127	1.0	N/A
C31	C	-0.26069	-0.6059	1.09238	1.0	N/A
C32	C	-0.39417	-0.14708	1.93483	1.0	N/A
C33	C	-0.10354	-0.58004	1.16261	1.0	N/A
C34	C	-0.49772	-0.86669	1.90447	1.0	N/A
C35	C	-0.53172	-0.60654	1.6139	1.0	N/A
C36	C	-0.5795	-0.62246	1.84486	1.0	N/A
C37	C	-0.61014	-0.57264	1.92578	1.0	N/A
C38	C	-0.58067	-0.52748	1.73934	1.0	N/A
N39	N	-0.52861	-0.54781	1.60423	1.0	N/A
C40	C	-0.60611	-0.47659	1.60864	1.0	N/A
C41	C	-0.66362	-0.4652	1.70882	1.0	N/A
C42	C	-0.69887	-0.51054	1.83311	1.0	N/A
C43	C	-0.75632	-0.50051	1.9309	1.0	N/A
C44	C	-0.7796	-0.4458	1.88163	1.0	N/A
C45	C	-0.74448	-0.39977	1.76806	1.0	N/A
C46	C	-0.68608	-0.40944	1.68544	1.0	N/A
C47	C	-0.83912	-0.43978	1.9106	1.0	N/A
C48	C	-0.57354	-0.4379	1.38112	1.0	N/A
C49	C	-0.58958	-0.38089	1.31282	1.0	N/A
C50	C	-0.53917	-0.34912	1.28596	1.0	N/A
C51	C	-0.49351	-0.38752	1.33478	1.0	N/A
N52	N	-0.51486	-0.44259	1.32383	1.0	N/A
C53	C	-0.06974	-0.53071	1.13597	1.0	N/A
C54	C	-0.44664	-0.89753	1.85694	1.0	N/A
C55	C	-0.87123	-0.4888	2.00215	1.0	N/A
C56	C	-0.44256	-0.11396	2.01718	1.0	N/A
C57	C	-0.92288	-0.49931	1.79971	1.0	N/A

C58	C	-0.95078	-0.55199	1.85388	1.0	N/A
C59	C	-0.9433	-0.45989	1.5136	1.0	N/A
C60	C	-0.44515	-0.95605	1.94115	1.0	N/A
C61	C	-0.39556	-0.98794	1.85639	1.0	N/A
C62	C	-0.49448	-0.98468	2.0715	1.0	N/A
C63	C	-0.45055	-0.06273	1.81047	1.0	N/A
C64	C	-0.40975	-0.03378	1.59981	1.0	N/A
C65	C	-0.49722	-0.04332	1.99993	1.0	N/A
H66	H	-0.93666	-0.58031	2.07502	1.0	N/A
H67	H	-0.53114	-0.06776	2.10899	1.0	N/A
H68	H	-0.37911	-1.0039	2.126	1.0	N/A
C69	C	-0.01533	-0.52798	1.31004	1.0	N/A
C70	C	0.00374	-0.56725	1.59848	1.0	N/A
C71	C	0.01113	-0.47508	1.25886	1.0	N/A
H72	H	-0.00374	-0.44636	1.04279	1.0	N/A
H73	H	-0.49433	-0.19311	1.85197	1.0	N/A
H74	H	-0.51566	-0.29011	1.62625	1.0	N/A
H75	H	-0.33783	-0.32652	1.58416	1.0	N/A
H76	H	-0.31641	-0.22835	1.79213	1.0	N/A
H77	H	-0.32798	-0.36555	1.82088	1.0	N/A
H78	H	-0.27003	-0.45859	1.65541	1.0	N/A
H79	H	-0.42506	-0.69815	0.82098	1.0	N/A
H80	H	-0.3304	-0.63872	0.76203	1.0	N/A
H81	H	-0.58994	-0.69961	1.65765	1.0	N/A
H82	H	-0.58676	-0.79676	1.93091	1.0	N/A
H83	H	-0.42196	-0.81968	1.42068	1.0	N/A
H84	H	-0.42446	-0.72213	1.1584	1.0	N/A
H85	H	-0.25576	-0.46362	1.26564	1.0	N/A
H86	H	-0.15326	-0.48223	1.21516	1.0	N/A
H87	H	-0.18646	-0.65879	1.05112	1.0	N/A
H88	H	-0.28995	-0.63891	1.04888	1.0	N/A
H89	H	-0.59189	-0.66386	1.91886	1.0	N/A
H90	H	-0.6493	-0.57012	2.07469	1.0	N/A
H91	H	-0.68307	-0.552	1.83992	1.0	N/A
H92	H	-0.7823	-0.53421	2.02426	1.0	N/A
H93	H	-0.76164	-0.35888	1.73975	1.0	N/A
H94	H	-0.65968	-0.37529	1.61017	1.0	N/A
H95	H	-0.63096	-0.36443	1.32273	1.0	N/A
H96	H	-0.53623	-0.30473	1.2805	1.0	N/A
H97	H	-0.08675	-0.49432	1.00032	1.0	N/A
H98	H	-0.41072	-0.8775	1.73533	1.0	N/A
H99	H	-0.85354	-0.5205	2.1843	1.0	N/A
H100	H	-0.47595	-0.13101	2.18744	1.0	N/A
H101	H	-0.9237	-0.42018	1.48263	1.0	N/A
H102	H	-0.52858	-0.96298	2.20299	1.0	N/A
H103	H	-0.37347	-0.05893	1.55566	1.0	N/A
H104	H	-0.01654	-0.60659	1.63289	1.0	N/A
C105	C	0.13403	0.6119	0.80518	1.0	N/A
C106	C	0.66055	0.8775	0.89519	1.0	N/A

C107	C	0.92066	0.36553	0.22209	1.0	N/A
C108	C	0.45646	0.11166	1.13352	1.0	N/A
N109	N	0.10761	0.66255	0.70191	1.0	N/A
N110	N	0.71419	0.90161	0.85684	1.0	N/A
N111	N	0.94444	0.3122	0.28019	1.0	N/A
N112	N	0.41151	0.09061	1.35923	1.0	N/A
Cu113	Cu	0.527	0.49328	0.37212	1.0	N/A

References

[1] Cui W-R, Li F-F, Xu R-H, Zhang C-R, Chen X-R, Yan R-H*, et al.* Regenerable Covalent Organic Frameworks for Photo-enhanced Uranium Adsorption from Seawater. *Angew. Chem. Int. Ed* **2020**, 59(40)**:** 17684-17690.

[2] Zhao Z, Lei R, Zhang Y, Cai T, Han B. Defect controlled MOF-808 for seawater uranium capture with high capacity and selectivity. *Journal of Molecular Liquids* **2022**, 367.

[3] Shao C, Tuo K, Hou W, Liang C, Li Y, Sun Y*, et al.* Construction of a phosphonate-based hydrogen-bonded organic framework for efficient extraction of uranium from seawater. *Colloids and Surfaces A: Physicochemical and Engineering Aspects* **2025**, 718.

[4] Yuan Y, Meng Q, Faheem M, Yang Y, Li Z, Wang Z*, et al.* A Molecular Coordination Template Strategy for Designing Selective Porous Aromatic Framework Materials for Uranyl Capture. *ACS Central Science* **2019**, 5(8)**:** 1432-1439.

[5] Sun Q, Aguila B, Earl LD, Abney CW, Wojtas L, Thallapally PK*, et al.* Covalent Organic Frameworks as a Decorating Platform for Utilization and Affinity Enhancement of Chelating Sites for Radionuclide Sequestration. *Advanced Materials* **2018**, 30(20).

[6] Zhu J, Liu Q, Liu J, Chen R, Zhang H, Yu J*, et al.* Novel Ion-Imprinted Carbon Material Induced by Hyperaccumulation Pathway for the Selective Capture of Uranium. *ACS Applied Materials & Interfaces* **2018**, 10(34)**:** 28877-28886.

[7] Wang D, Song J, Wen J, Yuan Y, Liu Z, Lin S*, et al.* Significantly Enhanced Uranium Extraction from Seawater with Mass Produced Fully Amidoximated Nanofiber Adsorbent. *Advanced Energy Materials* **2018**, 8(33).

[8] Wang Z, Ma R, Meng Q, Yang Y, Ma X, Ruan X*, et al.* Constructing Uranyl-Specific Nanofluidic Channels for Unipolar Ionic Transport to Realize Ultrafast Uranium Extraction. *Journal of the American Chemical Society* **2021**, 143(36)**:** 14523-14529.

[9] Liu J-m, Yin X-h, Liu T. Amidoxime-functionalized metal-organic frameworks UiO-66 for U(VI) adsorption from aqueous solution. *Journal of the Taiwan Institute of Chemical Engineers* **2019**, 95**:** 416-423.

[10] Naggar AH, Alenezy EK, Bakr A-SA, Khairallah HA, Dhmees AS. Sustainable and cost-effective fabrication of kaolinite/MoS2 nanocomposite for enhanced uranium sorption: Experimental and mathematical modeling insights. *Inorganic Chemistry Communications* **2025**, 180.

[11] Song Y, Hou L, Lan PC, Xing Z, Sun Q, Lv J*, et al.* Creating electrochemical accessibility in covalent organic frameworks for uranium extraction via electrodeposition. *Nature Communications* **2025**, 16(1).

[12] Zhang D, Liu L, Zhao B, Wang X, Pang H, Yu S. Highly efficient extraction of uranium from seawater by polyamide and amidoxime co-functionalized MXene. *Environmental Pollution* **2023**, 317.

[13] Lin Q, Liu X, Zhang X, Ye Z, Fang J, Yang Z*, et al.* PEI/SA-PAO double crosslinked network gel spheres composites for uranium extraction from seawater. *Separation and Purification Technology* **2025**, 376.

[14] Liu X, Xie Y, Hao M, Chen Z, Yang H, Waterhouse GIN*, et al.* Highly Efficient Electrocatalytic Uranium Extraction from Seawater over an Amidoxime‐Functionalized In‐N‐C Catalyst. *Advanced Science* **2022**, 9(23).

[15] Wang Y, Zhang Y, Li Q, Li Y, Cao L, Li W. Amidoximated cellulose fiber membrane for uranium extraction from simulated seawater. *Carbohydrate Polymers* **2020**, 245.

[16] Cai Y, Zhang Y, Lv Z, Zhang S, Gao F, Fang M*, et al.* Highly efficient uranium extraction by a piezo catalytic reduction-oxidation process. *Applied Catalysis B: Environmental* **2022**, 310.

[17] Chen X, Wan C, Yu R, Meng L, Wang D, Duan T*, et al.* Fabrication of amidoximated polyacrylonitrile nanofibrous membrane by simultaneously biaxial stretching for uranium extraction from seawater. *Desalination* **2020**, 486.

[18] Zhang X, Gao Q, Yuan Y, Shao D. Application of Natural Antibacterial Plants in the Extraction of Uranium from Seawater. *ACS Applied Bio Materials* **2024**, 7(8)**:** 5461-5469.

[19] Yue Y, Mayes RT, Kim J, Fulvio PF, Sun XG, Tsouris C*, et al.* Seawater Uranium Sorbents: Preparation from a Mesoporous Copolymer Initiator by Atom‐Transfer Radical Polymerization. *Angewandte Chemie International Edition* **2013**, 52(50)**:** 13458-13462.

[20] Metilda P, Gladis JM, Venkateswaran G, Prasada Rao T. Investigation of the role of chelating ligand in the synthesis of ion-imprinted polymeric resins on the selective enrichment of uranium(VI). *Analytica Chimica Acta* **2007**, 587(2)**:** 263-271.

[21] Cheng G, Zhang A, Zhao Z, Chai Z, Hu B, Han B*, et al.* Extremely stable amidoxime functionalized covalent organic frameworks for uranium extraction from seawater with high efficiency and selectivity. *Sci. Bull.* **2021**, 66(19)**:** 1994-2001.

[22] Xia T, Liu J, Zhang J, Tang W, Xue J, Hao L*, et al.* Rod-structured phytic acid-functionalized covalent organic frameworks for

enhanced uranium extraction from seawater. *Desalination* **2025**, 616.

[23] Kuo L-J, Janke CJ, Wood JR, Strivens JE, Das S, Oyola Y*, et al.* Characterization and Testing of Amidoxime-Based Adsorbent Materials to Extract Uranium from Natural Seawater. *Industrial & Engineering Chemistry Research* **2015**, 55(15)**:** 4285-4293.

[24] Wang M, Feng L, Luo G, Feng T, Zhao S, Wang H, Shi S, Liu T, Fu Q, Li J, Wang N, Yuan Y. Ultrafast extraction of uranium from seawater using photosensitized biohybrid system with bioinspired cascaded strategy. Journal of Hazardous Materials. 2023, 445: 130620.

[25] Huang Z, Liu Y, Xie X, Yang Y, Yu Z, Luo F. Creating precise uranyl-targeted site through sulphonation within mesoporous covalent organic framework enabling high U/V selectivity and rapid uranium extraction from seawater. *Chemical Engineering Journal* **2025**, 520.

[26] Gui Y, Li Q, Su X, Ruan H, Wang J, Yan Y*, et al.* Anti-biofouling amidoxime polyacrylonitrile composite fibers modified with phosphate and guanidine for efficient and selective uranium extraction from seawater. *Separation and Purification Technology* **2025**, 378.

[27] Kaushik A, Marvaniya K, Kulkarni Y, Bhatt D, Bhatt J, Mane M*, et al.* Large-area self-standing thin film of porous hydrogen-bonded organic framework for efficient uranium extraction from seawater. *Chem* **2022**, 8(10)**:** 2749-2765.

[28] Xie Y, Wu Y, Liu X, Hao M, Chen Z, Waterhouse GIN*, et al.* Rational design of cooperative chelating sites on covalent organic frameworks for highly selective uranium extraction from seawater. *Cell Reports Physical Science* **2023**, 4(1).

[29] Li Y, Zheng YJ, Ahamd Z, Zhu LX, Yang JJ, Chen JP*, et al.* Strategies for designing highly efficient adsorbents to capture uranium from seawater. *Coord. Chem. Rev.* **2023**, 491**:** 215234.

[30] Liu T, Zhang R, Chen M, Liu Y, Xie Z, Tang S*, et al.* Vertically Aligned Polyamidoxime/Graphene Oxide Hybrid Sheets' Membrane for Ultrafast and Selective Extraction of Uranium from Seawater. *Advanced Functional Materials* **2021**, 32(14).

[31] Wang Z, Meng Q, Ma R, Wang Z, Yang Y, Sha H*, et al.* Constructing an Ion Pathway for Uranium Extraction from Seawater. *Chem* **2020**, 6(7)**:** 1683-1691.
